# Metal-free syntheses of new azocines *via* addition reactions of enaminones with acenaphthoquinone followed by oxidative cleavages of the corresponding vicinal diols†

**DOI:** 10.1039/d0ra02852e

**Published:** 2020-05-29

**Authors:** S. Hekmat Mousavi, Mohammad Reza Mohammadizadeh, Satoru Arimitsu, Dariush Saberi, Samira Poorsadeghi, Kojya Genta

**Affiliations:** Department of Chemistry, Faculty of Sciences, Persian Gulf University Bushehr 75169 Iran mrmohamadizadeh@pgu.ac.ir +98-771-4541494; Department of Chemistry, Biology and Marine Science, Faculty of Science, University of the Ryukyus 1-Senbaru, Nishihara Nakagami Okinawa 903-0213 Japan; Marine Chemistry Department, Faculty of Marine Science and Technology, Persian Gulf University Bushehr 75169 Iran; Center for Research Advancement and Collaboration, University of the Ryukyus Senbaru 1, Nishihara Okinawa 903-0213 Japan

## Abstract

A one-pot, clean and green procedure is described for the syntheses of new azocine derivatives *via* addition reactions of enaminones with acenaphthoquinone followed by periodic acid-mediated oxidative cleavages of the corresponding vicinal diols. Various derivatives of azocine were prepared and well characterized. The excellent yields, simple synthesis procedure, lack of a need to carry out any tedious work-up and column chromatography, metal-free catalysis, and mild reaction conditions are important features of this protocol.

## Abstract 2

One-pot metal-free periodic acid-mediated syntheses of azocine rings with high yields under mild reaction conditions *via* the addition reactions of enaminones and acenaphthoquinone.

## Graphical abstract

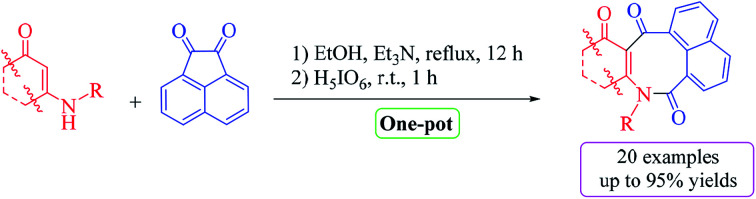

## Introduction

Eight-membered azocine rings are N-heterocyclic compounds that play a pivotal role in the structures of various important natural products as well as precursors in the syntheses of biologically active compounds.^1^ Azocine derivatives have also found applications as therapeutic agents, such as antitussives, antihypertensives, analgesics, nasal decongestants, and antimalarials.^2^ These compounds are also structurally attractive, as they show interesting conformations. Because of unfavorable entropy and enthalpy factors, the azocine ring system is generally difficult to obtain,^3^ and relatively few methods are available for its preparation. However, there have been several general approaches used to construct azocine rings, including cycloaddition,^4^ the fragmentation reaction,^5^ Dieckmann cyclization,^6^ tandem hydroboration reactions,^7^ the Michael reaction,^8^ intramolecular Heck reactions,^9^ microwave (MW)- and photo-assisted reactions,^10^ ring-expansion reactions,^11^ and ring-closing metathesis.^12^ Recently, Soldatova *et al.* reported a new pathway for azocine derivative synthesis, with this pathway involving the reaction of an activated acetylene with a β-amino ketone.^13^ Most of the methods developed for azocine synthesis use expensive and inaccessible raw materials. In addition, some of the problems associated with the previous methods include the employment of metals as catalysts, harsh reaction conditions, poor product efficiency and tedious work-up. The interesting structures and important biological properties of these compounds constitute the driving force for the development of greener methods for synthesizing these compounds. In 2014, our research group developed a novel procedure for the synthesis of new azocine derivatives, with this procedure involving reactions of acenaphthoquinone with 6-aminouracil derivatives in the presence of lead(iv) acetate.^14^ Although this method is interesting for forming an eight-membered azocine ring, both in terms of starting from relatively simple raw materials and mild reaction conditions and product efficiency, the use of toxic lead metal as a catalyst has reduced its popularity. In the present study, in continuation of our studies on the oxidative cleavages of cyclic vicinal diols aimed at synthesizing potentially biologically active heterocycles,^15^ we developed a facile and green procedure for the synthesis of new azocine derivatives. The addition reactions of enaminones, prepared from reactions between simple and commercially available β-diketones and amines, with acenaphthoquinone followed by the metal-free H_5_IO_6_-mediated oxidative cleavage of the corresponding vicinal diols resulted in the azocine derivatives with excellent yields under mild reaction conditions.

## Results and discussion

**Scheme 1:**
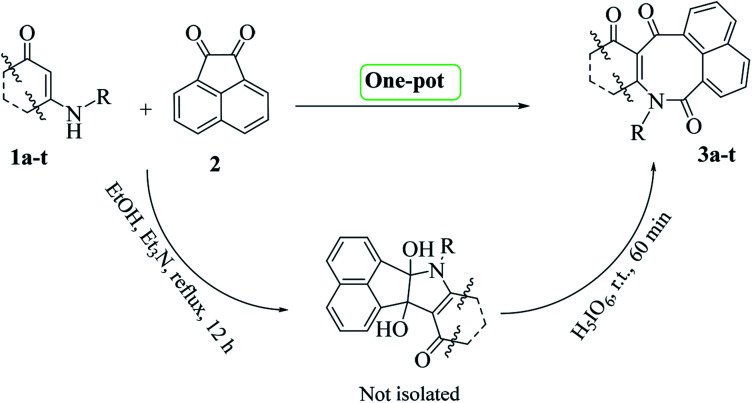
Procedure for one-pot syntheses of azocines 3a–t

Initially, according to a previously reported procedure,^16^ enaminone derivatives 1a–t were synthesized *via* I_2_-mediated condensation reactions of amines and 1,3-dicarbonyls (for details see General procedure). The synthesized enaminones 1a–t were then reacted with acenaphthoquinone 2 and converted to the corresponding azocines 3a–t in two steps (addition and oxidative-cleavage steps) as a one-pot reaction (Scheme 1).

Initial studies were done on the synthesis of product 3b. In the addition step, which was carried out in ethanol and in the presence of Et_3_N serving as a base, all of the starting materials were consumed after twelve hours of reaction time (Scheme 1, step 1). Based on our previous studies on the addition reactions of various dinucleophiles and diketones,^14,15*a*–*d*^ it was predictable that vicinal diols would be the reaction products at this stage. With this precedence in mind and without carrying out any separation procedure at this stage, the corresponding intermediate was introduced into the second phase of the reaction—and by adding H_5_IO_6_ into the vessel, the reaction was allowed to continue for another one hour at room temperature. After now carrying out separation and purification of the final product, spectral data taken from this product confirmed it to be structure 3b. The ^1^H-NMR spectrum of 3b in DMSO-*d*_6_ exhibited three sharp singlets, at *δ* = 0.62, 0.84, and 3.83, attributed to the methyl groups. It also exhibited one sharp doublet signal (^3^*J* = 16.5 Hz) at 2.03 ppm, together with a multiplet signal in the region 2.12–2.24 ppm and a sharp doublet signal (^3^*J* = 17.6 Hz) at 2.33 ppm related to the methylene protons. Ten aromatic hydrogens were also indicated by the presence of three doublets (*δ* = 7.14, ^3^*J* = 8.5 Hz), (*δ* = 7.41, ^3^*J* = 8.5 Hz), and (*δ* = 7.91, ^3^*J* = 6.9 Hz), and one triplet (*δ* = 8.18, ^3^*J* = 8.6 Hz) along with a multiplet at *δ* = 7.57–7.80 ppm. The ^1^H- decoupled ^13^C-NMR spectrum of 3b showed 24 distinct signals in agreement with the proposed structure. The high-resolution mass spectrum of 3b displayed an [M + H]^+^ peak at *m*/*z* = 426.1700, in agreement with the proposed structure.

**Fig. 1:**
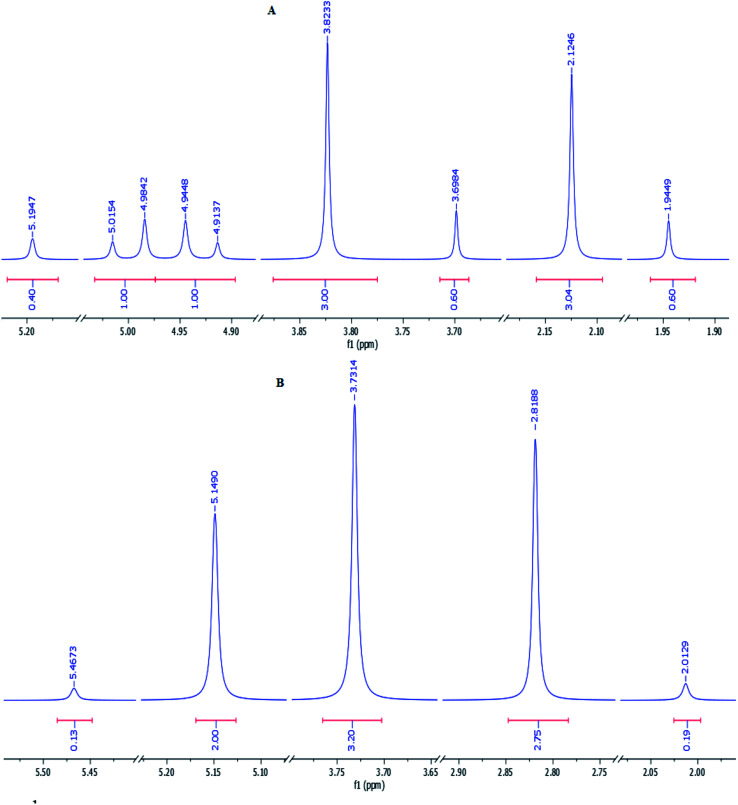
^1^H-NMR spectra of product 3s recorded at room temperature (A) and 80 °C (B). The aliphatic region of each spectrum is shown.

Subsequently, in order to extend the scope of this reaction, the wide range of enaminones 1a–m, synthesized *via* the reactions of cyclic β-dicarbonyls such as dimedone and 1,3-cyclohexadione with aromatic amines, were reacted with acenaphthoquinone to produce azocines, whose structures 3a–m and yields are shown in Table 1. As can be seen in this table, the products 3a–m were obtained with high efficiencies, indicating that the type, position, and number of substituents on the N-linked aromatic ring had no significant effect on the reaction rates. Moreover, the enaminones 1n–q composed of aliphatic amines such as ethyl and methyl amines as well as polycyclic amines, that is, 1-naphthylamine, were also subjected to the reaction conditions and the corresponding azocines 3n–q were isolated in high yields. And enaminones 1r–t, which were synthesized from the reactions of linear 1,3-dicarbonyls such as benzyl acetoacetate and isobutyl acetoacetate, were also successfully subjected to this reaction and their products 3r–t were obtained in good yields. It is worth noting that in contrast to the ^1^H- and ^13^C-NMR spectra of compounds 3a–q, which indicated the presence of one conformer for each compound, the spectra of products 3r–t each showed two different conformers; this result may have been due to the structural flexibilities of 3r–t being relatively high, and greater than those of compounds 3a–q, which have cyclic enaminone moieties. For product 3s, the ratio of its conformers was calculated from the ^1^H-NMR spectrum to be 83 : 17. The methylene protons of the major conformer resonated as two distinct doublets due to their diastereotopic nature, but they displayed a simple pattern in the minor conformer (Fig. 1). Surprisingly, the two distinct doublets of methylene protons of the major conformer of 3s at room temperature converted to a sharp singlet when its ^1^H-NMR spectrum was recorded at 80 °C. Moreover, the ratio of conformers was determined from the spectrum recorded at 80 °C to be 94 : 16 (Fig. 1).

**Table 1:** The structures of the synthesized azocine derivatives 3a–t^a^

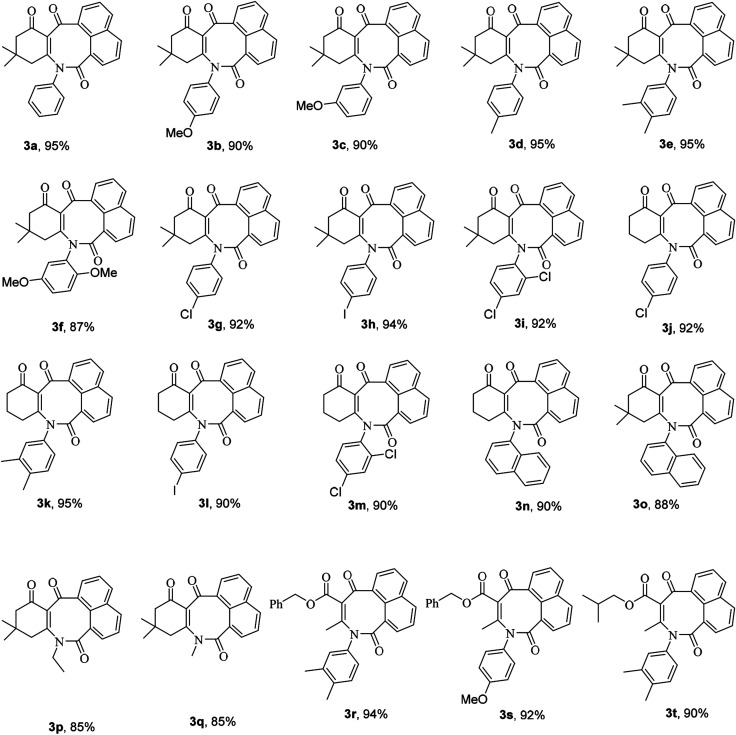

a Reaction conditions: enaminone (1 mmol), acenaphthoquinone (1 mmol); step 1: EtOH (4 mL), Et_3_N (1 mmol), reflux, 12 h; step 2: H_5_IO_6_ (1 mmol), r.t., 60 min.

**Fig. 2:**
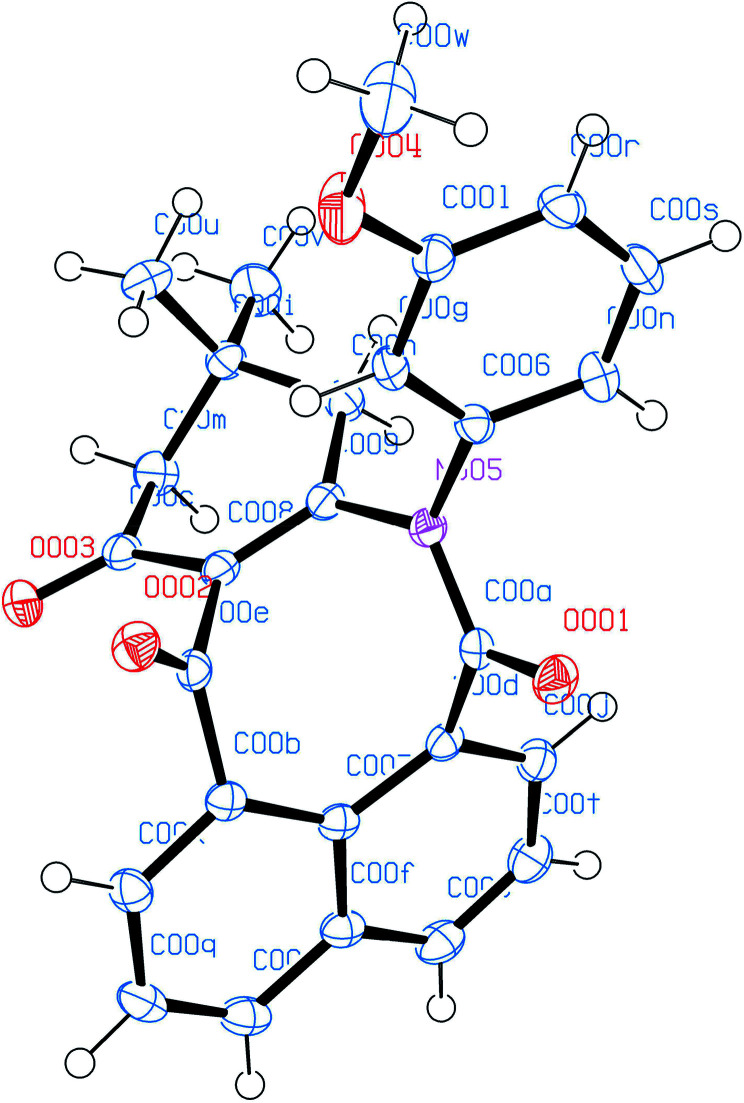
ORTED representation of 3c. CCDC 1976349.

The molecular structures of all products 3a–t were also elucidated from their IR, ^1^H-NMR, ^13^C-NMR and HRMS spectra. Finally, the structure of product 3c was determined unambiguously from an X-ray diffraction study (Fig. 2).

**Scheme 2:**
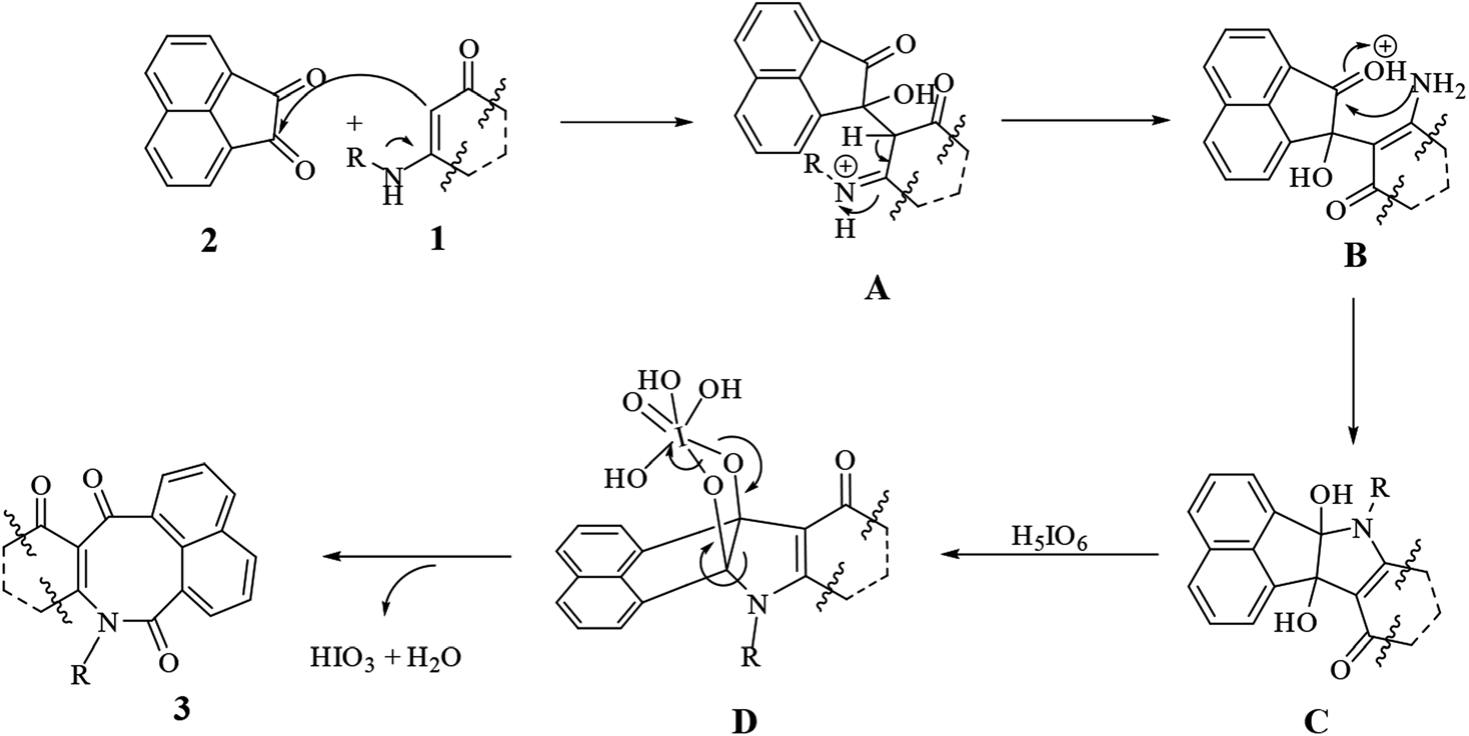
Proposed mechanism for the syntheses of compounds 3a–t

We proposed a general mechanism for the reactions, as shown in Scheme 2. According to the mechanism, first enaminone 1 attacked acenaphthoquinone 2 to form intermediate A, followed by a rearrangement of A to form intermediate B and then vicinal diol C. Then reacting C with periodic acid produced intermediate D, which then underwent a rearrangement to form product 3 with the loss of water and iodic acid molecules.

In summary, we have developed a novel pathway for the syntheses of new azocine derivatives *via* the reactions of enaminones with acenaphthoquinone followed by metal-free periodic acid-mediated oxidative cleavages of the corresponding vicinal diols. The novelty of the method in combination with high yields, short reaction times, and mild reaction conditions makes this procedure an especially attractive method for the syntheses of the titled compounds.

## Experimental

### General information

The chemicals used in this work were purchased from Merck and Sigma-Aldrich chemical companies and were used without purification. The progress of the reactions and the purity levels of the compounds were monitored using thin layer chromatography (TLC) analytical silica gel plates (Merck 60 F_250_). Melting points were determined using an Electro thermal 9100 apparatus. IR spectra were recorded using a Shimadzu IR-470 spectrometer with KBr plates. ^1^H-NMR and ^13^C-NMR spectra were recorded by using a Bruker DRX-400 AVANCE spectrometer in DMSO-d_6_ as solvent.

### General procedure for preparing enaminones 1a–t

In each case, 1,3-diketone (5 mmol), amine (5 mmol), I_2_ (0.1 mmol), and CH_3_CN (5 mL) were added to a reaction tube. The tube was then sealed and its contents stirred at room temperature for 1 h. In most cases, enaminone 1 precipitated from the reaction mixture as white crystals, which were collected on filter paper and further purified by washing them with cool acetonitrile (2 × 2 mL). When the product was soluble in acetonitrile, the solvent was removed under reduced pressure, with water (10 mL) then added and product extracted using ethyl acetate (3 × 3 mL). The organic layers were collected, washed with an aqueous Na_2_S_2_O_3_ solution and dried over anhydrous Na_2_SO_4_. After partial vaporization of solvent, the product was precipitated and the mixture was filtered to give the pure enaminone 1 as a white solid.

### General procedure for synthesizing azocine derivatives 3a–t

In each case, a mixture of enaminone 3 (1 mmol), Et_3_N (1 mmol), and acenaphthoquinone 2 (1 mmol) in EtOH (4 mL) was placed in a flask and the mixture was stirred for 12 hours at reflux conditions. The progress of the reaction was monitored by performing TLC using EtOAc/*n*-hexane as an eluent. After completion of the reaction, the reaction mixture was cooled to room temperature and H_5_IO_6_ (1 mmol) was added to the flask and the resulting mixture was stirred for an additional 1 hour. The reaction mixture was filtered and the crude product was recrystallized from ethanol to afford the pure product 3.

## Conflicts of interest

There are no conflicts to declare.

## Supplementary Material

RA-010-D0RA02852E-s001

RA-010-D0RA02852E-s002

## References

[cit1] (b) Brown J. D. , in Comprehensive Heterocyclic Chemistry, ed. A. R. Katrizky and C. W. Rees, Pergamon Press, Oxford, 1984, vol. 3, p. 57

[cit2] Arnold L. A., Kiplin Guy R. (2006). Bioorg. Med. Chem. Lett..

[cit3] Ma S., Gu Z. (2006). J. Am. Chem. Soc..

[cit4] Lehman P. G. (1972). Tetrahedron Lett..

[cit5] Grob C. A., Kunz W., Marbet P. R. (1975). Tetrahedron Lett..

[cit6] Leonard N. J., Sato T. (1969). J. Org. Chem..

[cit7] Garst M. E., Bonfiglio J. N., Marks J. (1982). J. Org. Chem..

[cit8] Nakatsubo F., Cocuzza A. J., Keeley D. E., Kishi Y. (1977). J. Am. Chem. Soc..

[cit9] Majumdar K. C., Chattopadhyay B., Samanta S. (2009). Tetrahedron Lett..

[cit10] Alcaide B., Almendros P., Aragoncillo C., Fernandez I., Gomez-Campillos G. (2014). J. Org. Chem..

[cit11] Penning M., Christoffers J. (2014). Eur. J. Org. Chem..

[cit12] Yet L. (2000). Chem. Rev..

[cit13] Soldatova S. A., Kolyadina N. M., Soldatenkov A. T., Malkova A. V. (2019). Russ. J. Org. Chem..

[cit14] Mohammadizadeh M. R., Alborz M. (2014). Tetrahedron Lett..

[cit15] Mohammadizadeh M. R., Firoozi N. (2010). Tetrahedron Lett..

[cit16] Datta B., Reddy M. B. M., Pasha M. A. (2011). Synth. Commun..

