# Supplementary material for: Metal-free syntheses of new azocines via addition reactions of enaminones with acenaphthoquinone followed by oxidative cleavages of the corresponding vicinal diols
Source: RSC Adv. 2020 May 29;10(35):20552–7. doi: 10.1039/d0ra02852e (PMC9054298; doi:10.1039/d0ra02852e)
Supplement: RA-010-D0RA02852E-s001 [file RA-010-D0RA02852E-s001.pdf]

Electronic Supplementary Material (ESI) for RSC Advances.
This journal is © The Royal Society of Chemistry 2020

Supporting information

Metal-free synthesis of new azocines via the addition reaction of enaminones with acenaphthoquinone followed by oxidative-cleavage of the corresponding vicinal diols

S. Hekmat Mousavi[a], Mohammad Reza Mohammadizadeh[*,a], Satoru Arimitsu[b], Dariush Saberi[c], Samira Poorsadeghi[b], Kojya Genta[d]

[a]Department of Chemistry, Faculty of Sciences, Persian Gulf University, Bushehr 75169, Iran, Fax: +98-771-4541494, E-mail: mrmohamadizadeh@pgu.ac.ir

[b]Department of Chemistry, Biology and Marine Science, Faculty of Science, University of the Ryukyus, 1-Senbaru, Nishihara, Nakagami, Okinawa, Japan, Zip code: 903-0213

[c]Marine Chemistry Department, Faculty of Marine Science and Technology, Persian Gulf University, Bushehr 75169, Iran

[d]Center for Research Advancement and Collaboration, University of the Ryukyus, Senbaru 1, Nishihara, Okinawa 903-0213, Japan

Table of Contents

	Page
General information	2
General procedure for the preparation of enaminones **1a-t**	2
General procedure for the synthesis of azocine derivatines **3a-t**	2
Spectrum information of the synthesized azocine derivatives	3
^{1}H-NMR and ^{13}C-NMR spectrum of the synthesized compounds	16

General information: The chemicals used in this work were purchased from Merck and Sigma-Aldrich chemical companies and were used without purification. The progress of the reaction and the purity of compounds were monitored by TLC analytical silica gel plates (Merck 60 F_{250}). Melting points were determined on an Electro thermal 9100 apparatus. IR spectra were recorded using a Shimadzu IR-470 spectrometer with KBr plates. ^{1}H-NMR and ^{13}C-NMR spectra were recorded on a Bruker DRX-400 AVANCE spectrometer in DMSO-d_6 as solvent.

General procedure for the preparation of enaminones 1a-t

1,3-Diketone (5 mmol), amine (5 mmol), I_2 (0.1 mmol), and CH_3CN (5 mL) were added to a reaction tube. The tube was then sealed and stirred at room temperature for 1 h. In the most cases, enaminones **1** were precipitate from the reaction mixture as white crystals, which were collected on filter paper and further purified by washing with cool acetonitrile (2×2 mL). When the products were soluble in acetonitrile, the solvent was removed under reduced pressure, then water (10 mL) added and products extracted by ethyl acetate (3×3 mL). The organic layers were collected, washed with aqueous $Na_2S_2O_3$ solution and dried over anhydrous Na_2SO_4. After partial vaporization of solvent, the products were precipitated and the mixture was filtered to give the pure enaminones **1** as white solids.

General procedure for the synthesis of azocine derivatives 3a-t:

A mixture of enaminone **3** (1 mmol), Et_3N (1 mmol), and acenaphthoquinone **2** (1 mmol) in EtOH (4 mL) was placed in the flask and the mixture stirred 12 hours at reflux condition. The progress of the reaction was monitored by thin layer chromatography using EtOAc/*n*-hexane as eluent. After completion of the reaction, the reaction mixture was cooled to room temperature and H_5IO_6 (1 mmol) was added to the flask and the mixture stirred for additional 1 hour. The reaction mixture was filtered and the crude product was recrystallized from ethanol to afford the pure product **3**.

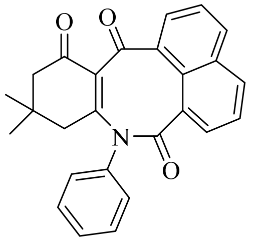

10,10-Dimethyl-8-phenyl-10,11-dihydro-7*H*-benzo[*b*]naphtho[1,8-*ef*]azocine 7,12,13(8*H*,9*H*)-trione (**3a**): isolated yield = 95%; white powder; mp = 224 °C; IR cm^{-1}: 1306, 1358, 1666, 1681, 1692, 1712, 2957, 3304; ^{1}H NMR (400 MHz, DMSO-d_6) δ 0.60 (s, 3H, CH_3), 0.84 (s, 3H, CH_3), 2.03 (d, J = 16.5 Hz, 1H, H-CH), 2.10-2.25 (m, 2H, CH_2), 2.33 (d, J = 17.7 Hz, 1H, H-CH), 7.40–7.55 (m, 3H, arom), 7.60 (t, J = 7.8 Hz, 2H, arom), 7.66–7.82 (m, 3H, arom), 7.94 (d, J = 7.1 Hz, 1H, arom), 8.19 (t, J = 8.1 Hz, 2H, arom); ^{13}C NMR (100 MHz, DMSO-d_6) δ 27.1, 27.4, 33.3, 50.3, 126.7, 127.2, 127.3, 127.6, 127.7, 128.7, 129.1, 130.1, 130.6, 132.4, 132.8, 134.7, 136.5, 137.0, 137.8, 151.6, 168.0, 196.1, 198.2. HRMS (ESI) calcd for $C_{26}H_{21}NO_3$ $[M+H]^+$: 396.1600; Found: 396.1595.

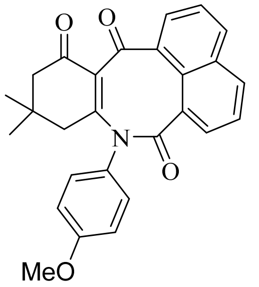

8-(4-Methoxyphenyl)-10,10-dimethyl-10,11-dihydro-7*H*-benzo[*b*]naphtho[1,8-*ef*]azocine-7,12,13(8*H*,9*H*)-trione (**3b**): isolated yield = 90%; white powder; mp >230 °C; IR cm^{-1}: 767, 810, 1356, 1639, 1671, 1693, 2923; ^{1}H NMR (400 MHz, DMSO-d_6) δ 0.62 (s, 3H, CH_3), 0.84 (s, 3H, CH_3), 2.02 (d, J = 16.5 Hz, 1H, H-CH), 2.12-2.24 (m, 2H, CH_2), 2.33 (d, J = 17.6 Hz, 1H, H-CH), 3.83 (s, 3H, OCH_3), 7.14 (d, J = 8.5 Hz, 2H, arom), 7.41 (d, J = 8.5 Hz, 2H, arom), 7.57–7.80 (m, 3H, arom), 7.91 (d, J = 6.9 Hz, 1 H, arom), 8.18 (t, J = 8.6 Hz, 2H, arom); ^{13}C NMR (100 MHz, DMSO-d_6) δ 27.0, 27.4, 33.3, 50.3, 55.9, 115.3, 126.6, 127.2, 127.3, 127.6, 128.7, 128.9, 130.2, 130.6, 132.4, 132.8, 134.8,

136.0, 137.0, 151.8, 159.6, 168.1, 196.1, 198.4. HRMS (ESI) calcd for $C_{27}H_{23}NO_4$ $[M+H]^+$: 426.1705; found: 426.1700.

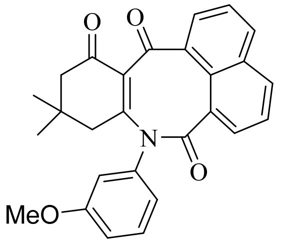

8-(3-Methoxyphenyl)-10,10-dimethyl-10,11-dihydro-7*H*-benzo[*b*]naphtho[1,8-*ef*]azocine-7,12,13(8*H*,9*H*)-trione (**3c**): isolated yield = 90%; white powder; mp >230 °C; IR cm^{-1}: 766, 826, 1638, 1654, 1685, 2967, 3296; ^{1}H NMR (400 MHz, DMSO-d_6) δ 0.61 (s, 3H, CH_3), 0.85 (s, 3H, CH_3), 2.03 (d, J = 16.5 Hz, 1H, H-CH), 2.15-2.26 (m, 2H, CH_2), 2.35 (d, J = 17.6 Hz, 1H, CH), 3.84 (s, 3H, OCH_3), 7.00-7.14 (m, 3H, arom), 7.50 (t, J = 7.9 Hz, 1H, arom), 7.67–7.80 (m, 3H, arom), 7.96 (d, J = 6.9 Hz, 1 H, arom), 8.18 (t, J = 8.4 Hz, 2H, arom); ^{13}C NMR (100 MHz, DMSO-d_6) δ 26.9, 27.5, 33.3, 50.3, 55.9, 113.7, 114.5, 119.7, 126.6, 127.2, 127.3, 127.7, 128.7, 130.6, 130.9, 132.4, 132.8, 134.7, 136.4, 137.0, 138.8, 151.6, 160.5, 167.9, 196.1, 198.2. HRMS (ESI) calcd for $C_{27}H_{23}NO_4$ $[M+H]^+$: 426.1705; found: 426.1699.

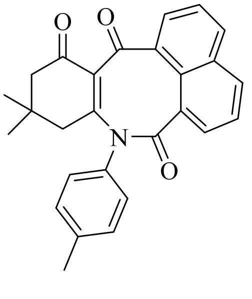

10,10-Dimethyl-8-(*p*-tolyl)-10,11-dihydro-7*H*-benzo[*b*]naphtho[1,8-*ef*]azocine-7,12,13(8*H*,9*H*)-trione (**3d**): isolated yield = 95%; white powder; mp = 225-227 °C; IR cm^{-1}: 765, 808, 1302, 1357, 1653, 2341,

2360, 3030; ^{1}H NMR (400 MHz, DMSO-d_6) δ 0.61 (s, 3H, CH_3), 0.84 (s, 3H, CH_3), 2.02 (d, J = 16.5 Hz, 1H, H-CH), 2.11-2.24 (m, 2H, CH_2), 2.32 (d, J = 17.7 Hz, 1H, H-CH), 2.38 (s, 3H, CH_3), 7.33-7.42 (m, 4H, arom), 7.66-7.77 (m, 3H, arom), 7.92 (d, J = 7.0 Hz, 1H, arom), 8.18 (t, J = 8.4 Hz, 2H, arom); ^{13}C NMR (100 MHz, DMSO-d_6) δ 21.2, 27.0, 27.4, 33.3, 50.3, 126.6, 127.2, 127.3, 127.4, 127.7, 128.6, 130.5, 130.6, 132.4, 132.8, 134.8, 135.2, 136.2, 137.0, 138.7, 151.7, 168.0, 196.0, 198.3. HRMS (ESI) calcd for $C_{27}H_{23}NO_3$ $[M+H]^+$: 410.1756; found: 410.1751.

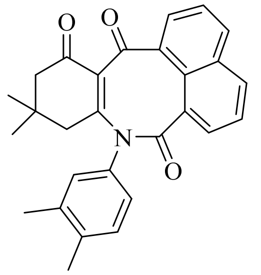

8-(3,4-Dimethylphenyl)-10,10-dimethyl-10,11-dihydro-7*H*-benzo[*b*]naphtho[1,8-*ef*]azocine-7,12,13(8*H*,9*H*)-trione (**3e**): isolated yield = 95%; white powder; mp = 227-229 °C; IR cm^{-1}: 773, 833, 1302, 1356, 1627, 1660, 1677, 2955; ^{1}H NMR (400 MHz, DMSO-d_6) δ 0.60 (s, 3H, CH_3), 0.84 (s, 3H, CH_3), 2.02 (d, J = 16.5 Hz, 1H, H-CH), 2.12-2.24 (m, 2H, CH_2), 2.29 (s, 3H, CH_3), 2.31 (s, 3H, CH_3), 2-27-2.32 (d overlapped with CH_3 singnals, 1H, H-CH), 7.18 (dd, J_1 = 7.9 Hz, J_2 = 1.6 Hz, 1H, Aarom), 7.28 (s, 1H, arom), 7.34 (d, J = 8.0 Hz, 1H, arom) 7.66-7.76 (m, 3H, arom), 7.92 (d, J = 6.8 Hz, 1H, arom), 8.18 (t, J = 8.5 Hz, 2H, arom); ^{13}C NMR (100 MHz, DMSO-d_6) δ 19.6, 19.9, 27.0, 27.4, 33.3, 50.3, 124.7, 126.6, 127.2, 127.4, 127.6, 128.4, 128.5, 130.5, 130.9, 132.4, 132.8, 134.9, 135.4, 136.1, 137.1, 137.4, 138.2, 151.7, 168.0, 196.1, 198.3. HRMS (ESI) calcd for $C_{28}H_{25}NO_3$ $[M+H]^+$: 424.1913; found: 424.1904.

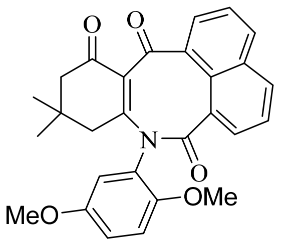

8-(2,5-Dimethoxyphenyl)-10,10-dimethyl-10,11-dihydro-7*H*-benzo[*b*]naphtho[1,8-*ef*]azocine-7,12,13(8*H*,9*H*)-trione (**3f**): isolated yield = 87%; white powder; mp >230 °C; IR cm^{-1}: 777, 1042, 1277, 1306, 1359, 1508, 1637, 1665, 1689, 2957; ^{1}H NMR (400 MHz, DMSO-d_6) δ 0.64 (s, 3H, CH_3), 0.85 (s, 3H, CH_3), 2.04 (d, J = 16.5 Hz, 1H, H-CH), 2.11-2.19 (m, 2H, CH_2), 2.23 (d, J = 17.6 Hz, 1H, H-CH), 3.78 (s, 3H, OCH_3), 3.92 (s, 3H, OCH_3), 6.8 (broad peak, 1H, arom), 7.08 (dd, J_1 = 9.0 Hz, J_2 = 3.1 Hz, 1H, arom), 7.22 (d, J = 9.0 Hz, 1H, arom) 7.68-7.79 (m, 4H, arom), 8.11-8.25 (m, 2H, arom); ^{13}C NMR (100 MHz, DMSO-d_6) δ 26.8, 27.6, 33.2, 50.3, 56.2, 57.0, 114.3, 115.2, 119.6, 126.7, 126.9, 127.3, 127.4, 127.5, 128.0, 130.4, 132.3, 132.5, 132.9, 135.0, 135.9, 137.3, 149.7, 151.8, 153.8, 167.5, 195.9. HRMS (ESI) calcd for $C_{28}H_{25}NO_5$ $[M+H]^+$: 456.1811; found: 456.1801.

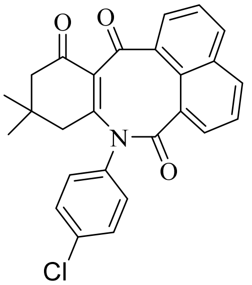

8-(4-Chlorophenyl)-10,10-dimethyl-10,11-dihydro-7*H*-benzo[*b*]naphtho[1,8-*ef*]azocine-7,12,13(8*H*,9*H*)-trione (**3g**): isolated yield = 92%; white powder; mp = 225-227 °C; IR cm^{-1}: 808, 1303, 1670, 2342, 2360, 2954; ^{1}H NMR (400 MHz, DMSO-d_6) δ 0.60 (s, 3H, CH_3), 0.85 (s, 3H, CH_3), 2.03 (d, J = 16.4 Hz, 1H, H-CH), 2.17-2.25 (m, 2H, CH_2), 2.34 (d, J = 17.7 Hz, 1H, H-CH), 7.53 (d, J = 8.5 Hz, 2H, arom), 7.67-7.73 (m, 3H, arom), 7.76 (d, J = 7.3 Hz, 2H, arom), 7.96 (d, J = 7.3 Hz, 1H, arom), 8.19 (t, J = 7.7 Hz, 2H, arom); ^{13}C NMR (100 MHz, DMSO-d_6) δ 27.1, 27.3, 33.3, 50.2, 126.7, 127.2,

127.3, 127.8, 128.8, 129.4, 130.2, 130.8, 132.4, 132.9, 134.4, 136.6, 136.7, 136.8, 151.2, 168.0, 196.1, 198.1. HRMS (ESI) calcd for $C_{26}H_{20}ClNO_3$ $[M+H]^+$: 430.1210; found: 430.1202.

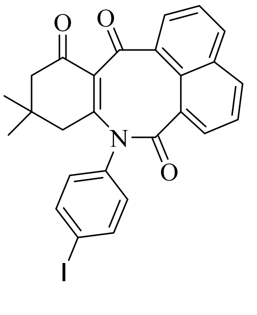

8-(4-Iodophenyl)-10,10-dimethyl-10,11-dihydro-7*H*-benzo[b]naphtho[1,8-*ef*]azocine-7,12,13(8*H*,9*H*)-trione (**3h**): isolated yield = 94%; white powder; mp >230 °C; IR cm^{-1}: 764, 1646, 2341, 2359, 2955; ^1H NMR (400 MHz, DMSO-d_6) δ 0.60 (s, 3H, CH_3), 0.86 (s, 3H, CH_3), 2.03 (d, *J* = 16.3 Hz, 1H, H-CH), 2.16-2.26 (m, 2H, CH_2), 2.32 (d, *J* = 17.5 Hz, 1H, H-CH), 7.31 (d, *J* = 8.2 Hz, 2H, arom), 7.68-7.73 (m, 1H, arom), 7.76 (d, *J* = 7.2 Hz, 2H, arom), 7.94-7.98 (m, 3H, arom), 8.19 (t, *J* = 7.9 Hz, 2H, arom); ^{13}C NMR (100 MHz, DMSO-d_6) δ 27.1, 27.3, 33.3, 50.3, 95.1, 126.7, 127.2, 127.3, 127.8, 128.8, 129.7, 130.7, 132.4, 132.9, 134.5, 136.8, 136.9, 137.5, 138.9, 151.1, 167.9, 196.0, 198.1. HRMS (ESI) calcd for $C_{26}H_{20}NO_3I$ $[M+H]^+$: 522.0566; found: 522.0554.

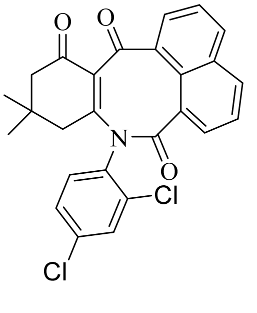

8-(2,4-Dichlorophenyl)-10,10-dimethyl-10,11-dihydro-7*H*-benzo[*b*]naphtho[1,8-*ef*]azocine-7,12,13(8*H*,9*H*)-trione (**3i**): isolated yield = 92%; white powder; mp >230 °C; IR cm^{-1}: 769, 817, 1306, 1360, 1634, 1671, 2360, 2951; ^1H NMR (400 MHz, DMSO-d_6) δ 0.69 (s, 3H, CH_3), 0.82 (s, 3H, CH_3), 2.08-2.15 (m, 3H, CH_2+H-CH), 2.36 (d, *J* = 17.7 Hz, 1H, H-CH), 7.39 (d, *J* = 8.4 Hz, 1H, arom), 7.70-

7.78 (m, 4H, arom) 7.85 (d, *J* = 6.7 Hz, 1H, arom), 8.00 (s, 1H, arom), 8.21 (d, *J* = 7.8 Hz, 2H, arom); ^{13}C NMR (100 MHz, DMSO-d_6) δ 26.3, 27.8, 30.1, 50.2, 126.8, 127.3, 127.4, 127.6, 128.4, 129.7, 130.2, 130.8, 131.0, 132.4, 133.9, 134.1, 134.2, 135.1, 136.1, 136.8, 150.7, 167.1, 196.0, 198.6. HRMS (ESI) calcd for $C_{26}H_{19}NO_3Cl_2$ $[M+H]^+$: 464.0820; found: 464.0808.

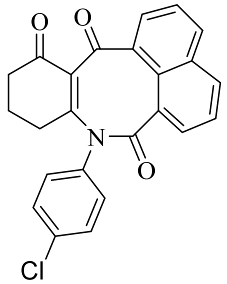

8-(4-Chlorophenyl)-10,11-dihydro-7*H*-benzo[*b*]naphtho[1,8-*ef*]azocine-7,12,13(8*H*,9*H*)-trione (**3j**): isolated yield = 92%; white powder; mp = 229 °C; IR cm^{-1}: 777, 1291, 1361, 1637, 1647, 1685, 1707, 1718, 2345, 2917; ^{1}H NMR (400 MHz, DMSO-d_6) δ 1.68-1.82 (m, 2H, CH_2), 1.96-2.19 (m, 2H, CH_2), 2.20-2.32 (m, 2H, CH_2), 7.51-7.59 (m, 2H, arom), 7.64-7.80 (m, 5H, arom), 7.98 (dd, J_1 = 7.8 Hz, J_2 = 0.8 Hz, 1H, arom), 8.14-8.24 (m, 2H, arom); ^{13}C NMR (100 MHz, DMSO-d_6) δ 20.9, 27.0, 36.9, 126.6, 127.2, 127.3, 127.8, 128.8, 129.3, 130.2, 130.7, 132.4, 132.8, 133.4, 134.3, 136.9, 137.0, 137.7, 153.3, 168.0, 196.0, 198.0. HRMS (ESI) calcd for $C_{24}H_{18}NO_3$ $[M+H]^+$: 402.0897; found: 402.0891.

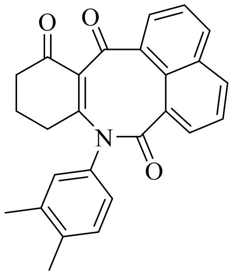

8-(3,4-Dimethylphenyl)-10,11-dihydro-7*H*-benzo[*b*]naphtho[1,8-*ef*]azocine-7,12,13(8*H*,9*H*)-trione (**3k**): isolated yield = 95%; white powder; mp = 225-227 °C; IR cm^{-1}: 779, 1295, 1361, 1637, 1653, 1693, 2341, 2360, 2962; ^{1}H NMR (400 MHz, DMSO-d_6) δ 1.64-1.81 (m, 2H, CH_2), 2.03-2.15 (m, 1H,

CH), 2.15-2.28 (m, 2H, CH_2), 2.29 (s, 3H, CH_3), 2.31 (s, 3H, CH_3), 2.38-2.45 (m, 1H, CH), 7.20 (dd, J_1 = 7.5 Hz, J_2 = 2.2 Hz, 1H, arom), 7.29 (s, 1H, arom), 7.3 (d, J = 7.8 Hz, 1H, arom), 7.64-7.80 (m, 3H, arom), 7.92 (d, J = 6.7 Hz, 1H, arom), 8.18 (t, J = 7.6 Hz, arom); ^{13}C NMR (100 MHz, DMSO-d_6) δ 19.6, 19.9, 20.9, 27.1, 37.0, 124.6, 126.6, 127.2, 127.3, 127.7, 128.2, 128.5, 130.5, 131.0, 132.4, 132.7, 134.8, 135.8, 137.0, 137.1, 137.4, 138.3, 153.9, 168.1, 196.0, 198.5. HRMS (ESI) calcd for $C_{26}H_{21}NO_3$ $[M+H]^+$: 396.1600; found: 396.1592.

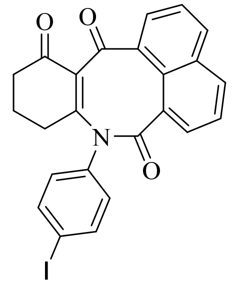

8-(4-Iodophenyl)-10,11-dihydro-7*H*-benzo[*b*]naphtho[1,8-*ef*]azocine-7,12,13(8*H*,9*H*)-trione (**3l**): isolated yield = 90%; white powder; mp >230 °C; IR cm^{-1}: 797, 1004, 1651, 2341, 2359, 2871, 3052; ^{1}H NMR (400 MHz, DMSO-d_6) δ 1.66-1.85 (m, 2H, CH_2), 2.04-2.16 (m, 1H, H-CH), 2.18-2.33 (m, 2H, CH_2), 2.39-2.47 (m, 1H, H-CH), 7.33 (d, J = 8.2 Hz, 2H, arom), 7.60-7.80 (m, 3H, arom), 7.90-7.80 (m, 3H, arom), 7.86-8.01 (m, 3H, arom), 8.19 (t, J = 6.8 Hz, 2H, arom); ^{13}C NMR (100 MHz, DMSO-d_6) δ 20.8, 27.0, 36.9, 95.0, 126.6, 127.2, 127.3, 127.8, 128.7, 129.6, 130.7, 132.4, 132.8, 134.3, 136.9, 137.7, 137.9, 139.0, 153.2, 167.9, 196.0, 198.3. HRMS (ESI) calcd for $C_{24}H_{16}NO_3I$ $[M+H]^+$: 494.253; found: 494.0242.

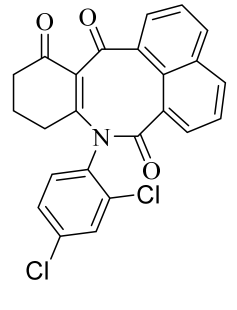

8-(2,4-Dichlorophenyl)-10,11-dihydro-7*H*-benzo[*b*]naphtho[1,8-*ef*]azocine-7,12,13(8*H*,9*H*)-trione (**3m**): isolated yield = 90%; white powder; mp >230 °C; IR cm-1: 769, 811, 1127, 1644, 2349, 2359, 2889, 3089; ^{1}H NMR (400 MHz, DMSO-d_6) δ 1.60-1.86 (m, 2H, CH_2), 2.06-2.29 (m, 3H, CH_2), 2.41-2.49 (m, 1H, H-CH), 7.42 (d, J = 8.3 Hz, 1H, arom), 7.65-7.80 (m, 3H, arom), 7.78 (d, J = 7.2 Hz, 2H, arom),7.85 (d, J = 6.8 Hz, 1H, arom), 8.00 (brs, 1H, arom), 8.21 (d, J = 8.0 Hz, 2H, arom); ^{13}C NMR (100 MHz, DMSO-d_6) δ 20.6, 26.3, 36.9, 126.7, 127.3, 127.4, 127.7, 128.5, 129.8, 130.3, 130.7, 131.0, 132.4, 132.8, 133.8, 134.1, 134.7, 135.1, 136.8, 136.9, 152.9, 167.2, 196.02, 198.8. HRMS (ESI) calcd for $C_{24}H_{15}NO_3Cl_2$ $[M+H]^+$: 436.0507; found: 436.0497.

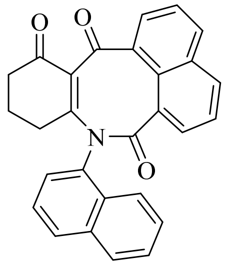

8-(Naphthalen-1-yl)-10,11-dihydro-7*H*-benzo[*b*]naphtho[1,8-*ef*]azocine-7,12,13(8*H*,9*H*)-trione (**3n**): isolated yield = 90%; white powder; mp >230 °C; IR cm^{-1}: 793, 1015, 1191, 1295, 1363, 1610, 1634, 1661, 1690, 2358, 2873, 3050; The ^{1}H NMR (400 MHz, DMSO-d_6) spectra of this compound shows the existence of two of diastereomers (conformers) which their ratio was calculated from the integrals of the relative peaks as 1:0.21; δ 1.45-1.82 (m, 2.4H, CH_2 of major and minor conformers), 1.89-2.08 (m, 1.2H, H-CH of both conformers), 2.10-2.31 (m, 2.42H, CH_2 of both conformers), 2.54-2.64 (m, 1.21H, H-CH of both conformers), 7.58 (d, J = 6.9 Hz, 1H, arom), 7.63-7.95 (m, 7.26H, arom), 7.97-8.33 (m, 7.47H, arom); ^{13}C NMR (100 MHz, DMSO-d_6) δ 20.5, 20.8, 26.7, 27.0, 36.9, 122.4, 122.7, 125.5, 126.6, 126.6, 126.7, 126.8, 127.2, 127.3, 127.4, 127.5, 127.8, 127.9, 128.1, 128.4, 128.7, 128.9, 129.1, 129.2, 130.1, 130.2, 130.3, 130.6, 130.9, 131.7, 132.3, 132.5, 132.7, 132.8, 134.4, 134.5, 134.7, 134.9, 135.1, 135.2, 136.5, 137.0, 137.4, 154.2, 154.3, 168.1, 170.9, 196.2, 199.0. HRMS (ESI) calcd for $C_{28}H_{19}NO_3$ $[M+H]^+$: 418.1443; found: 418.1439.

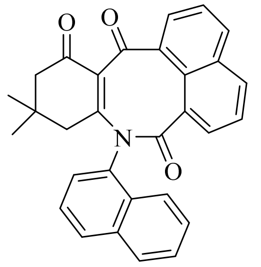

10,10-Dimethyl-8-(naphthalen-1-yl)-10,11-dihydro-7*H*-benzo[*b*]naphtho[1,8-*ef*]azocine-7,12,13(8*H*,9*H*)-trione (**3o**): isolated yield = 88%; white powder; mp >230 °C; IR cm^{-1}: 770, 805, 1313, 1357, 1680, 1633, 1666, 1680, 2360, 2952; The ^{1}H NMR (400 MHz, DMSO-d_6) spectra of this compound shows the existence of two of diastereomers (conformers) which their ratio was calculated from the integrals of the relative peaks as 1:0.18; δ 0.60 (s, 0.57H, CH_3 of minor conformer), 0.64 (s, 0.57H, CH_3 of minor conformer), 0.67 (s, 3H, CH_3 of major conformer), 0.74 (s, 3H, CH_3 of major conformer), 1.80-2.03 (m, 1.5H, H-CH of minor and major conformers), 2.03-2.20 (m, 2.38H, H-CH_2 of minor and major conformers), 3.31-2.49 (m, 1H, H-CH of major conformer), 7.57 (d, *J* = 7.2 Hz, 1H, arom), 7.65-7.77 (m, 3.55H, arom), 7.78-7.90 (m, 3.73H, arom), 8.06 (*J* = 6.9 Hz, 1H, arom) 8.13 (d, J= 9.3 Hz, 2H, aqrom), 8.02-8.16 (m, 0.54H, arom), 8.18 (d, *J* = 8.5 Hz, 1H, arom), 8.23 (d, *J* = 8.1 Hz, 2H, arom), 8.17-8.26 (m, 0.55H, arom); ^{13}C NMR (100 MHz, DMSO-d_6) δ 26.0, 27.0, 27.6, 28.0, 32.8, 32.9, 49.9, 50.3, 56.5, 122.7, 125.4, 125.5, 126.4, 126.8, 127.2, 127.4, 127.5, 127.7, 127.8, 128.5, 128.6, 128.8, 129.1, 129.3, 130.1, 130.3, 130.9, 132.5, 132.7, 132.8, 133.0, 134.4, 134.6, 134.7, 135.9, 137.0 151.9, 168.0, 196.1, 198.8. HRMS (ESI) calculated for $C_{30}H_{23}NO_3$ $[M+H]^+$: 446.1756; found: 446.1748.

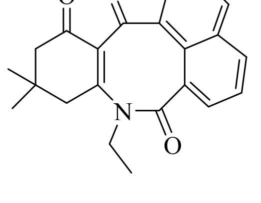

8-Ethyl-10,10-dimethyl-10,11-dihydro-7*H*-benzo[*b*]naphtho[1,8-*ef*]azocine-7,12,13(8*H*,9*H*)-trione (**3p**): isolated yield = 85%; white powder; mp = 226-228 °C; IR cm^{-1}: 669, 1284, 1634, 1689, 2341,

2359, 2959; ^{1}H NMR (400 MHz, DMSO-d_6) δ 0.57 (s, 3H, CH_3), 1.00 (s, 3H, CH_3), 1.18 (t, J = 6.9 Hz, 3H, CH_3), 1.96 (d, J = 16.5 Hz, 1H, H-CH), 2.21-2.32 (m, 2H, CH_2), 2.86 (d, J = 17.5 Hz, 1H, H-CH), 3.54 (m, 1H, H-CHEt), 4.06 (dq, J_1= 14.1 Hz, J_2= 7.1 Hz, 1H, H-CHEt), 7.59 (d, J = 6.9 Hz, 1H, arom), 7.63-7.70 (m, 3H, arom), 8.05-8.22 (m, 2H, arom); ^{13}C NMR (100 MHz, DMSO-d_6) δ 12.9, 26.9, 28.0, 33.6, 50.3, 126.5, 127.2, 127.3, 127.8, 128.6, 130.4, 132.3, 132.8, 135.1, 136.2, 137.0, 152.2, 167.1, 196.1, 197.7. 136.1, 136.8, 150.7, 167.1, 196.0, 198.6. HRMS (ESI) calcd for $C_{22}H_{21}NO_3$ $[M+H]^+$: 348.1600; found: 348.1593.

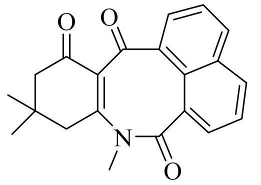

8,10,10-Trimethyl-10,11-dihydro-7*H*-benzo[*b*]naphtho[1,8-*ef*]azocine-7,12,13(8*H*,9*H*)-trione (**3q**): isolated yield = 85%; white powder; mp = 222-224 °C; IR cm^{-1}: 777, 1362, 1636, 1684, 2342, 2360, 2957; ^{1}H NMR (400 MHz, DMSO-d_6) δ 0.57 (s, 3H, CH_3), 1.00 (s, 3H, CH_3), 1.97 (d, J = 16.4 Hz, 1H, H-CH), 2.17-2.36 (m, 2H, CH_2), 2.88 (d, J = 17.5 Hz, 1H, H-CH), 3.35 (s, 3H, CH_3), 7.59-7.73 (m, 5H, arom), 8.13 (dd, J_1= 8.1 Hz, J2= 1.2 Hz, 1H, arom), 8.16 (dd, J_1= 7.8 Hz, J2= 1.8 Hz, 1H, arom); ^{13}C NMR (100 MHz, DMSO-d_6) δ 26.8, 28.0, 33.2, 33.6, 50.3, 126.4, 127.1, 127.3, 127.8, 129.1, 130.6, 132.4, 132.9, 134.8, 135.3, 137.1, 153.5, 167.6, 196.1, 197.6. HRMS (ESI) calcd for $C_{21}H_{19}NO_3$ $[M+H]^+$: 334.1443; found: 334.1439.

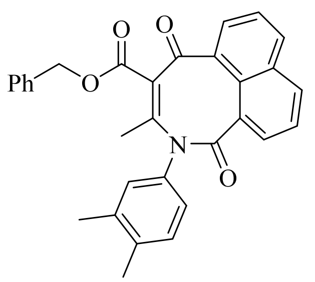

Benzyl (E)-2-(3,4-dimethylphenyl)-3-methyl-1,5-dioxo-2,5-dihydro-1H-naphtho[1,8-cd]azocine-4-carboxylate (**3r**): isolated yield = 94%; white powder; mp >230 °C; IR cm^{-1}: 778, 818, 1224, 1251, 1509, 1644, 1658, 1686, 1719, 2341, 2360, 2956, 3006; The ^{1}H NMR (400 MHz, DMSO-d_6) spectra of this compound shows the existence of two of diastereomers (conformers) which their ratio was calculated from the integrals of the relative peaks as 1:0.2; δ 1.91 (s, 0.59H, CH_3 of minor conformer), 2.12 (s, 3H, CH_3 of major conformer), 2.14 (s, 1.2H, 2*CH_3 of minor conformer), 2.28 (s, 3H, CH_3 of major conformer), 2.30 (s, 3H, CH_3 of major conformer), 4.92 (d, J = 12.4 Hz, 1H, H-CH of major conformer), 4.99 (d, J = 12.4 Hz, 1H, CH_2 of major conformer), 5.19 (s, 0.42H, CH_2 of minor conformer), 6.19 (dd, J_1= 8.0 Hz, J_2=2.0 Hz, 0.21H, arom), 6.28 (d, J= 2.0 Hz, 0.21H, arom), 6.93-7.08 (m, 2.12H, arom), 7.09-7.17 (m, 0.49H, arom), 7.18-7.45 (m, 6.65H, arom), 7.53-7.64 (m, 2H, arom), 7.74 (t, J = 8.0 Hz, 1H, arom), 7.84-8.02 (m, 1.59H, arom), 8.14-8.27 (m, 2H, arom), 8.37 (dd, J_1= 7.2 Hz, J_2=1.2 Hz, 0.53H, arom), 8.52 (dd, J_1= 8.4 Hz, J_2=1.2 Hz, 0.53H, arom); ^{13}C NMR (100 MHz, DMSO-d_6) δ 18.2, 18.7, 19.2, 19.6, 19.8, 19.9, 66.8, 67.7, 85.3, 116.4, 120.3, 124.8, 126.4, 127.3, 127.4, 127.5, 127.8, 127.9, 128.3, 128.4, 128.5, 128.5, 128.6, 128.6, 128.7, 128.8, 129.0, 130.4, 130.5, 130.7, 130.8, 131.1, 132.2, 132.4, 132.6, 133.0, 134.8, 135.4, 135.6, 135.7, 135.8, 136.2, 137.3, 137.4, 138.0, 147.3, 149.5, 161.9, 165.5, 166.2, 167.1, 189.8, 196.7. HRMS (ESI) calcd for $C_{31}H_{25}NO_4$ $[M+H]^+$: 476.1784; found: (No expected molecular ion was observed).

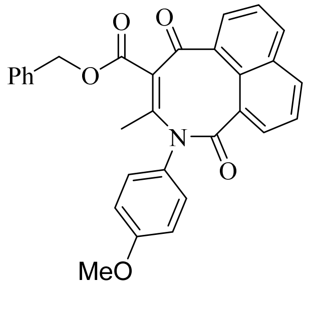

Benzyl(E)-2-(4-methoxyphenyl)-3-methyl-1,5-dioxo-2,5-dihydro-1H-naphtho[1,8-cd]azocine-4-carboxylate (**3s**): isolated yield = 92%; white powder; mp >230 °C; IR cm^{-1}: 778, 818, 1061, 1224, 1251, 1509, 1644, 1648, 1686, 1719, 2360, 2956, 3033; The ^{1}H NMR (400 MHz, DMSO-d_6) spectra of this compound shows the existence of two of diastereomers (conformers) which their ratio was

calculated from the integrals of the relative peaks as 1:0.2; δ 1.94 (s, 0.6H, CH_3 of minor conformer), 2.12 (s, 3H, CH_3 of major conformer), 3.70 (s, 0.6H, OCH_3 of minor conformer), 3.82 (s, 3H, CH_3, OCH_3 of major conformer), 4.93 (d, J = 12.4 Hz, 1H, CH_2 of major conformer), 5.00 (d, J = 12.4 Hz, 1H, CH_2 of major conformer), 5.19 (s, 0.4 H, CH_2 of minor conformer), 6.45 (dd, J_1= 6.6 Hz, J_2=2.0 Hz, 0.4H, arom), 6.86 (dd, J_1= 6.8 Hz, J_2=2.0 Hz, 0.4H, arom), 6.93-7.00 (m, 2H, arom), 7.08-7.18 (m, 2.42H, arom), 7.23-7.27 (m, 0.6H, arom), 7.28-7.36 (m, 3H, arom), 7.41-7.48 (m, 2.H, arom), 7.56 (d, J=7.6 Hz, 1H, arom), 7.59 (d, J= 7.2 Hz, 1H, arom), 74 (t, J= 7.2 Hz, 1H, arom), 7.86-7.92 (m+dd, J_1= 7.2 Hz, J_2= 0.8 Hz, 1.4H, arom), 8.14-8.24 (m, 2H, arom), 8.37 (dd, J_1= 7.2 Hz, J_2= 1.2 Hz, 0.4H, arom), 8.52 (dd, J_1= 8.0 Hz, J_2= 1.2 Hz, 0.4H, arom); ^{13}C NMR (100 MHz, DMSO-d_6) δ 18.1, 18.7, 55.6, 55.9, 66.8, 67.6, 114.7, 115.2, 120.9, 126.4, 127.3, 127.8, 127.4, 127.5, 127.8, 128.3, 128.4, 128.5, 128.6, 128.7, 128.8, 128.8, 128.9, 129.0, 130.5, 130.6, 131.0, 132.4, 132.6, 133.0, 134.7, 135.4, 135.6, 135.7, 142.3, 149.5, 156.5, 159.4, 161.9, 165.6, 166.4, 167.2, 189.8, 196.8. HRMS (ESI) calcd for $C_{30}H_{23}NO_5$ $[M+H]^+$: 478.1674; found: 478.1574.

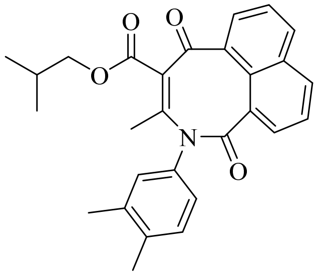

Isobutyl (E)-2-(3,4-dimethylphenyl)-3-methyl-1,5-dioxo-2,5-dihydro-1H-naphtho[1,8-cd]azocine-4-carboxylate (**3t**): isolated yield = 90%; white powder; mp >230 °C; IR cm^{-1}: 780, 1064, 1233, 1324, 1501, 1644, 1661, 1686, 1717, 2360, 2890, 2962; The ^1H NMR (400 MHz, DMSO-d_6) spectra of this compound shows the existence of two of diastereomers (conformers) which their ratio was calculated from the integrals of the relative peaks as 1:0.2; δ 0.60 (d, J = 6.8 Hz, 3H, CH_3 of major conformer), 0.66-0.80 (m+d, J = 6.8 Hz, 3.5H, CH_3 of major and minor conformers), 1.65 (two overlapped septets, J = 6.8 Hz, 1.11H, CH of major and minor conformers), 1.90 (s, 0.33H, CH_3 of minor conformer), 2.12 (s, 3H, CH_3 of major conformer), 2.14 (s, 0.6H, CH_3 of minor conformer), 2.28 (s, 3H, CH_3 of major

conformer), 2.30 (s, 3H, CH_3 of major conformer), 3.60-3.74 (m, 2H, CH_2 of major conformer), 3.88 (d, J= 6.0 Hz, CH_2 of minor conformer), 6.20 (d, J= 7.6 Hz, 0.11H, arom), 6.3 (s, 0.11H, arom), 7.03 (s, 0.11H, arom), 7.11-7.45 (m, 3H, arom), 7.68-7.93 (m, 4H, arom), 8.18 (d, J= 8.4 Hz, 1H, arom), 8.21 (d, J= 7.6 Hz, 1H, arom), 8.38 (d, J= 7.6 Hz, 0.33H, arom), 8.52 (d, J= 8.4 Hz, 0.33H, arom); ^{13}C NMR (100 MHz, DMSO-d_6) δ 19.1, 19.2, 19.4, 20.1, 24.4, 27.8, 28.0, 30.7, 71.6, 107.3, 117.0, 120.7, 126.8, 127.5, 129.6, 129.9, 131.1, 131.9, 135.1, 136.6, 137.5, 168.4, 170.9. HRMS (ESI) calcd for $C_{28}H_{27}NO_4$ $[M+H]^+$: 442.1940; found: (No expected molecular ion was observed).

HNMR Spectrum of Compound **3a**

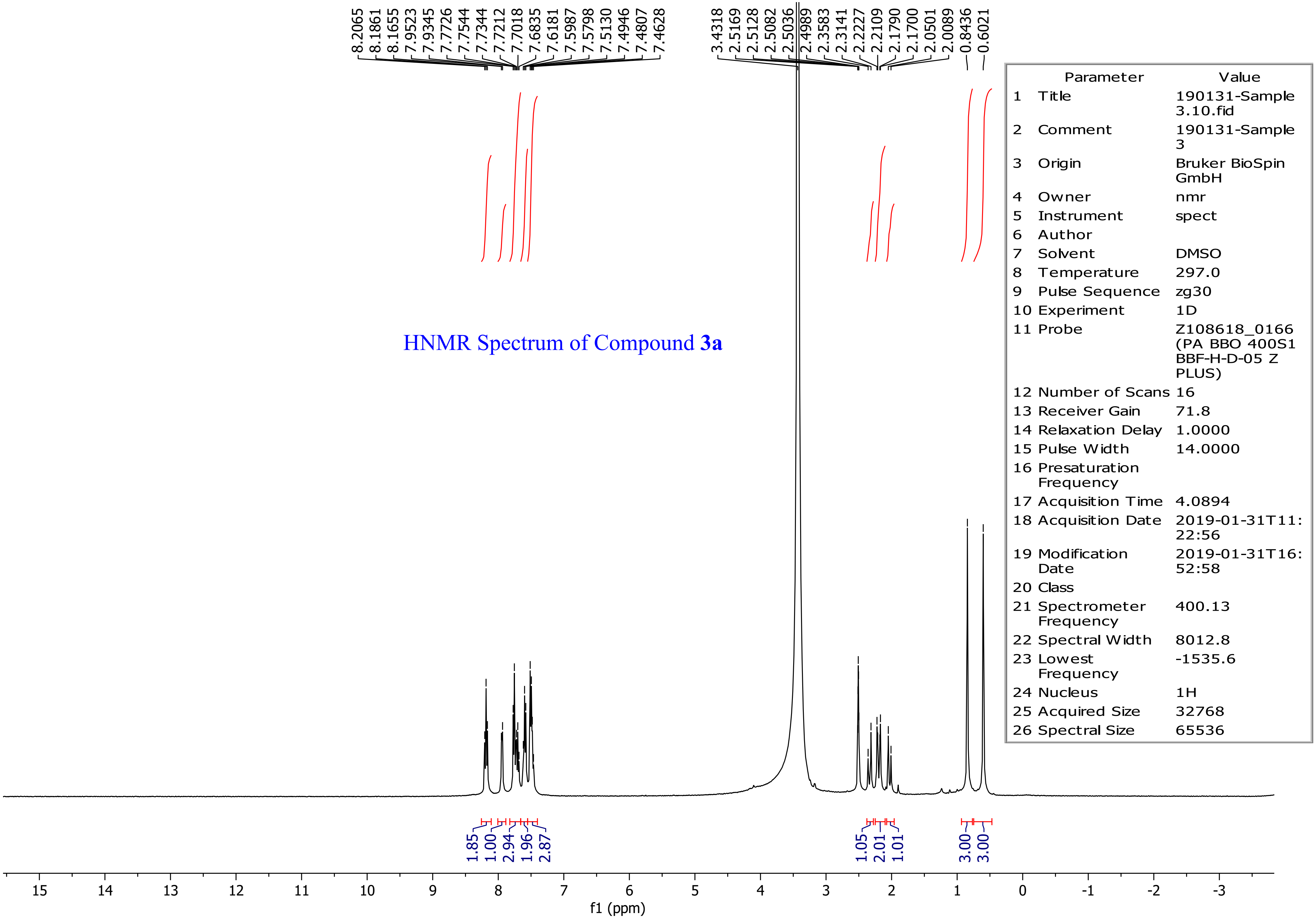

	Parameter	Value
1	Title	190131-Sample 3.10.fid
2	Comment	190131-Sample 3
3	Origin	Bruker BioSpin GmbH
4	Owner	nmr
5	Instrument	spect
6	Author	
7	Solvent	DMSO
8	Temperature	297.0
9	Pulse Sequence	zg30
10	Experiment	1D
11	Probe	Z108618_0166 (PA BBO 400S1 BBF-H-D-05 Z PLUS)
12	Number of Scans	16
13	Receiver Gain	71.8
14	Relaxation Delay	1.0000
15	Pulse Width	14.0000
16	Presaturation Frequency	
17	Acquisition Time	4.0894
18	Acquisition Date	2019-01-31T11:22:56
19	Modification Date	2019-01-31T16:52:58
20	Class	
21	Spectrometer Frequency	400.13
22	Spectral Width	8012.8
23	Lowest Frequency	-1535.6
24	Nucleus	1H
25	Acquired Size	32768
26	Spectral Size	65536

CNMR Spectrum of Compound **3a**

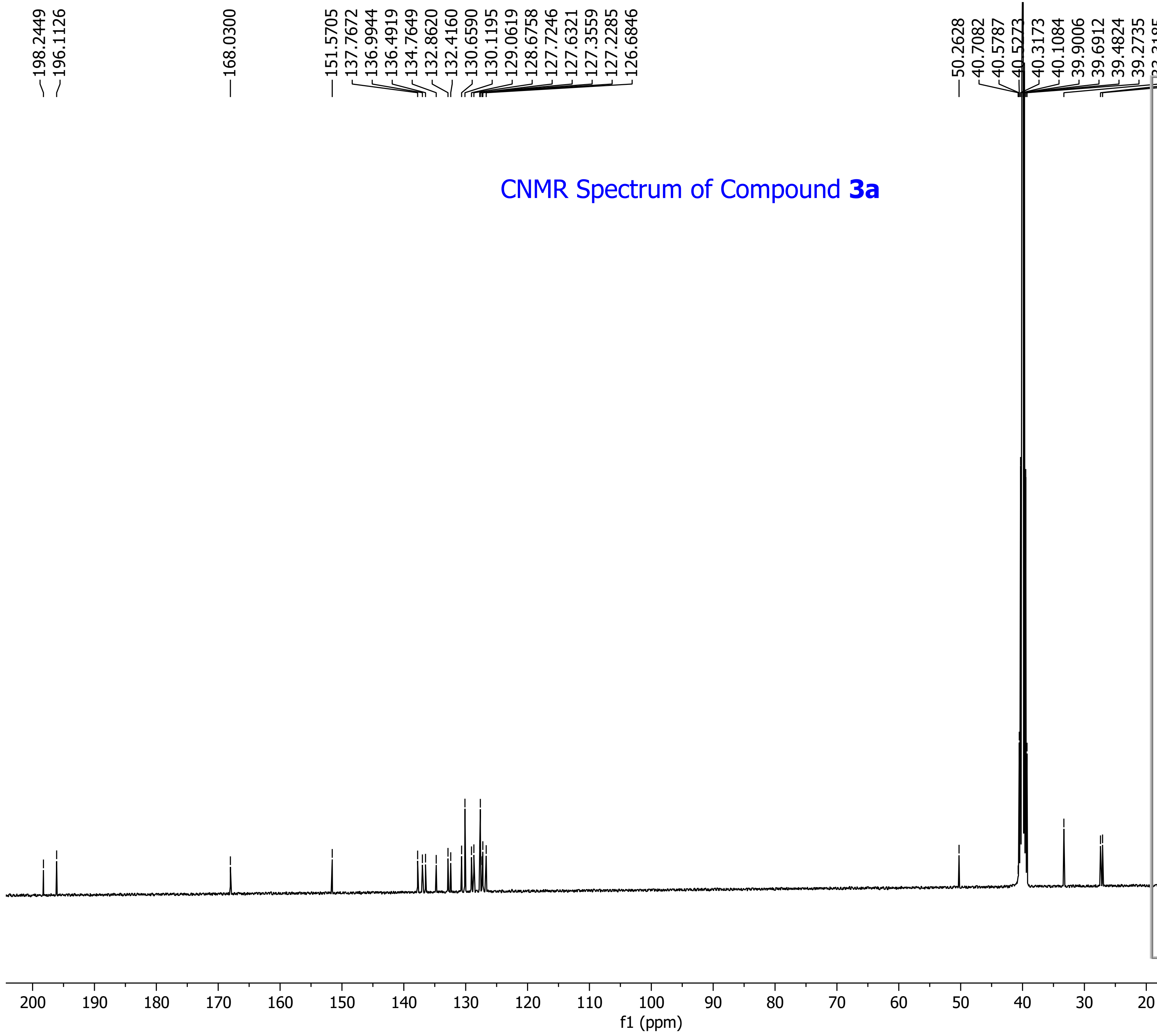

	Parameter	Value
1	Title	190208-Sample 3-CNMR.10.fid
2	Comment	190208-Sample 3-CNMR
3	Origin	Bruker BioSpin GmbH
4	Owner	nmr
5	Instrument	spect
6	Author	
7	Solvent	DMSO
8	Temperature	298.1
9	Pulse Sequence	zgpg30
10	Experiment	1D
11	Probe	Z108618_0166 (PA BBO 400S1 BBF-H-D-05 Z PLUS)
12	Number of Scans	4285
13	Receiver Gain	114.0
14	Relaxation Delay	2.0000
15	Pulse Width	9.0000
16	Presaturation Frequency	
17	Acquisition Time	1.3631
18	Acquisition Date	2019-02-08T16:29:39
19	Modification Date	2019-02-08T21:59:42
20	Class	
21	Spectrometer Frequency	100.62
22	Spectral Width	24038.5
23	Lowest Frequency	-1958.4
24	Nucleus	13C
25	Acquired Size	32768
26	Spectral Size	65536

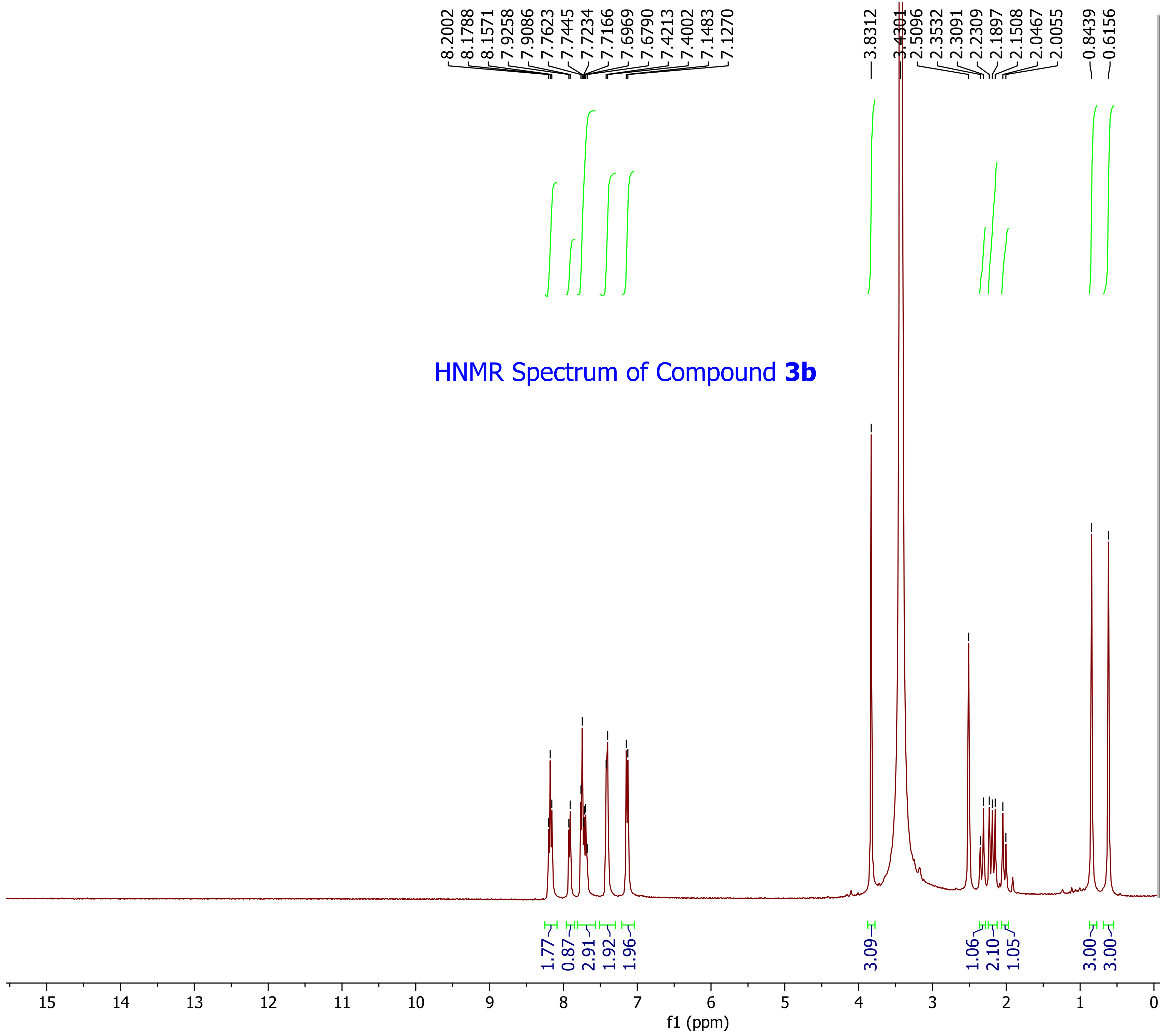

HNMR Spectrum of Compound **3b**

	Parameter	Value
1	Title	190128-Sample 2-HNMR.20.fid
2	Comment	190128-Sample 2-HNMR
3	Origin	Bruker BioSpin GmbH
4	Owner	nmr
5	Instrument	spect
6	Author	
7	Solvent	DMSO
8	Temperature	296.4
9	Pulse Sequence	zg30
10	Experiment	1D
11	Probe	Z108618_0166 (PA BBO 400S1 BBF-H-D-05 Z PLUS)
12	Number of Scans	16
13	Receiver Gain	80.6
14	Relaxation Delay	1.0000
15	Pulse Width	14.0000
16	Presaturation Frequency	
17	Acquisition Time	4.0894
18	Acquisition Date	2019-01-28T12:23:30
19	Modification Date	2019-01-28T17:53:32
20	Class	
21	Spectrometer Frequency	400.13
22	Spectral Width	8012.8
23	Lowest Frequency	-1535.6
24	Nucleus	1H
25	Acquired Size	32768
26	Spectral Size	65536

CNMR Spectrum of Compound **3b**

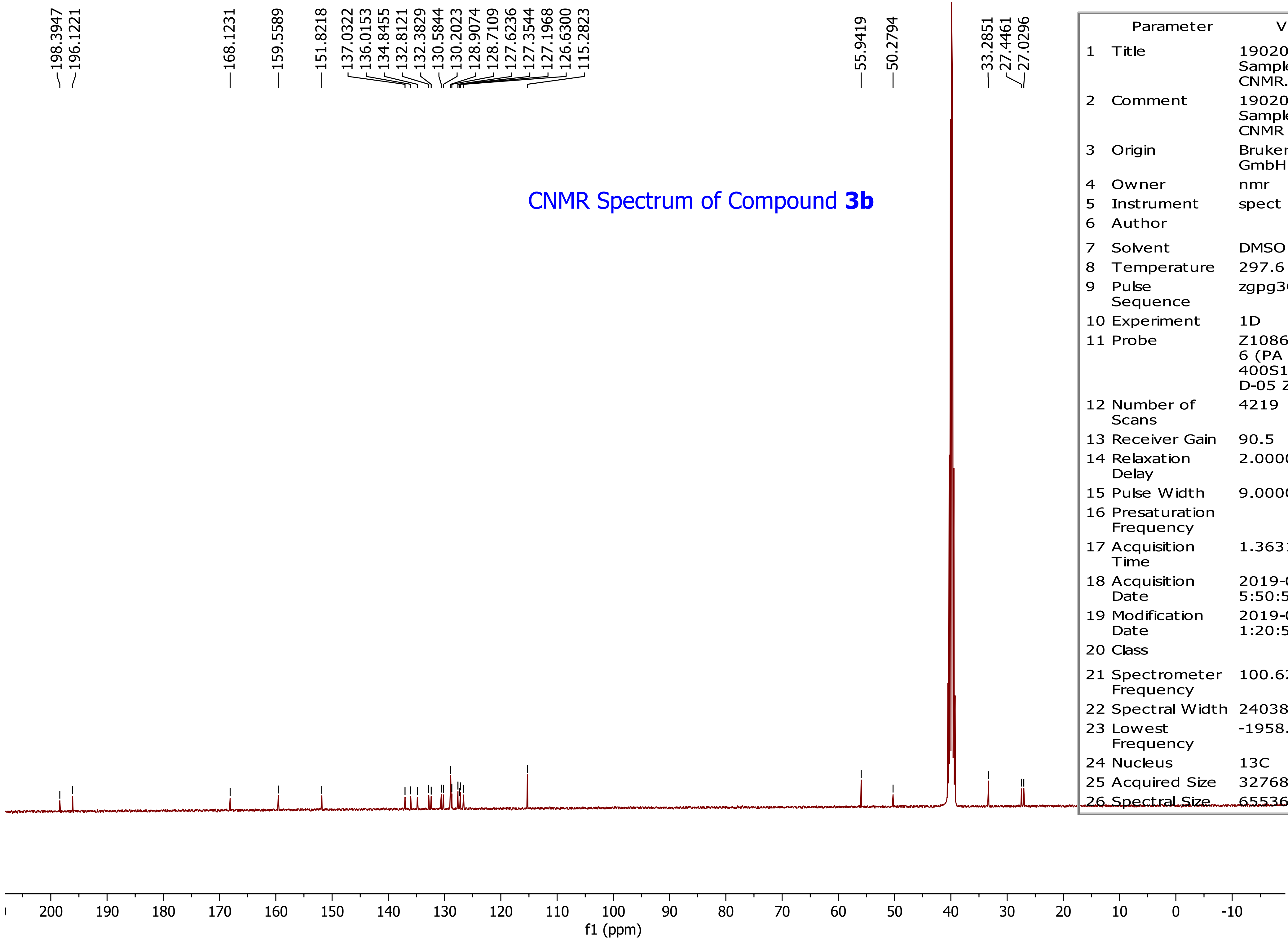

	Parameter	Value
1	Title	190203-Sample 2-CNMR.10.fid
2	Comment	190203-Sample 2-CNMR
3	Origin	Bruker BioSpin GmbH
4	Owner	nmr
5	Instrument	spect
6	Author	
7	Solvent	DMSO
8	Temperature	297.6
9	Pulse Sequence	zgpg30
10	Experiment	1D
11	Probe	Z108618_0166 (PA BBO 400S1 BBF-H-D-05 Z PLUS)
12	Number of Scans	4219
13	Receiver Gain	90.5
14	Relaxation Delay	2.0000
15	Pulse Width	9.0000
16	Presaturation Frequency	
17	Acquisition Time	1.3631
18	Acquisition Date	2019-02-03T15:50:55
19	Modification Date	2019-02-03T21:20:58
20	Class	
21	Spectrometer Frequency	100.62
22	Spectral Width	24038.5
23	Lowest Frequency	-1958.4
24	Nucleus	13C
25	Acquired Size	32768
26	Spectral Size	65536

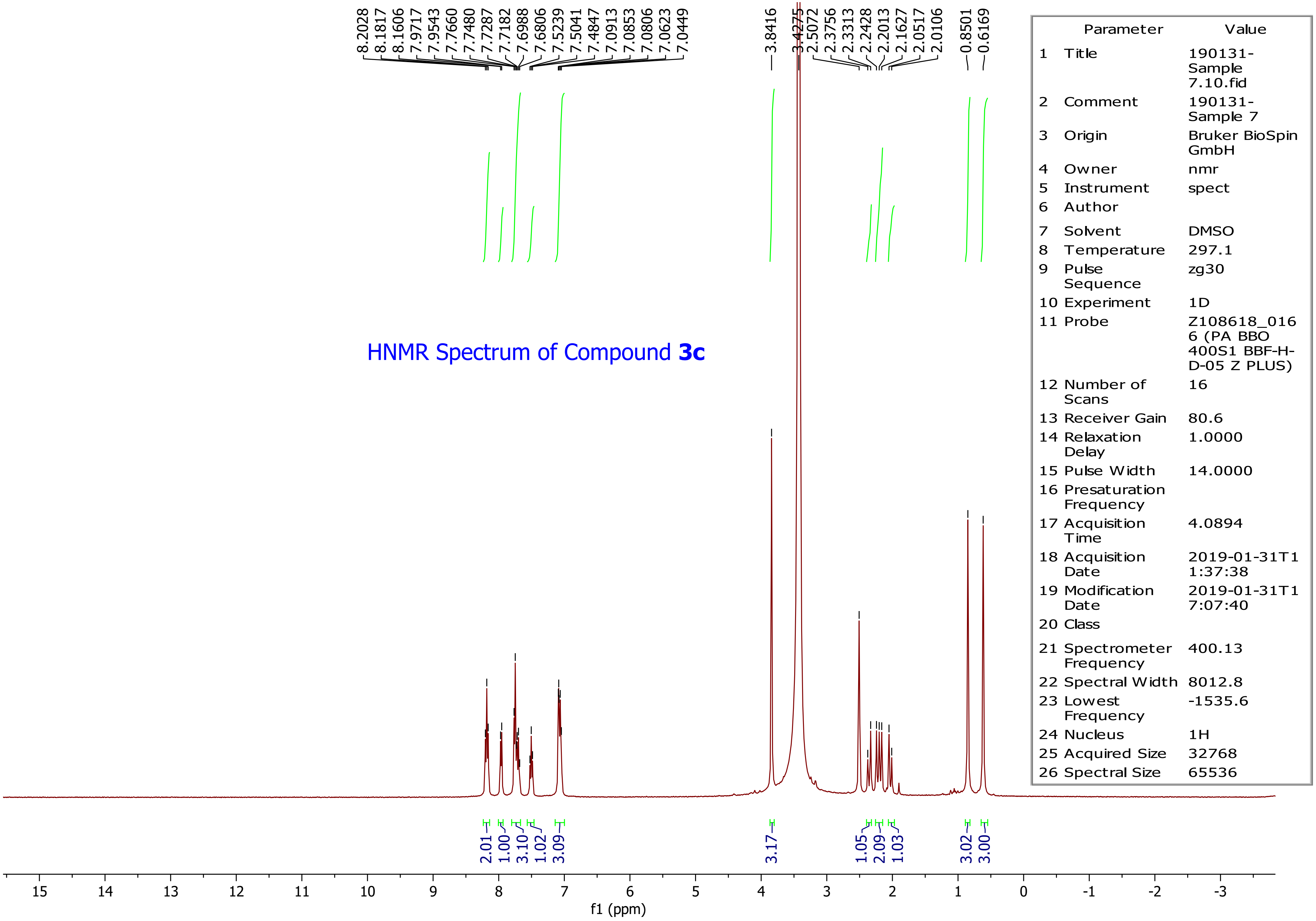

	Parameter	Value
1	Title	190131-Sample 7.10.fid
2	Comment	190131-Sample 7
3	Origin	Bruker BioSpin GmbH
4	Owner	nmr
5	Instrument	spect
6	Author	
7	Solvent	DMSO
8	Temperature	297.1
9	Pulse Sequence	zg30
10	Experiment	1D
11	Probe	Z108618_0166 (PA BBO 400S1 BBF-H-D-05 Z PLUS)
12	Number of Scans	16
13	Receiver Gain	80.6
14	Relaxation Delay	1.0000
15	Pulse Width	14.0000
16	Presaturation Frequency	
17	Acquisition Time	4.0894
18	Acquisition Date	2019-01-31T11:37:38
19	Modification Date	2019-01-31T17:07:40
20	Class	
21	Spectrometer Frequency	400.13
22	Spectral Width	8012.8
23	Lowest Frequency	-1535.6
24	Nucleus	1H
25	Acquired Size	32768
26	Spectral Size	65536

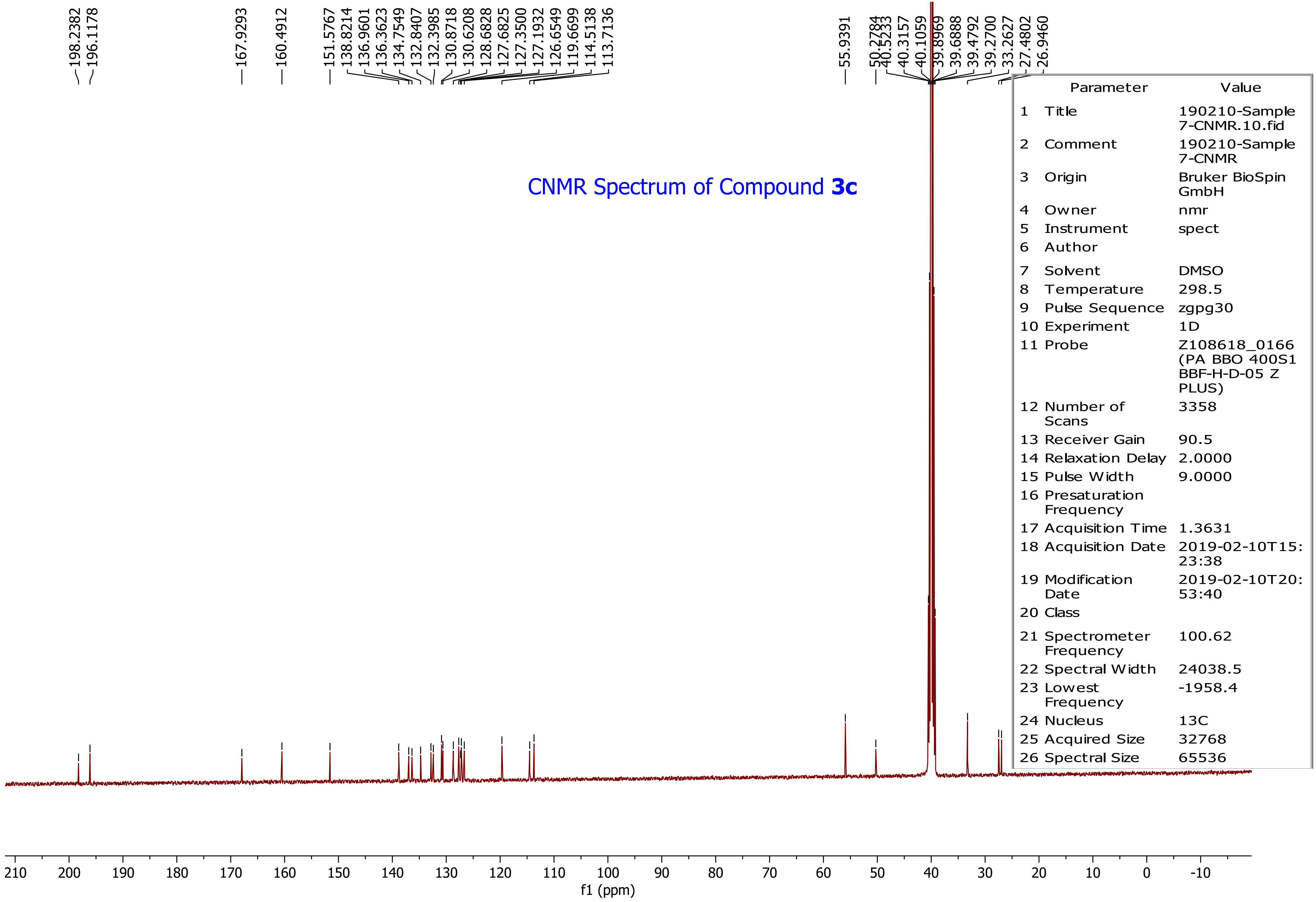

	Parameter	Value
1	Title	190210-Sample 7-CNMR.10.fid
2	Comment	190210-Sample 7-CNMR
3	Origin	Bruker BioSpin GmbH
4	Owner	nmr
5	Instrument	spect
6	Author	
7	Solvent	DMSO
8	Temperature	298.5
9	Pulse Sequence	zgpg30
10	Experiment	1D
11	Probe	Z108618_0166 (PA BBO 400S1 BBF-H-D-05 Z PLUS)
12	Number of Scans	3358
13	Receiver Gain	90.5
14	Relaxation Delay	2.0000
15	Pulse Width	9.0000
16	Presaturation Frequency	
17	Acquisition Time	1.3631
18	Acquisition Date	2019-02-10T15:23:38
19	Modification Date	2019-02-10T20:53:40
20	Class	
21	Spectrometer Frequency	100.62
22	Spectral Width	24038.5
23	Lowest Frequency	-1958.4
24	Nucleus	13C
25	Acquired Size	32768
26	Spectral Size	65536

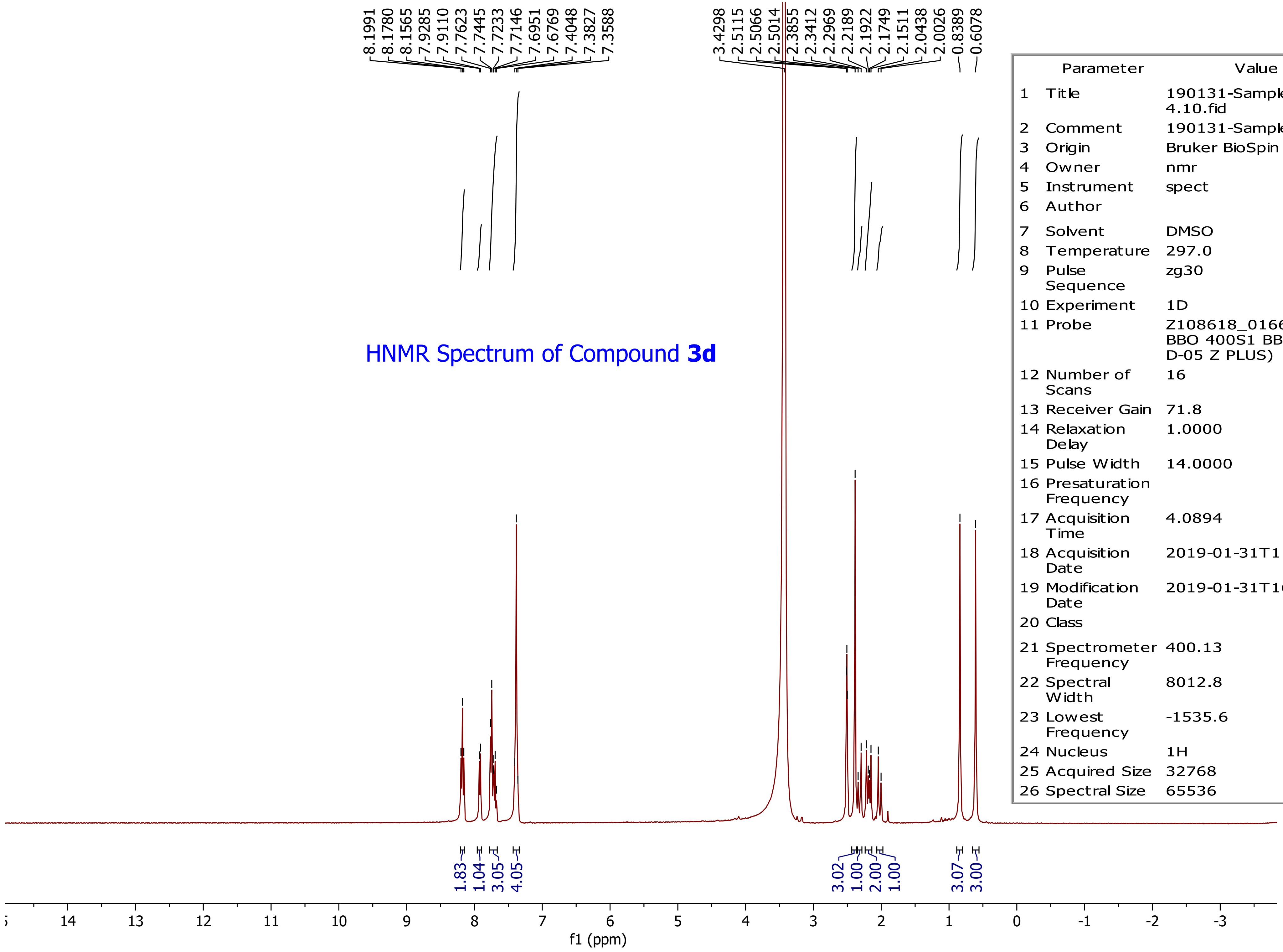

HNMR Spectrum of Compound **3d**

	Parameter	Value
1	Title	190131-Sample 4.10.fid
2	Comment	190131-Sample 4
3	Origin	Bruker BioSpin GmbH
4	Owner	nmr
5	Instrument	spect
6	Author	
7	Solvent	DMSO
8	Temperature	297.0
9	Pulse Sequence	zg30
10	Experiment	1D
11	Probe	Z108618_0166 (PA BBO 400S1 BBF-H-D-05 Z PLUS)
12	Number of Scans	16
13	Receiver Gain	71.8
14	Relaxation Delay	1.0000
15	Pulse Width	14.0000
16	Presaturation Frequency	
17	Acquisition Time	4.0894
18	Acquisition Date	2019-01-31T11:28:16
19	Modification Date	2019-01-31T16:58:18
20	Class	
21	Spectrometer Frequency	400.13
22	Spectral Width	8012.8
23	Lowest Frequency	-1535.6
24	Nucleus	1H
25	Acquired Size	32768
26	Spectral Size	65536

CNMR Spectrum of Compound **3d**

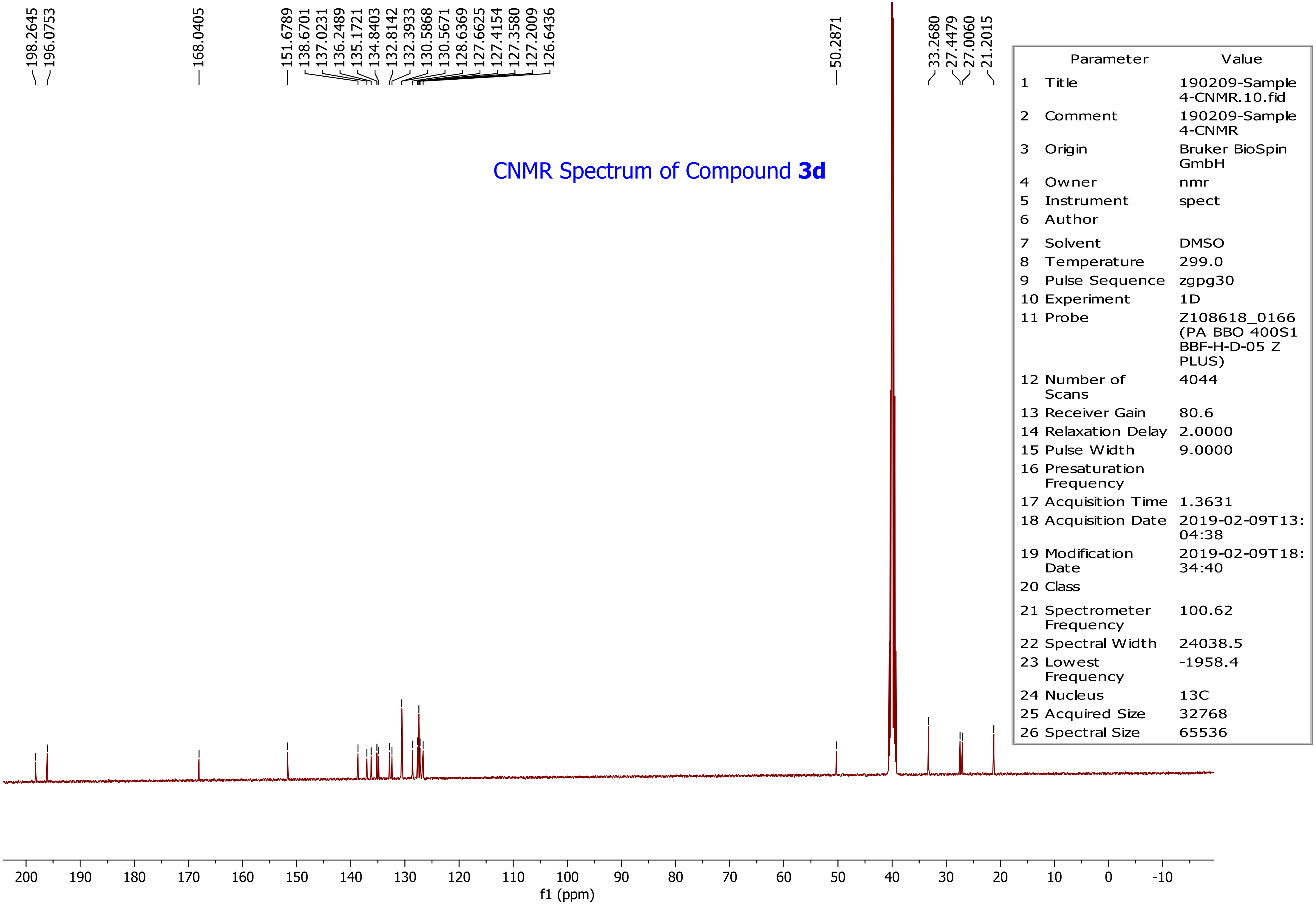

HNMR Spectrum of Compound **3e**

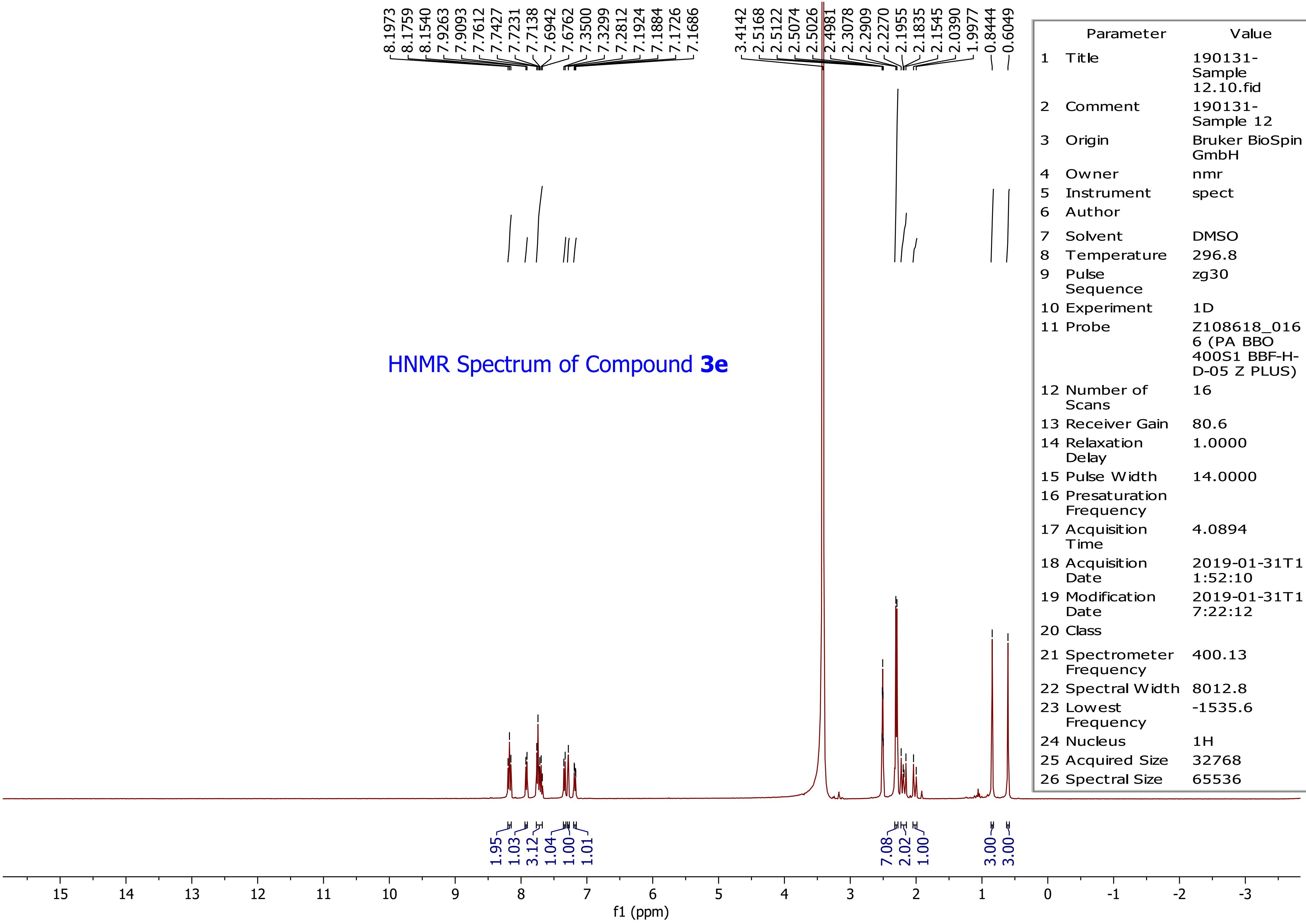

	Parameter	Value
1	Title	190131-Sample 12.10.fid
2	Comment	190131-Sample 12
3	Origin	Bruker BioSpin GmbH
4	Owner	nmr
5	Instrument	spect
6	Author	
7	Solvent	DMSO
8	Temperature	296.8
9	Pulse Sequence	zg30
10	Experiment	1D
11	Probe	Z108618_0166 (PA BBO 400S1 BBF-H-D-05 Z PLUS)
12	Number of Scans	16
13	Receiver Gain	80.6
14	Relaxation Delay	1.0000
15	Pulse Width	14.0000
16	Presaturation Frequency	
17	Acquisition Time	4.0894
18	Acquisition Date	2019-01-31T11:52:10
19	Modification Date	2019-01-31T17:22:12
20	Class	
21	Spectrometer Frequency	400.13
22	Spectral Width	8012.8
23	Lowest Frequency	-1535.6
24	Nucleus	1H
25	Acquired Size	32768
26	Spectral Size	65536

CNMR Spectrum of Compound **3e**

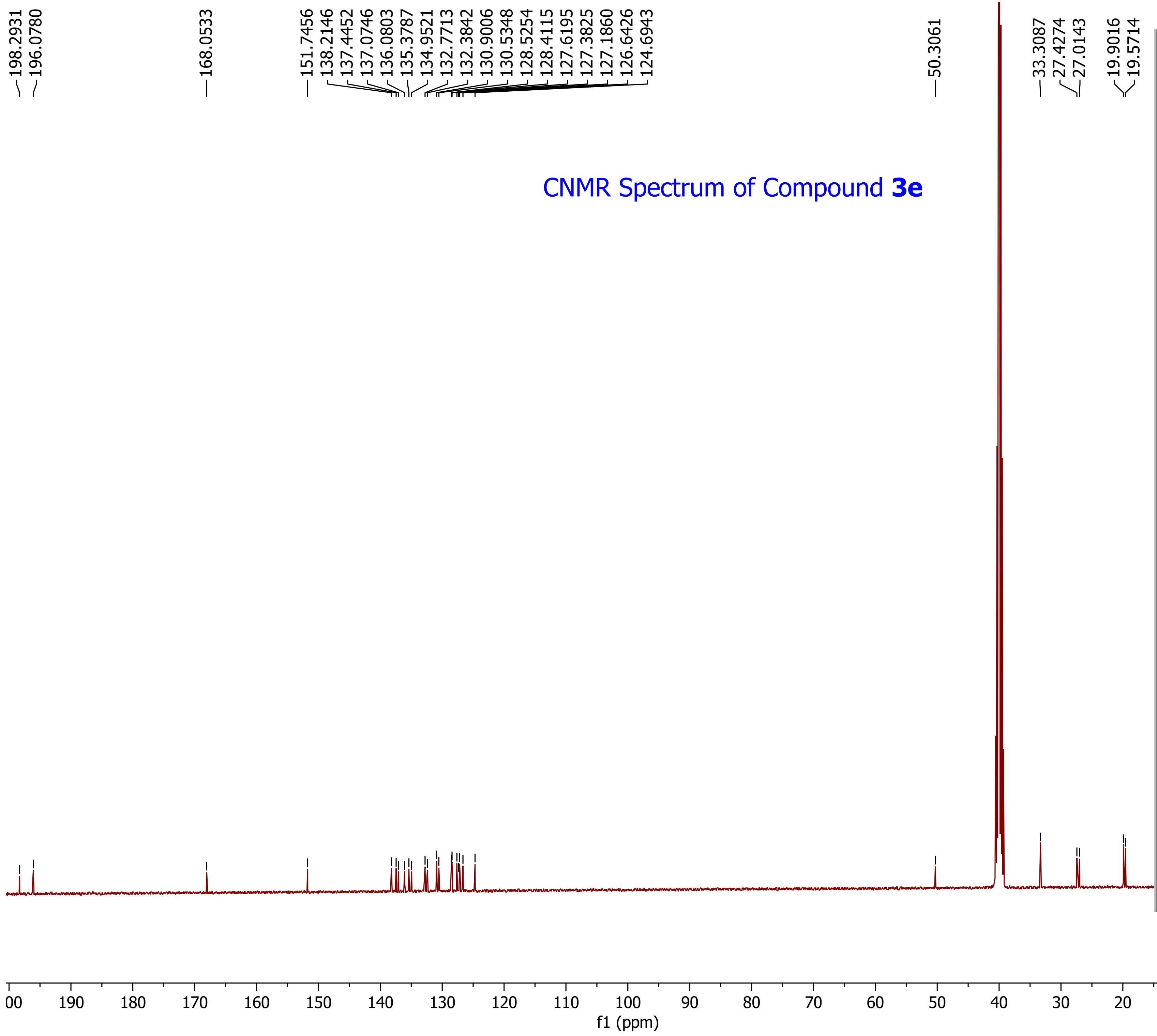

	Parameter	Value
1	Title	190216-Sample 12-CNMR.10.fid
2	Comment	190216-Sample 12-CNMR
3	Origin	Bruker BioSpin GmbH
4	Owner	nmr
5	Instrument	spect
6	Author	
7	Solvent	DMSO
8	Temperature	298.7
9	Pulse Sequence	zgpg30
10	Experiment	1D
11	Probe	Z108618_0166 (PA BBO 400S1 BBF-H-D-05 Z PLUS)
12	Number of Scans	4347
13	Receiver Gain	80.6
14	Relaxation Delay	2.0000
15	Pulse Width	9.0000
16	Presaturation Frequency	
17	Acquisition Time	1.3631
18	Acquisition Date	2019-02-16T12:06:52
19	Modification Date	2019-02-16T17:36:54
20	Class	
21	Spectrometer Frequency	100.62
22	Spectral Width	24038.5
23	Lowest Frequency	-1958.4
24	Nucleus	13C
25	Acquired Size	32768
26	Spectral Size	65536

HNMR Spectrum of Compound **3f**

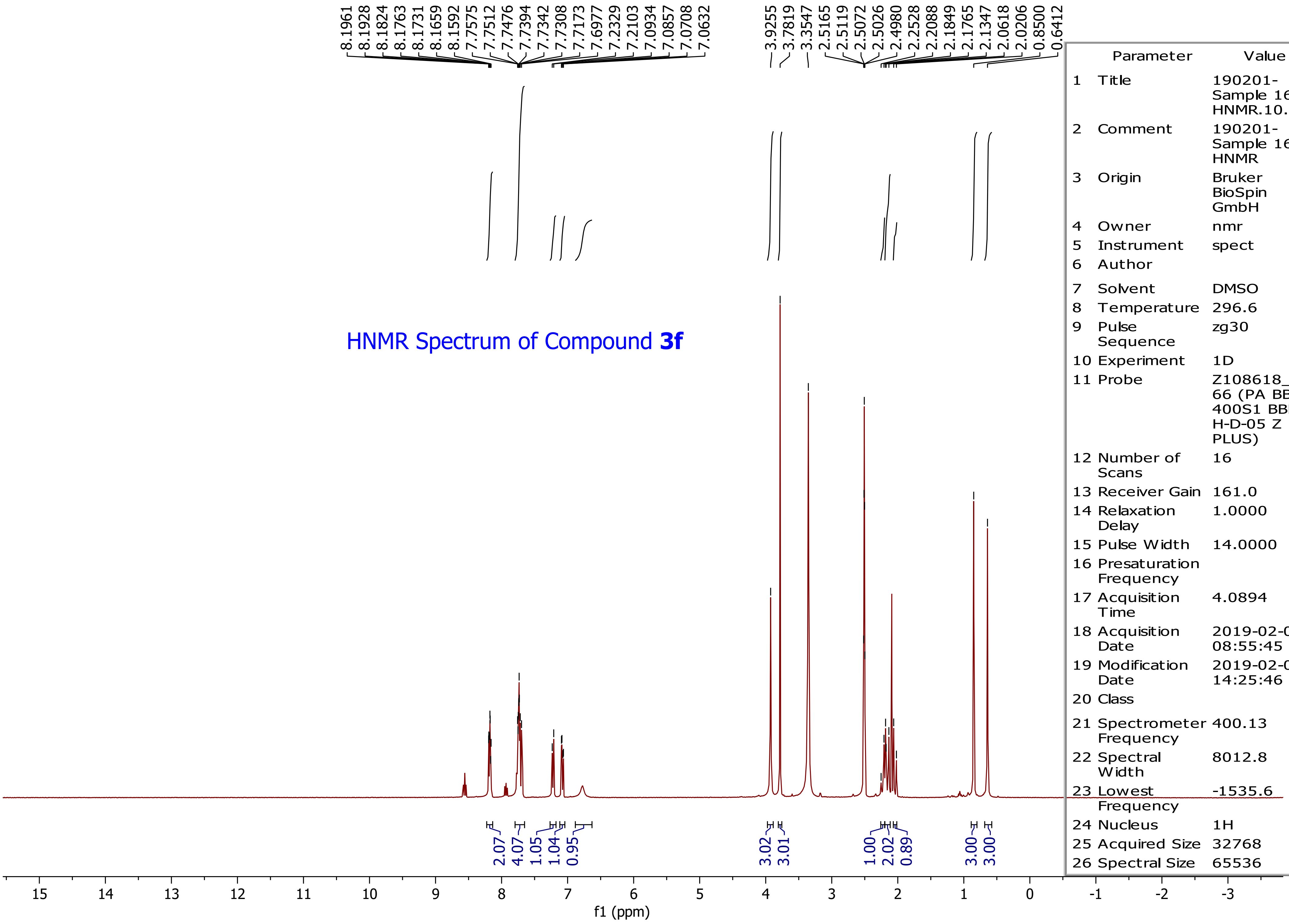

Parameter		Value
1	Title	190201-Sample 16-HNMR.10.fid
2	Comment	190201-Sample 16-HNMR
3	Origin	Bruker BioSpin GmbH
4	Owner	nmr
5	Instrument	spect
6	Author	
7	Solvent	DMSO
8	Temperature	296.6
9	Pulse Sequence	zg30
10	Experiment	1D
11	Probe	Z108618_0166 (PA BBO 400S1 BBF-H-D-05 Z PLUS)
12	Number of Scans	16
13	Receiver Gain	161.0
14	Relaxation Delay	1.0000
15	Pulse Width	14.0000
16	Presaturation Frequency	
17	Acquisition Time	4.0894
18	Acquisition Date	2019-02-01T08:55:45
19	Modification Date	2019-02-01T14:25:46
20	Class	
21	Spectrometer Frequency	400.13
22	Spectral Width	8012.8
23	Lowest Frequency	-1535.6
24	Nucleus	1H
25	Acquired Size	32768
26	Spectral Size	65536

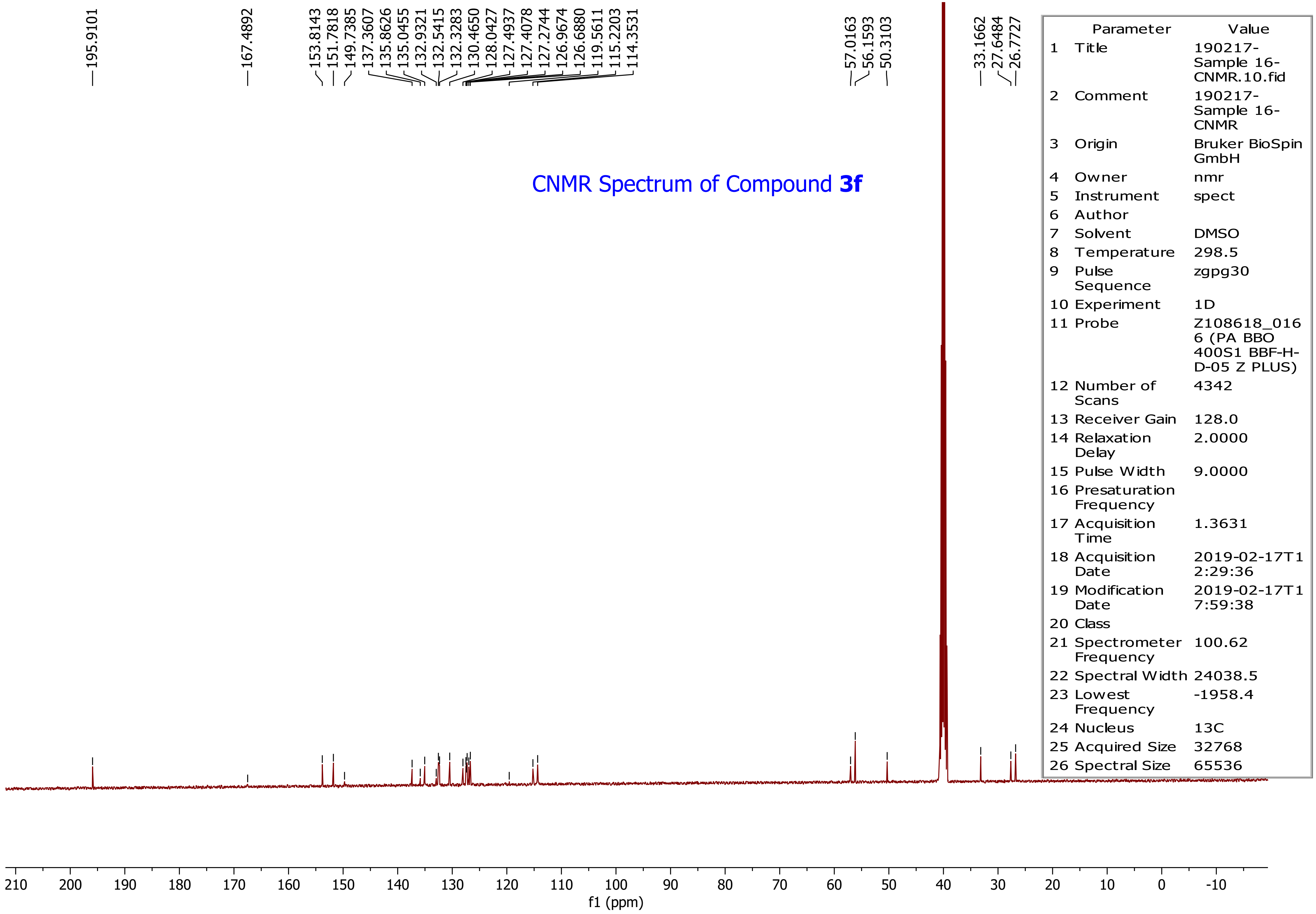

	Parameter	Value
1	Title	190217-Sample 16-CNMR.10.fid
2	Comment	190217-Sample 16-CNMR
3	Origin	Bruker BioSpin GmbH
4	Owner	nmr
5	Instrument	spect
6	Author	
7	Solvent	DMSO
8	Temperature	298.5
9	Pulse Sequence	zgpg30
10	Experiment	1D
11	Probe	Z108618_0166 (PA BBO 400S1 BBF-H-D-05 Z PLUS)
12	Number of Scans	4342
13	Receiver Gain	128.0
14	Relaxation Delay	2.0000
15	Pulse Width	9.0000
16	Presaturation Frequency	
17	Acquisition Time	1.3631
18	Acquisition Date	2019-02-17T12:29:36
19	Modification Date	2019-02-17T17:59:38
20	Class	
21	Spectrometer Frequency	100.62
22	Spectral Width	24038.5
23	Lowest Frequency	-1958.4
24	Nucleus	13C
25	Acquired Size	32768
26	Spectral Size	65536

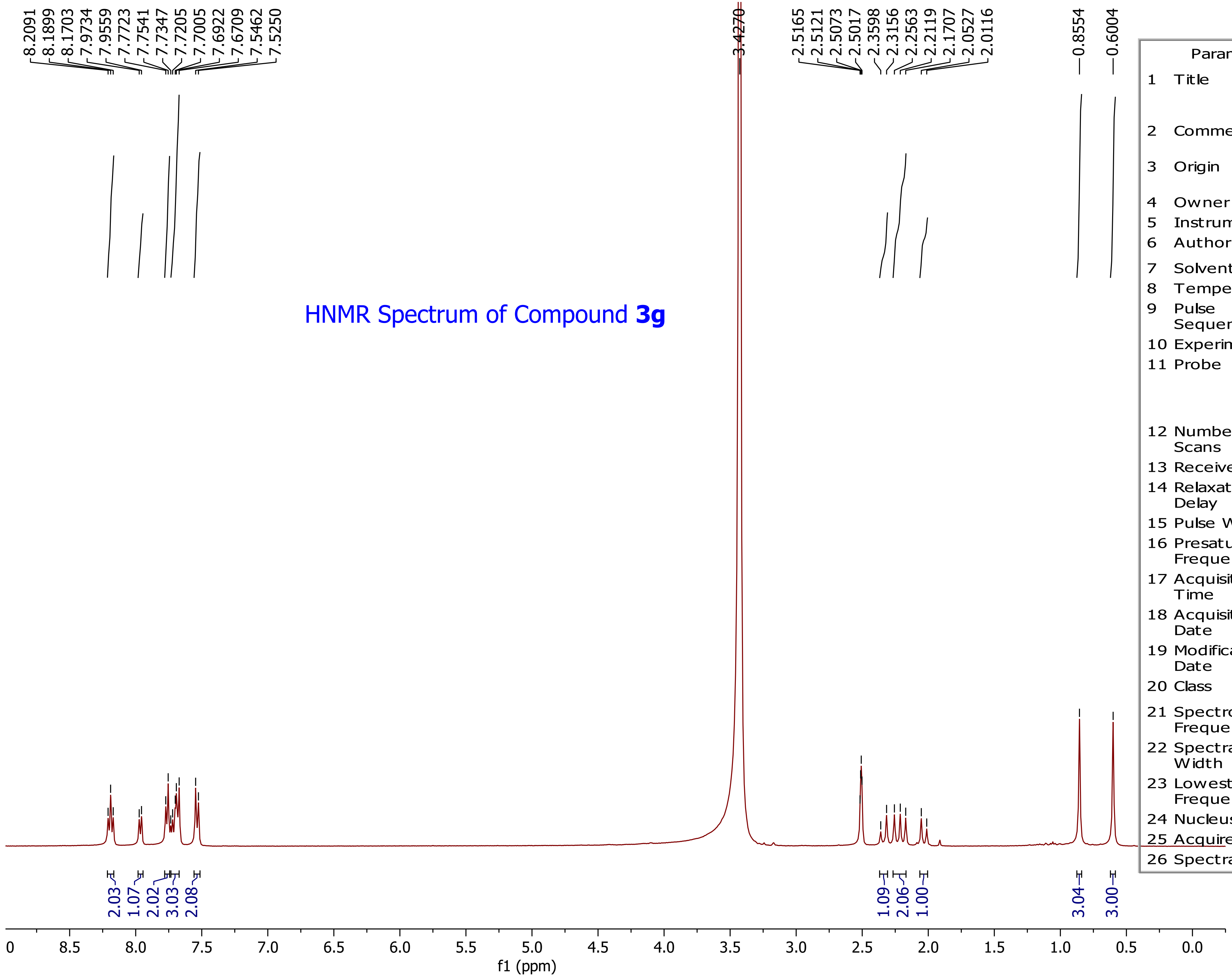

	Parameter	Value
1	Title	190131-Sample 8.10.fid
2	Comment	190131-Sample 8
3	Origin	Bruker BioSpin GmbH
4	Owner	nmr
5	Instrument	spect
6	Author	
7	Solvent	DMSO
8	Temperature	297.1
9	Pulse Sequence	zg30
10	Experiment	1D
11	Probe	Z108618_0166 (PA BBO 400S1 BBF-H-D-05 Z PLUS)
12	Number of Scans	16
13	Receiver Gain	80.6
14	Relaxation Delay	1.0000
15	Pulse Width	14.0000
16	Presaturation Frequency	
17	Acquisition Time	4.0894
18	Acquisition Date	2019-01-31T11:42:41
19	Modification Date	2019-01-31T17:12:44
20	Class	
21	Spectrometer Frequency	400.13
22	Spectral Width	8012.8
23	Lowest Frequency	-1535.6
24	Nucleus	1H
25	Acquired Size	32768
26	Spectral Size	65536

CNMR Spectrum of Compound **3g**

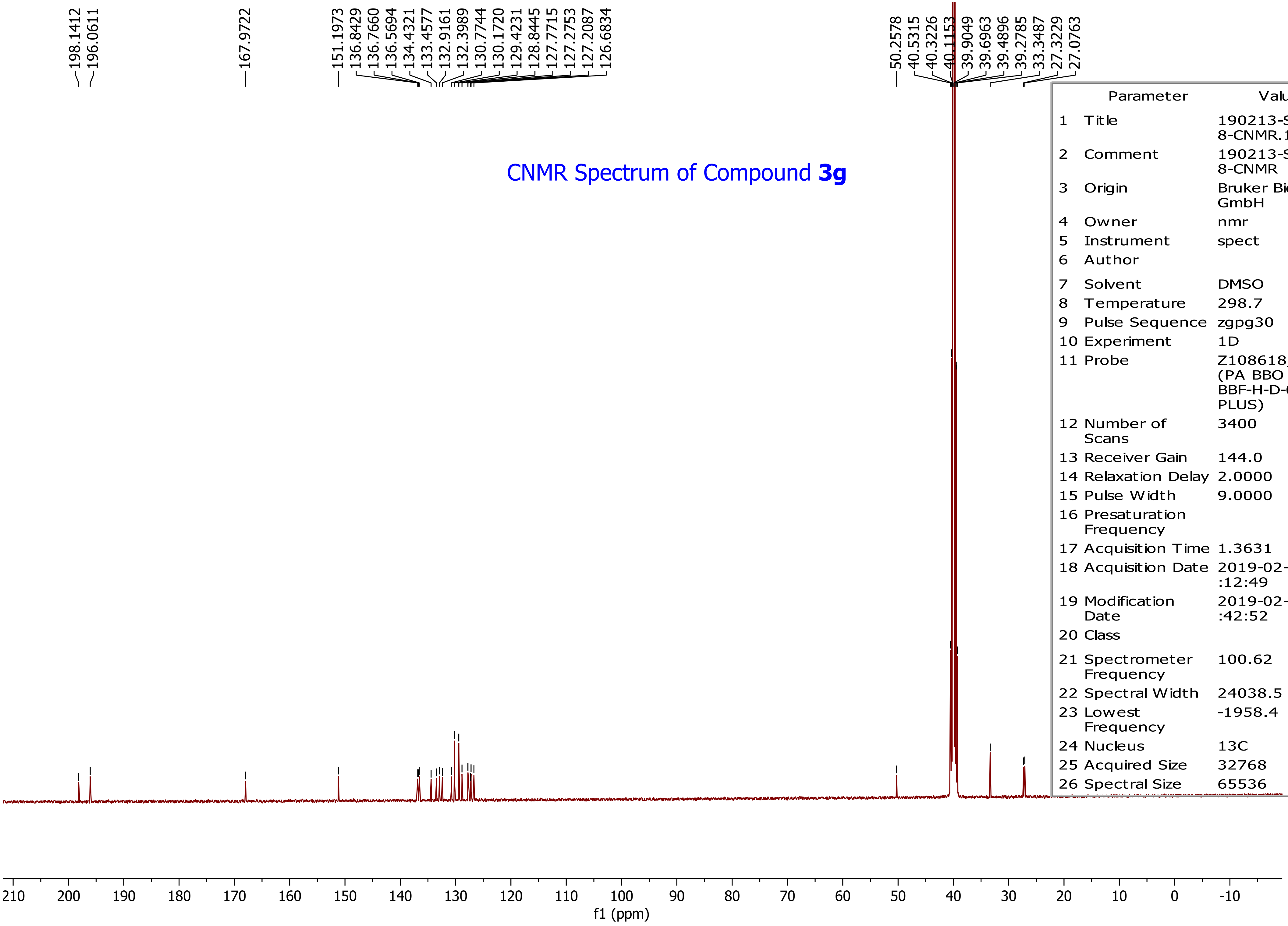

	Parameter	Value
1	Title	190213-Sample 8-CNMR.10.fid
2	Comment	190213-Sample 8-CNMR
3	Origin	Bruker BioSpin GmbH
4	Owner	nmr
5	Instrument	spect
6	Author	
7	Solvent	DMSO
8	Temperature	298.7
9	Pulse Sequence	zgpg30
10	Experiment	1D
11	Probe	Z108618_0166 (PA BBO 400S1 BBF-H-D-05 Z PLUS)
12	Number of Scans	3400
13	Receiver Gain	144.0
14	Relaxation Delay	2.0000
15	Pulse Width	9.0000
16	Presaturation Frequency	
17	Acquisition Time	1.3631
18	Acquisition Date	2019-02-13T15:12:49
19	Modification Date	2019-02-13T20:42:52
20	Class	
21	Spectrometer Frequency	100.62
22	Spectral Width	24038.5
23	Lowest Frequency	-1958.4
24	Nucleus	13C
25	Acquired Size	32768
26	Spectral Size	65536

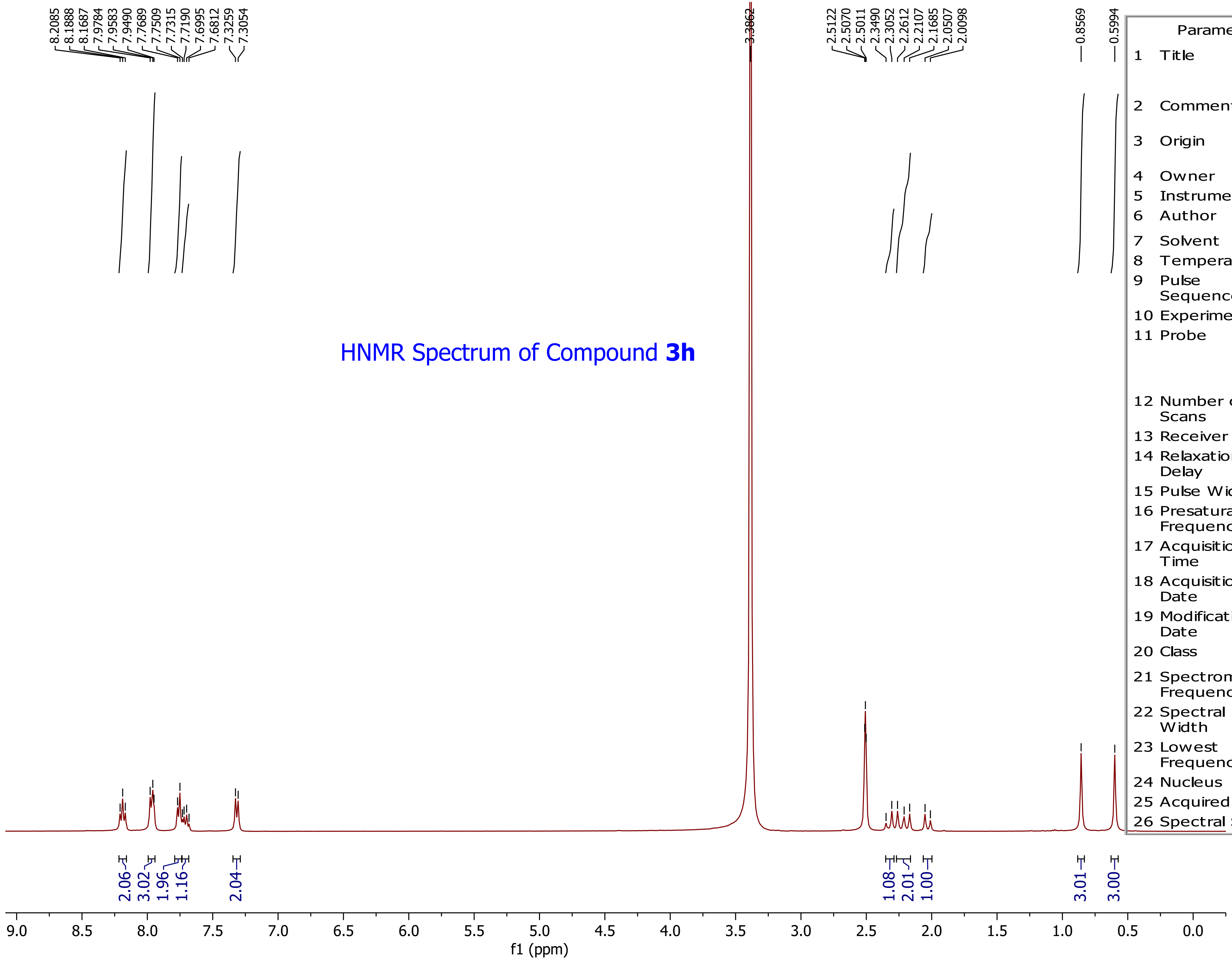

HNMR Spectrum of Compound **3h**

	Parameter	Value
1	Title	190131-Sample 13.10.fid
2	Comment	190131-Sample 13
3	Origin	Bruker BioSpin GmbH
4	Owner	nmr
5	Instrument	spect
6	Author	
7	Solvent	DMSO
8	Temperature	296.7
9	Pulse Sequence	zg30
10	Experiment	1D
11	Probe	Z108618_0166 (PA BBO 400S1 BBF-H-D-05 Z PLUS)
12	Number of Scans	16
13	Receiver Gain	114.0
14	Relaxation Delay	1.0000
15	Pulse Width	14.0000
16	Presaturation Frequency	
17	Acquisition Time	4.0894
18	Acquisition Date	2019-01-31T 11:57:13
19	Modification Date	2019-01-31T 17:27:14
20	Class	
21	Spectrometer Frequency	400.13
22	Spectral Width	8012.8
23	Lowest Frequency	-1535.6
24	Nucleus	1H
25	Acquired Size	32768
26	Spectral Size	65536

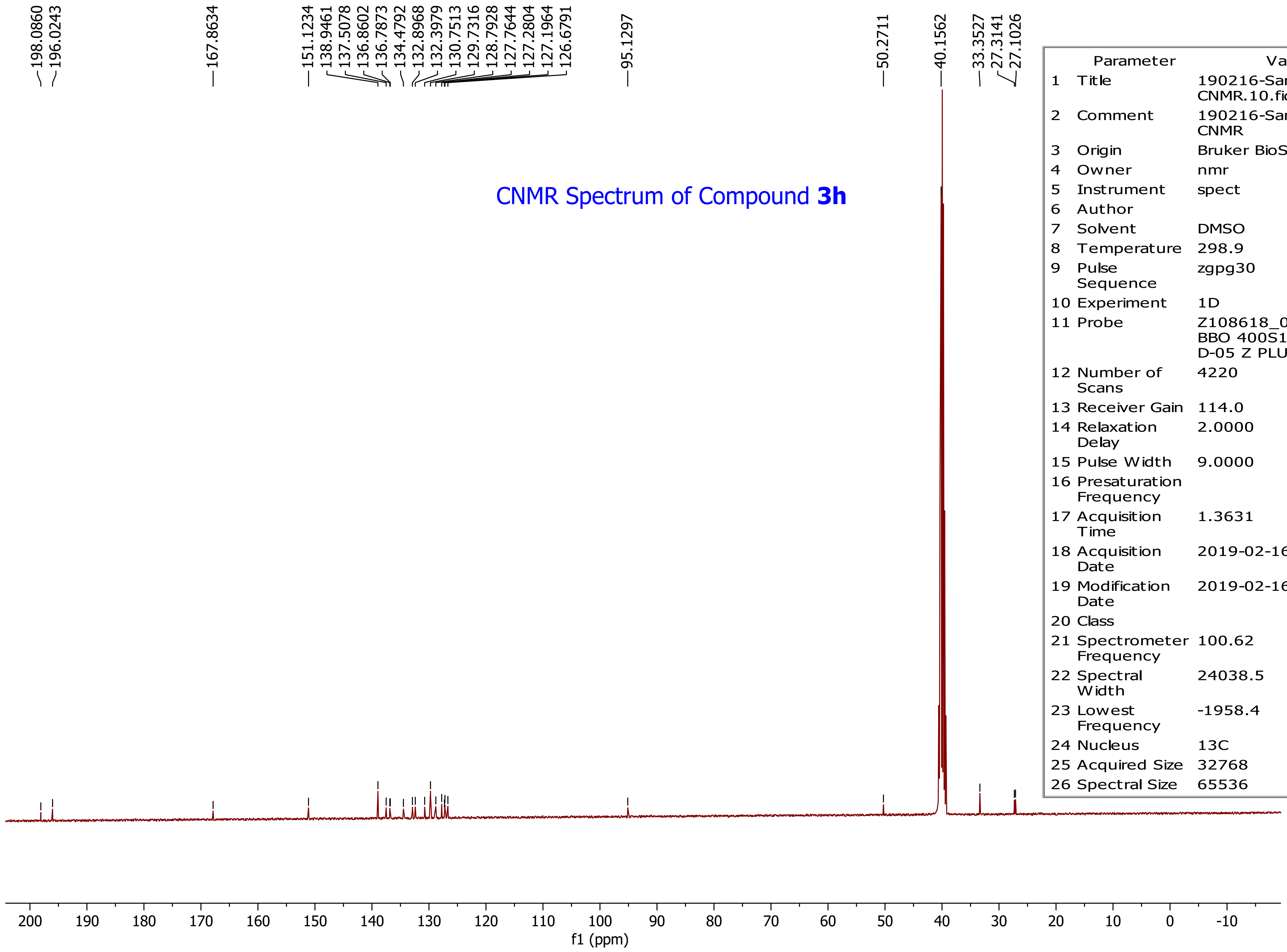

	Parameter	Value
1	Title	190216-Sample 13-CNMR.10.fid
2	Comment	190216-Sample 13-CNMR
3	Origin	Bruker BioSpin GmbH
4	Owner	nmr
5	Instrument	spect
6	Author	
7	Solvent	DMSO
8	Temperature	298.9
9	Pulse Sequence	zgpg30
10	Experiment	1D
11	Probe	Z108618_0166 (PA BBO 400S1 BBF-H-D-05 Z PLUS)
12	Number of Scans	4220
13	Receiver Gain	114.0
14	Relaxation Delay	2.0000
15	Pulse Width	9.0000
16	Presaturation Frequency	
17	Acquisition Time	1.3631
18	Acquisition Date	2019-02-16T16:16:10
19	Modification Date	2019-02-16T21:46:12
20	Class	
21	Spectrometer Frequency	100.62
22	Spectral Width	24038.5
23	Lowest Frequency	-1958.4
24	Nucleus	13C
25	Acquired Size	32768
26	Spectral Size	65536

HNMR Spectrum of Compound **3i**

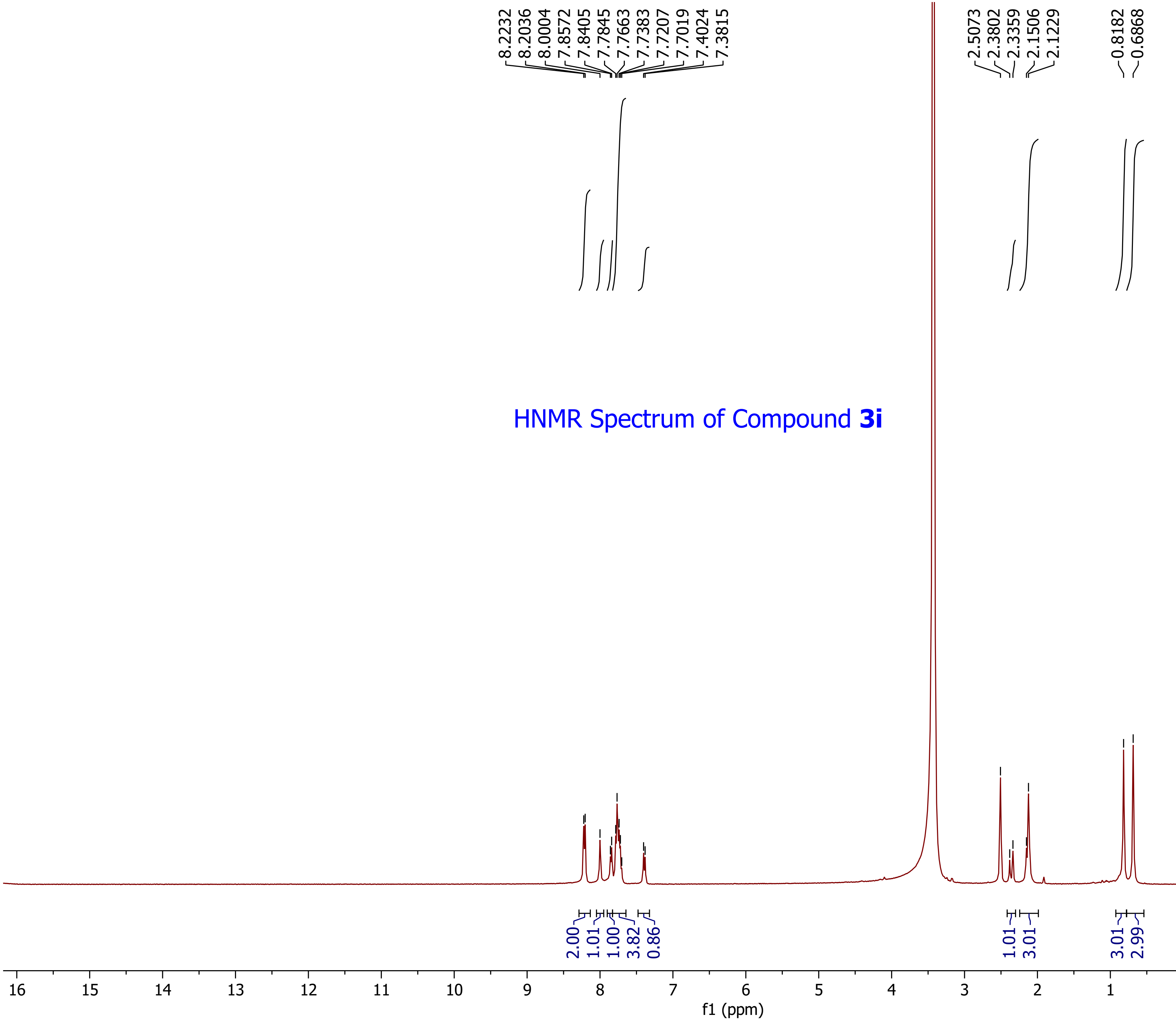

	Parameter	Value
1	Title	190131-Sample 6.10.fid
2	Comment	190131-Sample 6
3	Origin	Bruker BioSpin GmbH
4	Owner	nmr
5	Instrument	spect
6	Author	
7	Solvent	DMSO
8	Temperature	297.0
9	Pulse Sequence	zg30
10	Experiment	1D
11	Probe	Z108618_0166 (PA BBO 400S1 BBF-H-D-05 Z PLUS)
12	Number of Scans	16
13	Receiver Gain	80.6
14	Relaxation Delay	1.0000
15	Pulse Width	14.0000
16	Presaturation Frequency	
17	Acquisition Time	4.0894
18	Acquisition Date	2019-01-31T11:33:11
19	Modification Date	2019-01-31T17:03:14
20	Class	
21	Spectrometer Frequency	400.13
22	Spectral Width	8012.8
23	Lowest Frequency	-1535.6
24	Nucleus	1H
25	Acquired Size	32768
26	Spectral Size	65536

CNMR Spectrum of Compound **3i**

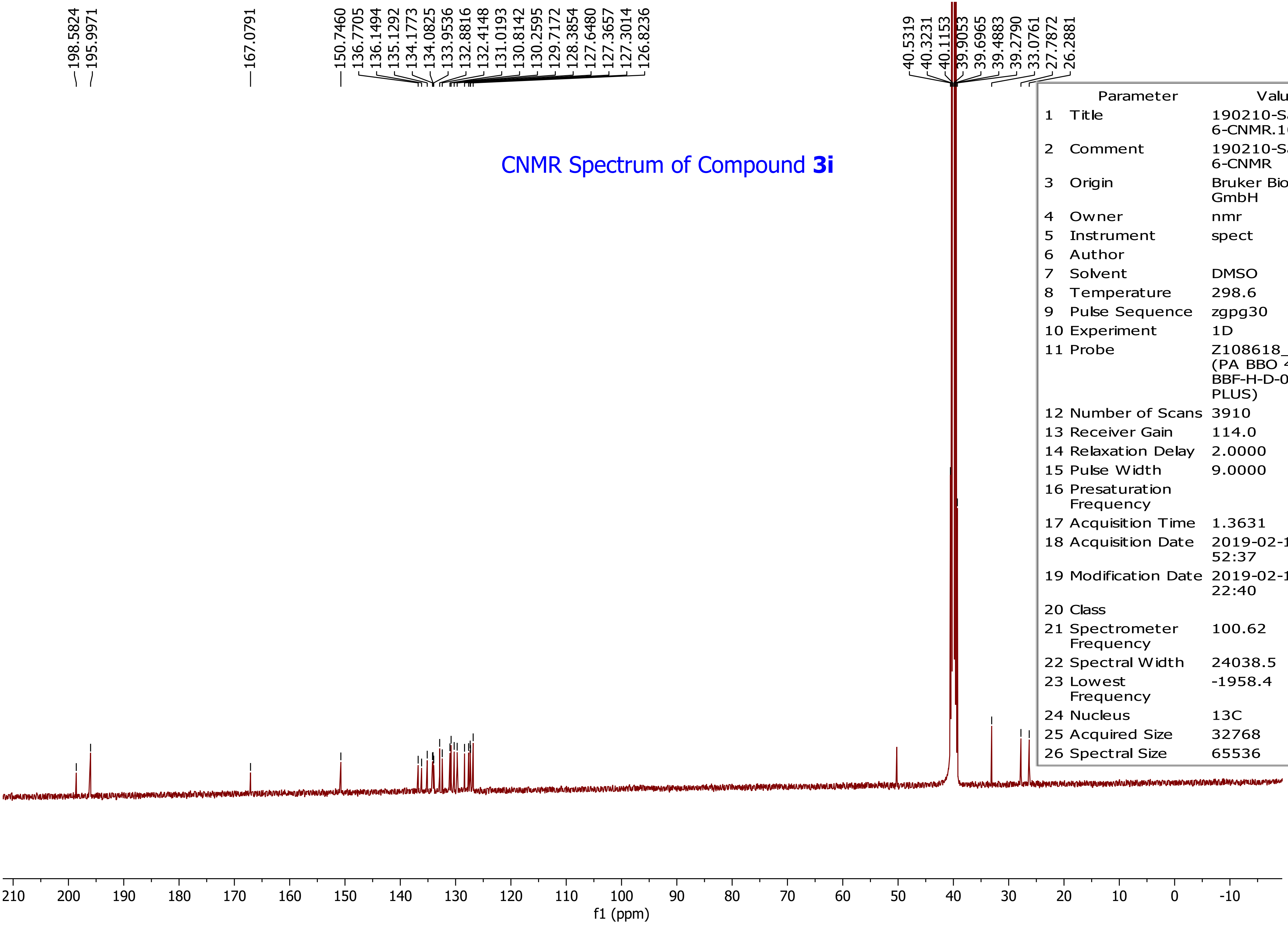

	Parameter	Value
1	Title	190210-Sample 6-CNMR.10.fid
2	Comment	190210-Sample 6-CNMR
3	Origin	Bruker BioSpin GmbH
4	Owner	nmr
5	Instrument	spect
6	Author	
7	Solvent	DMSO
8	Temperature	298.6
9	Pulse Sequence	zgpg30
10	Experiment	1D
11	Probe	Z108618_0166 (PA BBO 400S1 BBF-H-D-05 Z PLUS)
12	Number of Scans	3910
13	Receiver Gain	114.0
14	Relaxation Delay	2.0000
15	Pulse Width	9.0000
16	Presaturation Frequency	
17	Acquisition Time	1.3631
18	Acquisition Date	2019-02-10T10:52:37
19	Modification Date	2019-02-10T16:22:40
20	Class	
21	Spectrometer Frequency	100.62
22	Spectral Width	24038.5
23	Lowest Frequency	-1958.4
24	Nucleus	13C
25	Acquired Size	32768
26	Spectral Size	65536

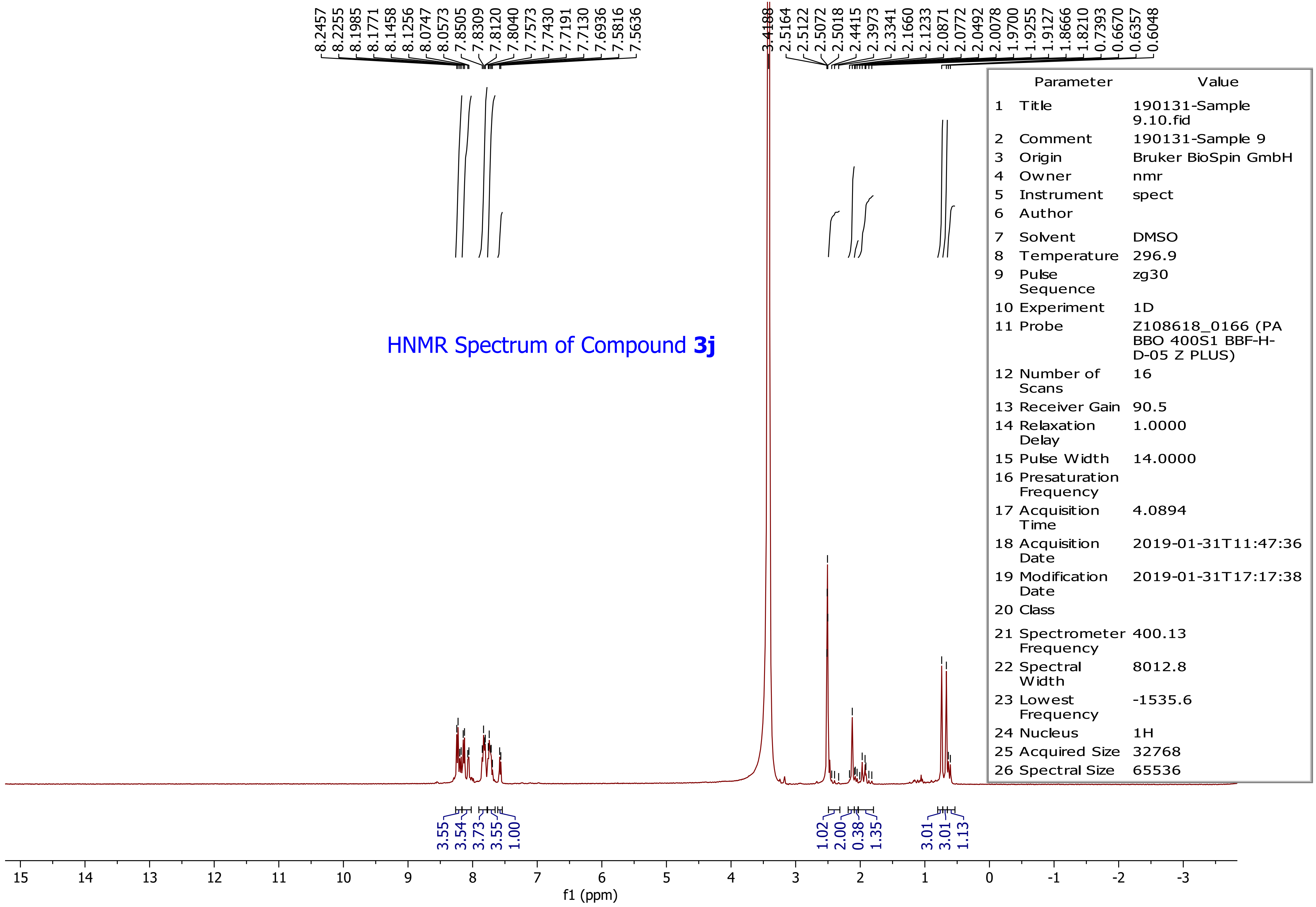
HNMR Spectrum of Compound 3j
8.2457 8.2255 8.1985 8.1771 8.1458 8.1256 8.0747 8.0573 7.8505 7.8309 7.8120 7.8040 7.7573 7.7430 7.7191 7.7130 7.6936 7.5816 7.5636
3.4188 2.5164 2.5122 2.5072 2.5018 2.4415 2.3973 2.3341 2.1660 2.1233 2.0871 2.0772 2.0492 2.0078 1.9700 1.9255 1.9127 1.8666 1.8210 0.7393 0.6670 0.6357 0.6048
3.55 3.54 3.73 3.55 1.00
1.02 2.00 0.38 1.35
3.01 3.01 1.13
15 14 13 12 11 10 9 8 7 6 5 4 3 2 1 0 -1 -2 -3
f1 (ppm)
Parameter Value
1 Title 190131-Sample 9.10.fid
2 Comment 190131-Sample 9
3 Origin Bruker BioSpin GmbH
4 Owner nmr
5 Instrument spect
6 Author
7 Solvent DMSO
8 Temperature 296.9
9 Pulse Sequence zg30
10 Experiment 1D
11 Probe Z108618_0166 (PA BBO 400S1 BBF-H-D-05 Z PLUS)
12 Number of Scans 16
13 Receiver Gain 90.5
14 Relaxation Delay 1.0000
15 Pulse Width 14.0000
16 Presaturation Frequency
17 Acquisition Time 4.0894
18 Acquisition Date 2019-01-31T11:47:36
19 Modification Date 2019-01-31T17:17:38
20 Class
21 Spectrometer Frequency 400.13
22 Spectral Width 8012.8
23 Lowest Frequency -1535.6
24 Nucleus 1H
25 Acquired Size 32768
26 Spectral Size 65536

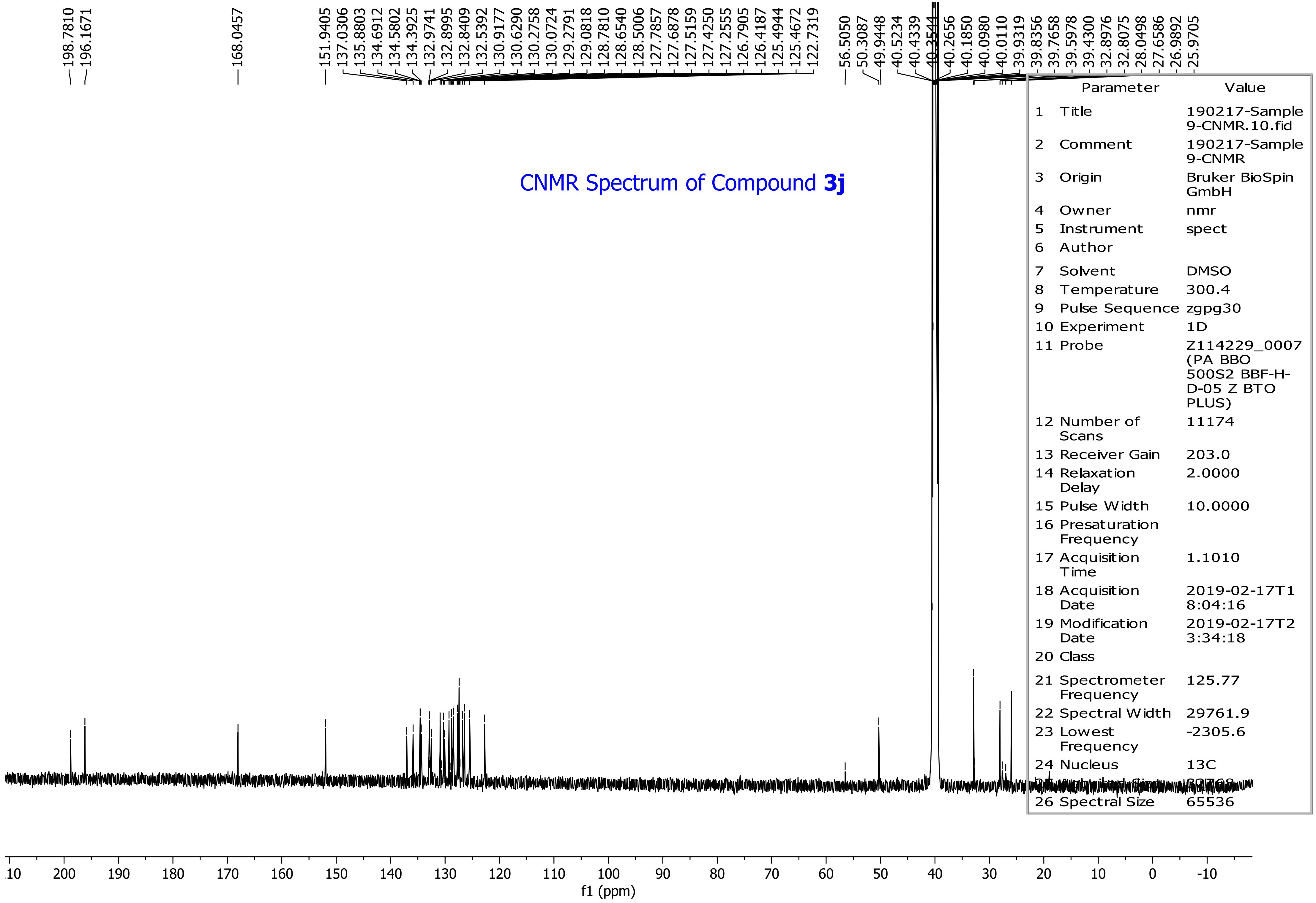
198.7810
196.1671
168.0457
151.9405
137.0306
135.8803
134.6912
134.5802
134.3925
132.9741
132.8995
132.8409
132.5392
130.9177
130.6290
130.2758
130.0724
129.2791
129.0818
128.7810
128.6540
128.5006
127.7857
127.6878
127.5159
127.4250
127.2555
126.7905
126.4187
125.4944
125.4672
122.7319
56.5050
50.3087
49.9448
40.5234
40.4339
40.3544
40.2656
40.1850
40.0980
40.0110
39.9319
39.8356
39.7658
39.5978
39.4300
32.8976
32.8075
28.0498
27.6586
26.9892
25.9705
CNMR Spectrum of Compound 3j
Parameter Value
1 Title 190217-Sample 9-CNMR.10.fid
2 Comment 190217-Sample 9-CNMR
3 Origin Bruker BioSpin GmbH
4 Owner nmr
5 Instrument spect
6 Author
7 Solvent DMSO
8 Temperature 300.4
9 Pulse Sequence zgpg30
10 Experiment 1D
11 Probe Z114229_0007 (PA BBO 500S2 BBF-H-D-05 Z BTO PLUS)
12 Number of Scans 11174
13 Receiver Gain 203.0
14 Relaxation Delay 2.0000
15 Pulse Width 10.0000
16 Presaturation Frequency
17 Acquisition Time 1.1010
18 Acquisition Date 2019-02-17T18:04:16
19 Modification Date 2019-02-17T23:34:18
20 Class
21 Spectrometer Frequency 125.77
22 Spectral Width 29761.9
23 Lowest Frequency -2305.6
24 Nucleus 13C
26 Spectral Size 65536
f1 (ppm)

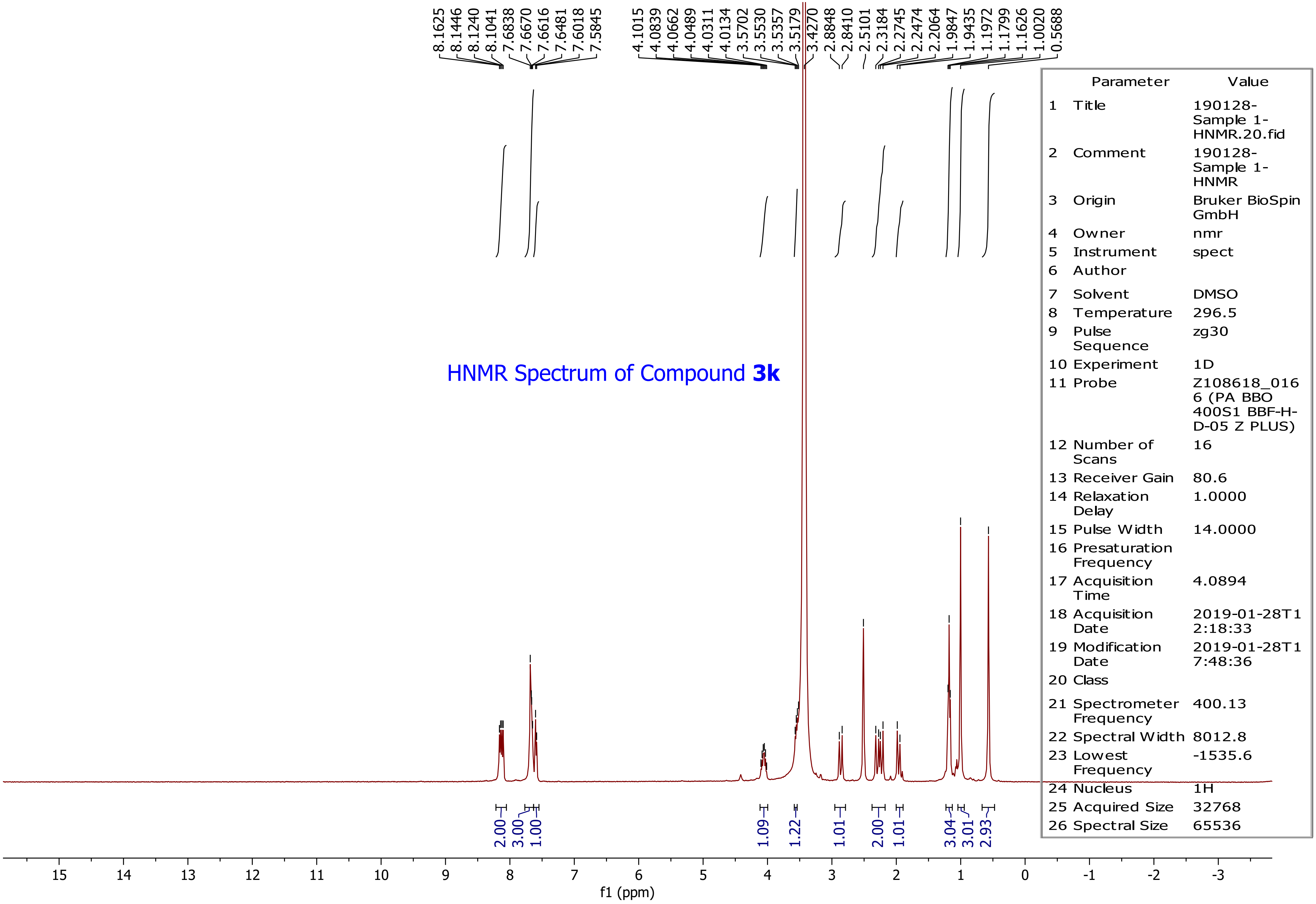

HNMR Spectrum of Compound **3k**

	Parameter	Value
1	Title	190128-Sample 1-HNMR.20.fid
2	Comment	190128-Sample 1-HNMR
3	Origin	Bruker BioSpin GmbH
4	Owner	nmr
5	Instrument	spect
6	Author	
7	Solvent	DMSO
8	Temperature	296.5
9	Pulse Sequence	zg30
10	Experiment	1D
11	Probe	Z108618_0166 (PA BBO 400S1 BBF-H-D-05 Z PLUS)
12	Number of Scans	16
13	Receiver Gain	80.6
14	Relaxation Delay	1.0000
15	Pulse Width	14.0000
16	Presaturation Frequency	
17	Acquisition Time	4.0894
18	Acquisition Date	2019-01-28T12:18:33
19	Modification Date	2019-01-28T17:48:36
20	Class	
21	Spectrometer Frequency	400.13
22	Spectral Width	8012.8
23	Lowest Frequency	-1535.6
24	Nucleus	1H
25	Acquired Size	32768
26	Spectral Size	65536

CNMR Spectrum of Compound **3k**

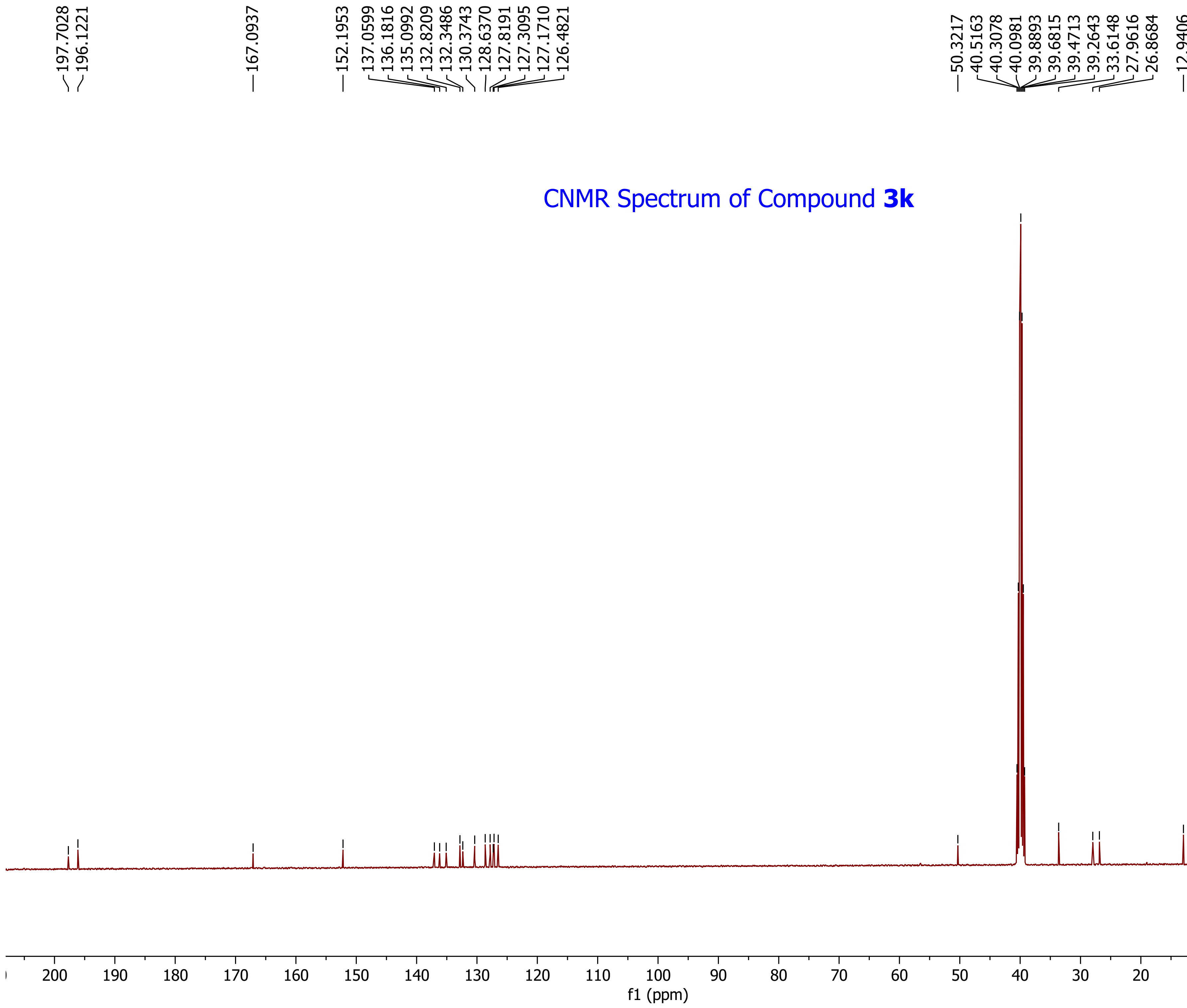

	Parameter	Value
1	Title	190203-Sample 1-CNMR.10.fid
2	Comment	190203-Sample 1-CNMR
3	Origin	Bruker BioSpin GmbH
4	Owner	nmr
5	Instrument	spect
6	Author	
7	Solvent	DMSO
8	Temperature	297.8
9	Pulse Sequence	zgpg30
10	Experiment	1D
11	Probe	Z108618_0166 (PA BBO 400S1 BBF-H-D-05 Z PLUS)
12	Number of Scans	4431
13	Receiver Gain	114.0
14	Relaxation Delay	2.0000
15	Pulse Width	9.0000
16	Presaturation Frequency	
17	Acquisition Time	1.3631
18	Acquisition Date	2019-02-03T11:43:44
19	Modification Date	2019-02-03T17:13:46
20	Class	
21	Spectrometer Frequency	100.62
22	Spectral Width	24038.5
23	Lowest Frequency	-1958.4
24	Nucleus	13C
25	Acquired Size	32768
26	Spectral Size	65536

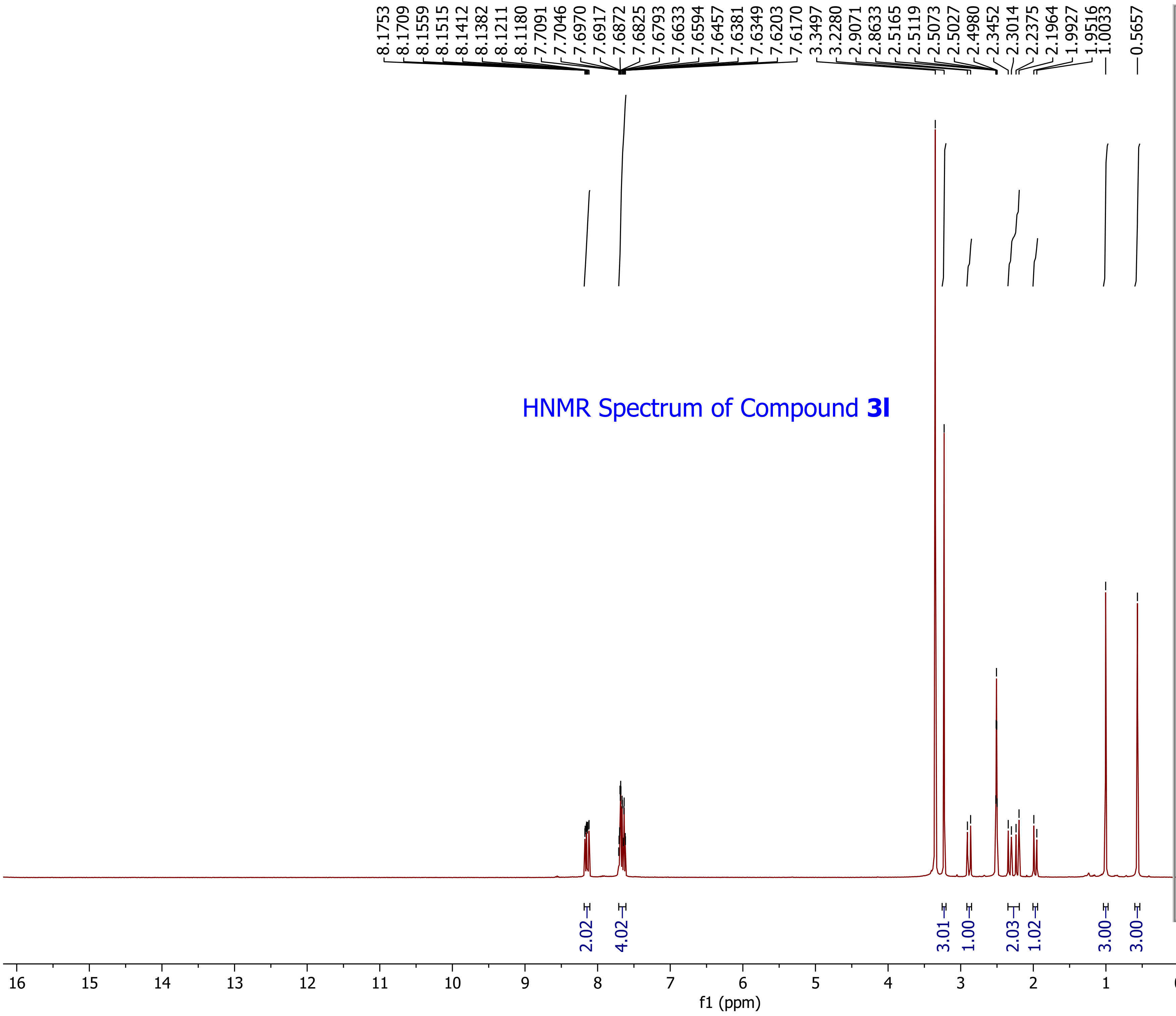

	Parameter	Value
1	Title	190201-Sample 17-HNMR.10.fid
2	Comment	190201-Sample 17-HNMR
3	Origin	Bruker BioSpin GmbH
4	Owner	nmr
5	Instrument	spect
6	Author	
7	Solvent	DMSO
8	Temperature	296.7
9	Pulse Sequence	zg30
10	Experiment	1D
11	Probe	Z108618_0166 (PA BBO 400S1 BBF-H-D-05 Z PLUS)
12	Number of Scans	16
13	Receiver Gain	161.0
14	Relaxation Delay	1.0000
15	Pulse Width	14.0000
16	Presaturation Frequency	
17	Acquisition Time	4.0894
18	Acquisition Date	2019-02-01T09:00:24
19	Modification Date	2019-02-01T14:30:26
20	Class	
21	Spectrometer Frequency	400.13
22	Spectral Width	8012.8
23	Lowest Frequency	-1535.6
24	Nucleus	1H
25	Acquired Size	32768
26	Spectral Size	65536

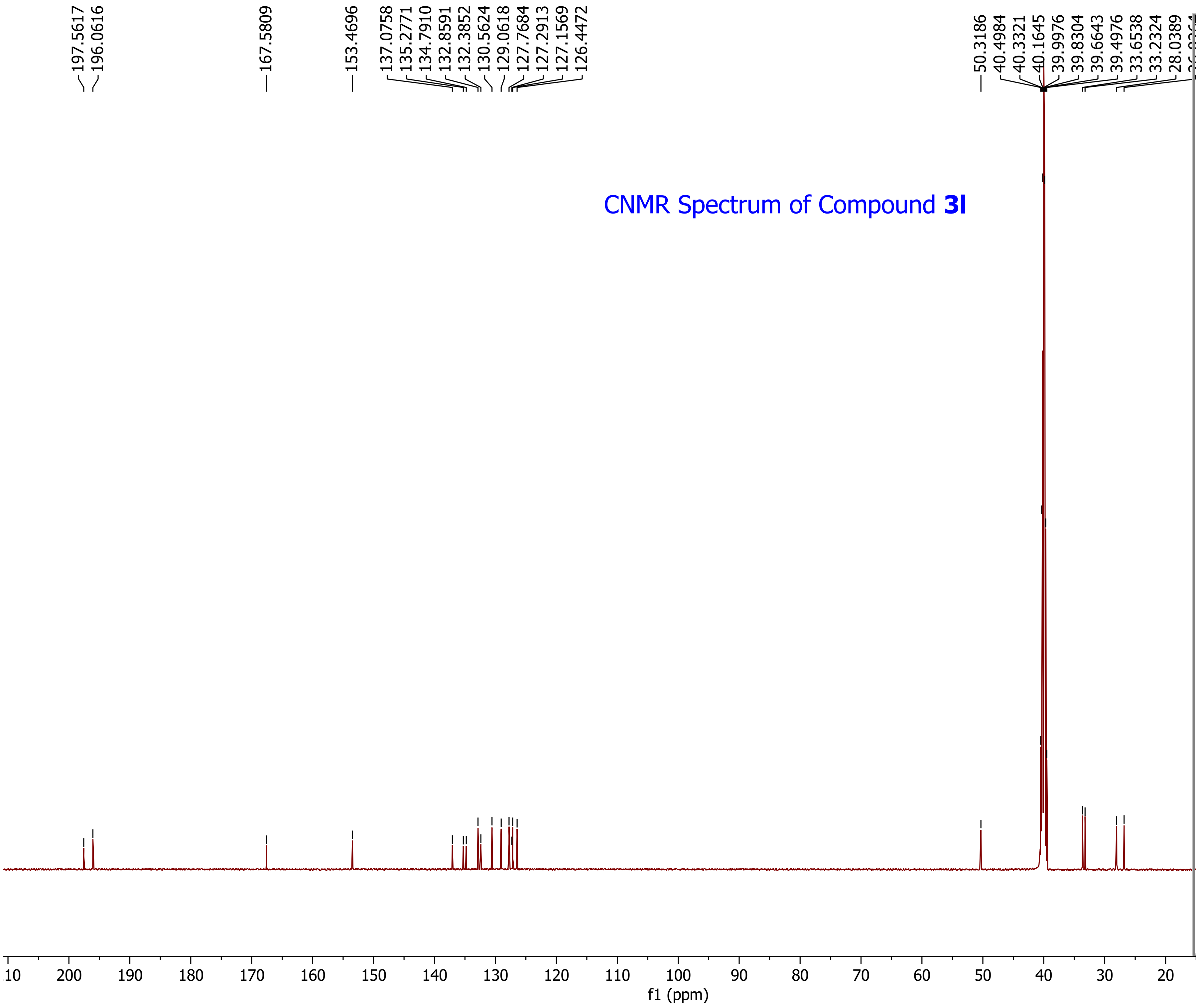

	Parameter	Value
1	Title	190216-Sample 17-CNMR.10.fid
2	Comment	190216-Sample 17-CNMR
3	Origin	Bruker BioSpin GmbH
4	Owner	nmr
5	Instrument	spect
6	Author	
7	Solvent	DMSO
8	Temperature	300.7
9	Pulse Sequence	zgpg30
10	Experiment	1D
11	Probe	Z114229_0007 (PA BBO 500S2 BBF-H-D-05 Z BTO PLUS)
12	Number of Scans	4546
13	Receiver Gain	203.0
14	Relaxation Delay	2.0000
15	Pulse Width	10.0000
16	Presaturation Frequency	
17	Acquisition Time	1.1010
18	Acquisition Date	2019-02-16T16:21:23
19	Modification Date	2019-02-16T21:51:26
20	Class	
21	Spectrometer Frequency	125.77
22	Spectral Width	29761.9
23	Lowest Frequency	-2305.6
24	Nucleus	13C
25	Acquired Size	32768
26	Spectral Size	65536

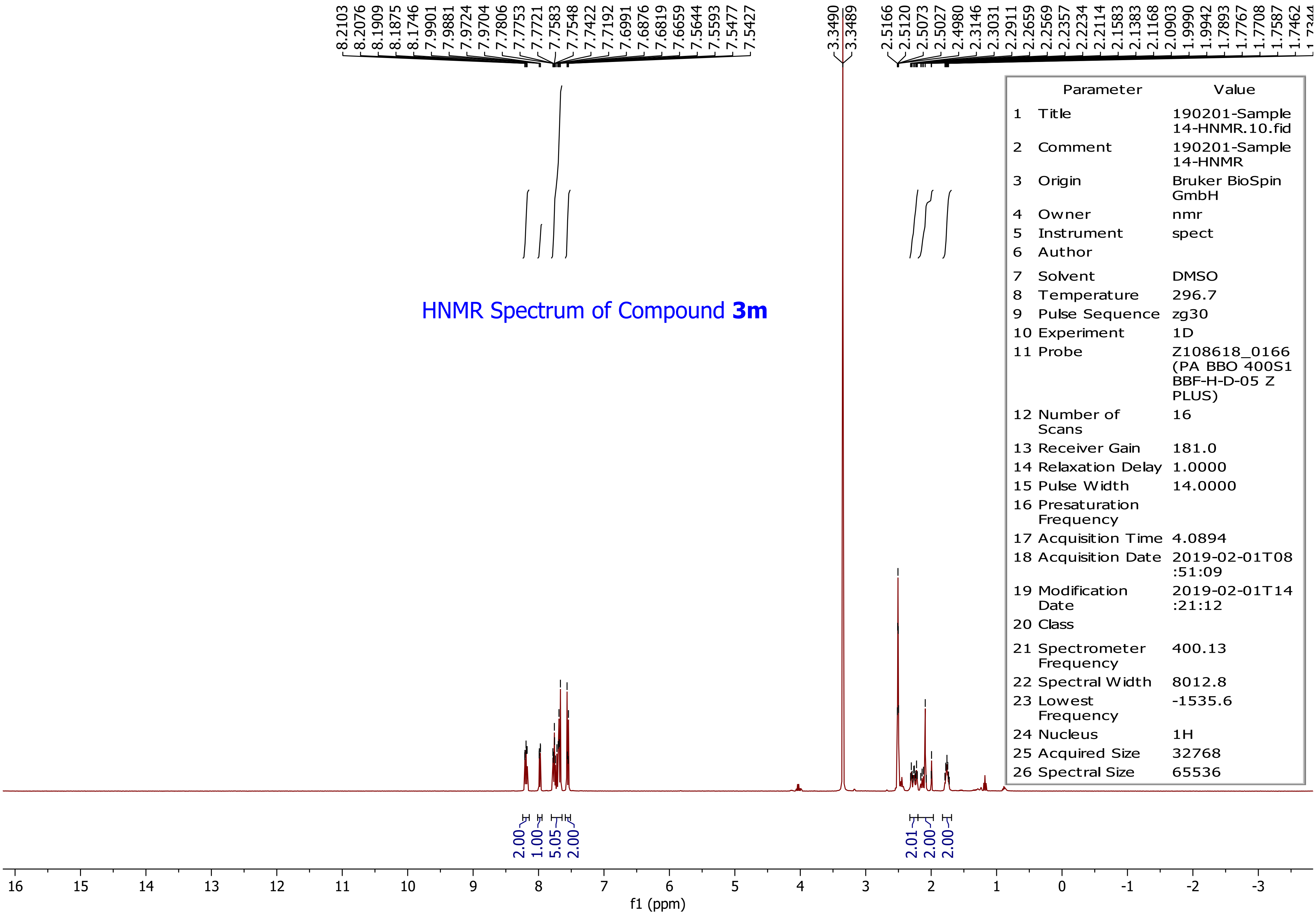

Parameter		Value
1	Title	190201-Sample 14-HNMR.10.fid
2	Comment	190201-Sample 14-HNMR
3	Origin	Bruker BioSpin GmbH
4	Owner	nmr
5	Instrument	spect
6	Author	
7	Solvent	DMSO
8	Temperature	296.7
9	Pulse Sequence	zg30
10	Experiment	1D
11	Probe	Z108618_0166 (PA BBO 400S1 BBF-H-D-05 Z PLUS)
12	Number of Scans	16
13	Receiver Gain	181.0
14	Relaxation Delay	1.0000
15	Pulse Width	14.0000
16	Presaturation Frequency	
17	Acquisition Time	4.0894
18	Acquisition Date	2019-02-01T08:51:09
19	Modification Date	2019-02-01T14:21:12
20	Class	
21	Spectrometer Frequency	400.13
22	Spectral Width	8012.8
23	Lowest Frequency	-1535.6
24	Nucleus	1H
25	Acquired Size	32768
26	Spectral Size	65536

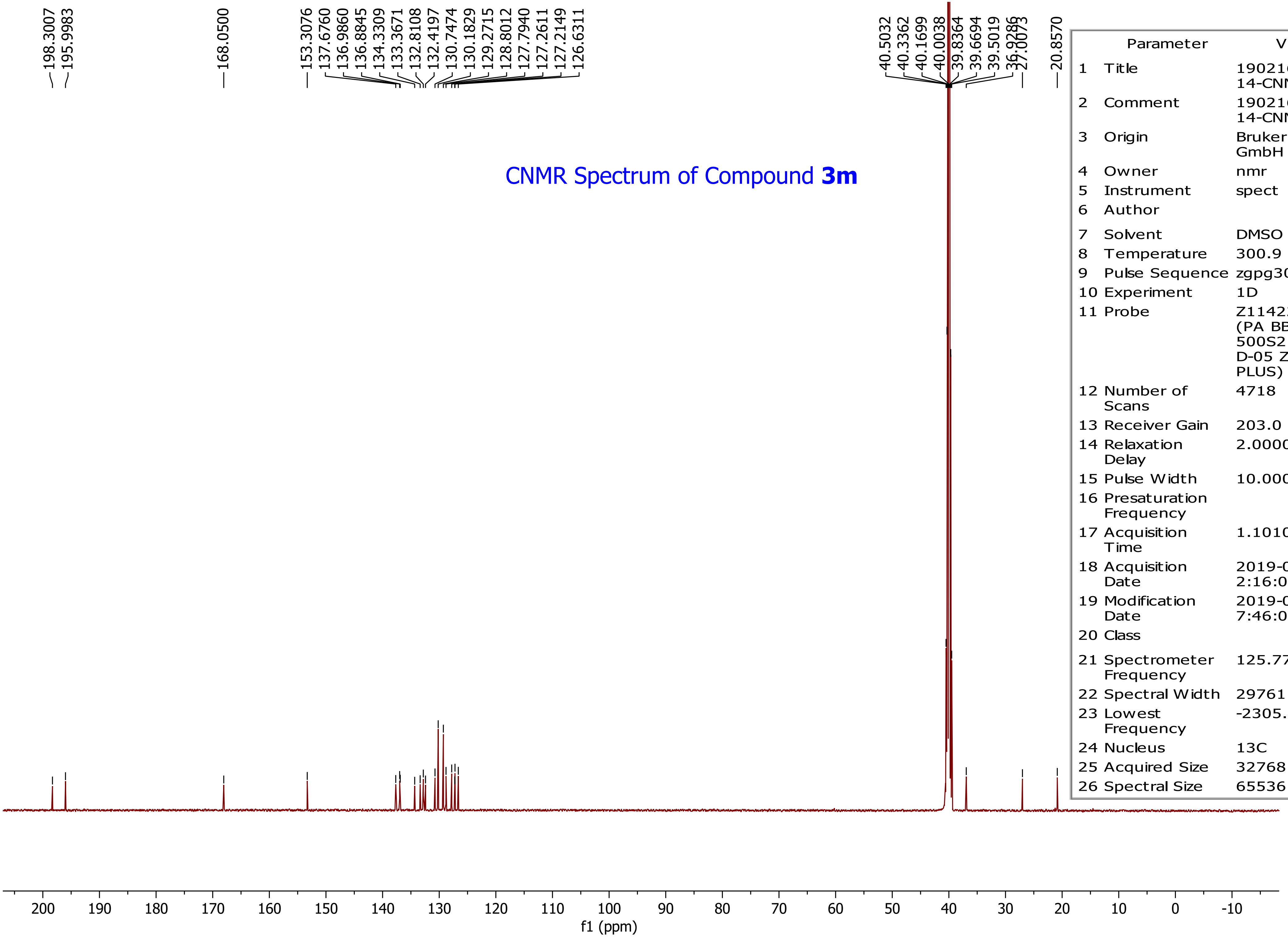

	Parameter	Value
1	Title	190216-Sample 14-CNMR.10.fid
2	Comment	190216-Sample 14-CNMR
3	Origin	Bruker BioSpin GmbH
4	Owner	nmr
5	Instrument	spect
6	Author	
7	Solvent	DMSO
8	Temperature	300.9
9	Pulse Sequence	zgpg30
10	Experiment	1D
11	Probe	Z114229_0007 (PA BBO 500S2 BBF-H-D-05 Z BTO PLUS)
12	Number of Scans	4718
13	Receiver Gain	203.0
14	Relaxation Delay	2.0000
15	Pulse Width	10.0000
16	Presaturation Frequency	
17	Acquisition Time	1.1010
18	Acquisition Date	2019-02-16T12:16:04
19	Modification Date	2019-02-16T17:46:06
20	Class	
21	Spectrometer Frequency	125.77
22	Spectral Width	29761.9
23	Lowest Frequency	-2305.6
24	Nucleus	13C
25	Acquired Size	32768
26	Spectral Size	65536

HNMR Spectrum of Compound **3n**

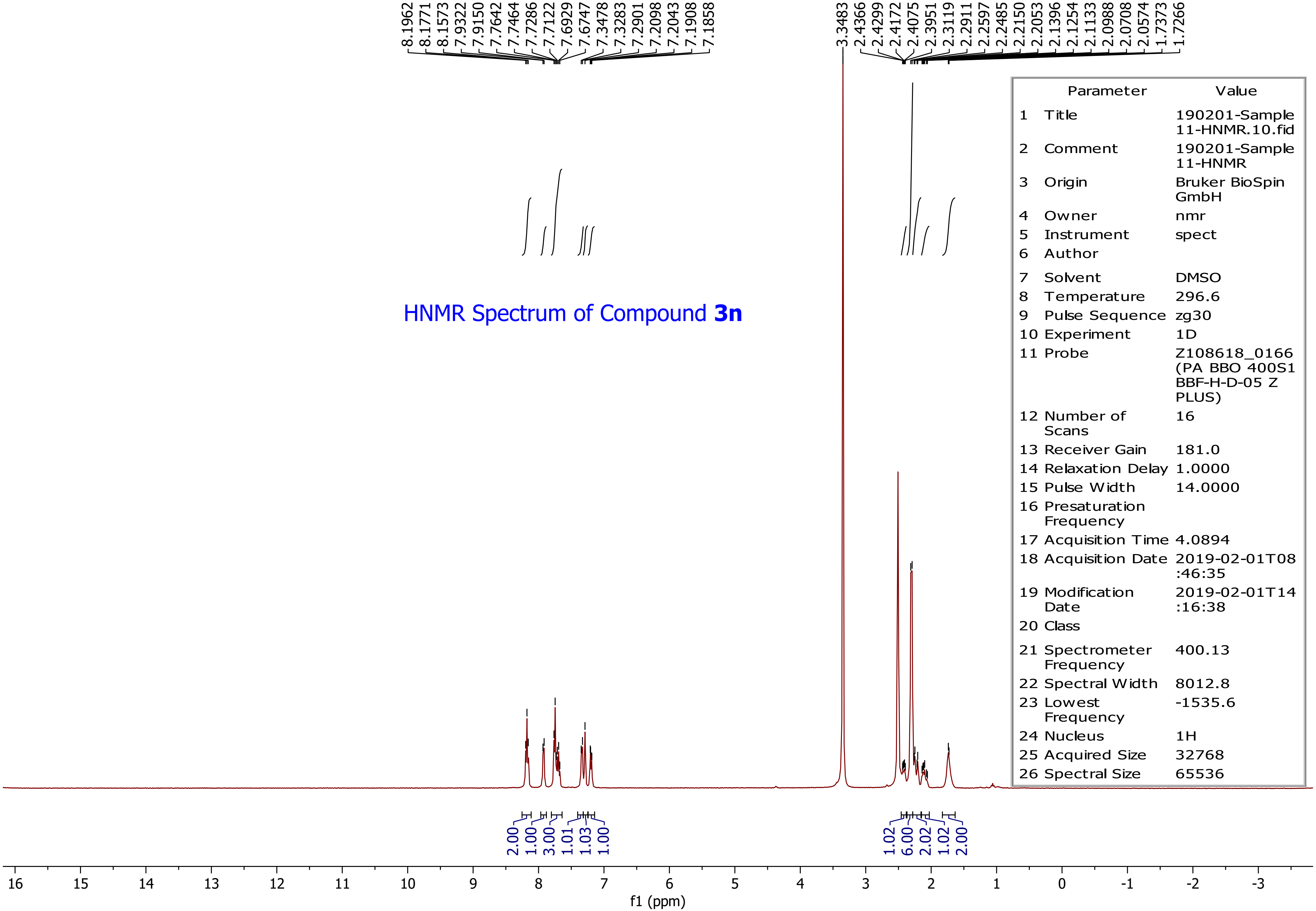

	Parameter	Value
1	Title	190201-Sample 11-HNMR.10.fid
2	Comment	190201-Sample 11-HNMR
3	Origin	Bruker BioSpin GmbH
4	Owner	nmr
5	Instrument	spect
6	Author	
7	Solvent	DMSO
8	Temperature	296.6
9	Pulse Sequence	zg30
10	Experiment	1D
11	Probe	Z108618_0166 (PA BBO 400S1 BBF-H-D-05 Z PLUS)
12	Number of Scans	16
13	Receiver Gain	181.0
14	Relaxation Delay	1.0000
15	Pulse Width	14.0000
16	Presaturation Frequency	
17	Acquisition Time	4.0894
18	Acquisition Date	2019-02-01T08:46:35
19	Modification Date	2019-02-01T14:16:38
20	Class	
21	Spectrometer Frequency	400.13
22	Spectral Width	8012.8
23	Lowest Frequency	-1535.6
24	Nucleus	1H
25	Acquired Size	32768
26	Spectral Size	65536

CNMR Spectrum of Compound **3n**

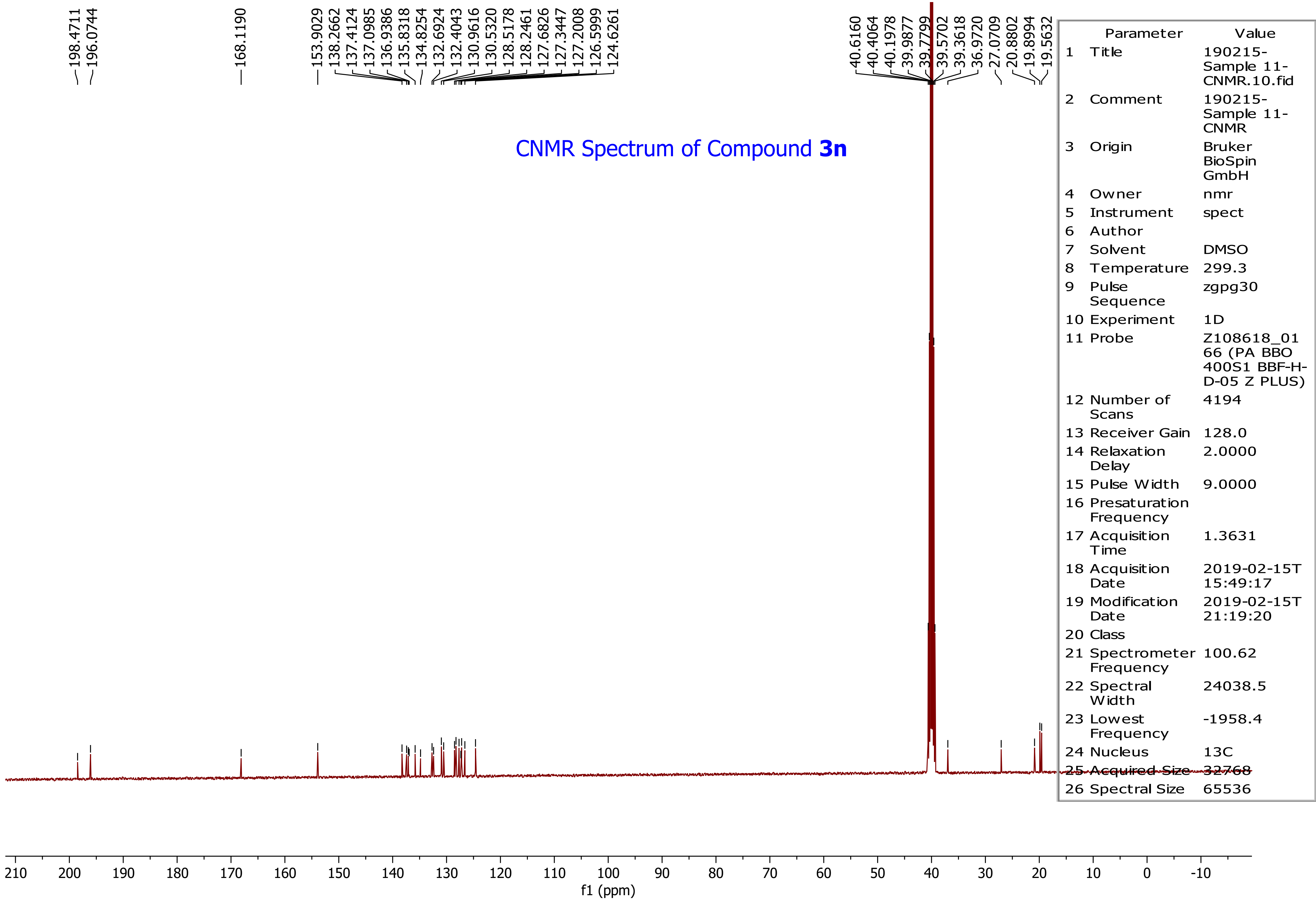

	Parameter	Value
1	Title	190215-Sample 11-CNMR.10.fid
2	Comment	190215-Sample 11-CNMR
3	Origin	Bruker BioSpin GmbH
4	Owner	nmr
5	Instrument	spect
6	Author	
7	Solvent	DMSO
8	Temperature	299.3
9	Pulse Sequence	zgpg30
10	Experiment	1D
11	Probe	Z108618_0166 (PA BBO 400S1 BBF-H-D-05 Z PLUS)
12	Number of Scans	4194
13	Receiver Gain	128.0
14	Relaxation Delay	2.0000
15	Pulse Width	9.0000
16	Presaturation Frequency	
17	Acquisition Time	1.3631
18	Acquisition Date	2019-02-15T15:49:17
19	Modification Date	2019-02-15T21:19:20
20	Class	
21	Spectrometer Frequency	100.62
22	Spectral Width	24038.5
23	Lowest Frequency	-1958.4
24	Nucleus	13C
25	Acquired Size	32768
26	Spectral Size	65536

HNMR Spectrum of Compound **3o**

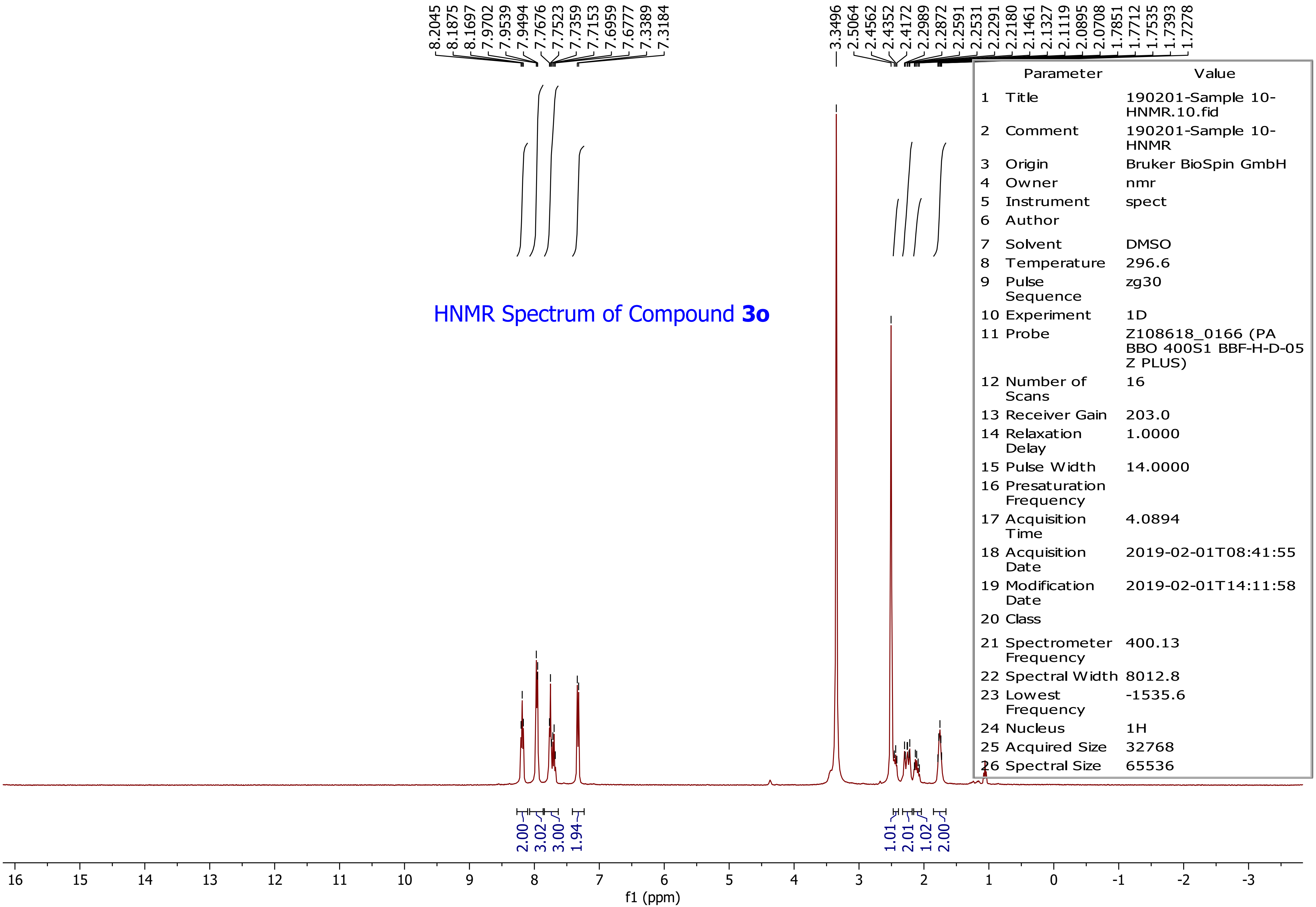

	Parameter	Value
1	Title	190201-Sample 10-HNMR.10.fid
2	Comment	190201-Sample 10-HNMR
3	Origin	Bruker BioSpin GmbH
4	Owner	nmr
5	Instrument	spect
6	Author	
7	Solvent	DMSO
8	Temperature	296.6
9	Pulse Sequence	zg30
10	Experiment	1D
11	Probe	Z108618_0166 (PA BBO 400S1 BBF-H-D-05 Z PLUS)
12	Number of Scans	16
13	Receiver Gain	203.0
14	Relaxation Delay	1.0000
15	Pulse Width	14.0000
16	Presaturation Frequency	
17	Acquisition Time	4.0894
18	Acquisition Date	2019-02-01T08:41:55
19	Modification Date	2019-02-01T14:11:58
20	Class	
21	Spectrometer Frequency	400.13
22	Spectral Width	8012.8
23	Lowest Frequency	-1535.6
24	Nucleus	1H
25	Acquired Size	32768
26	Spectral Size	65536

CNMR Spectrum of Compound **3o**

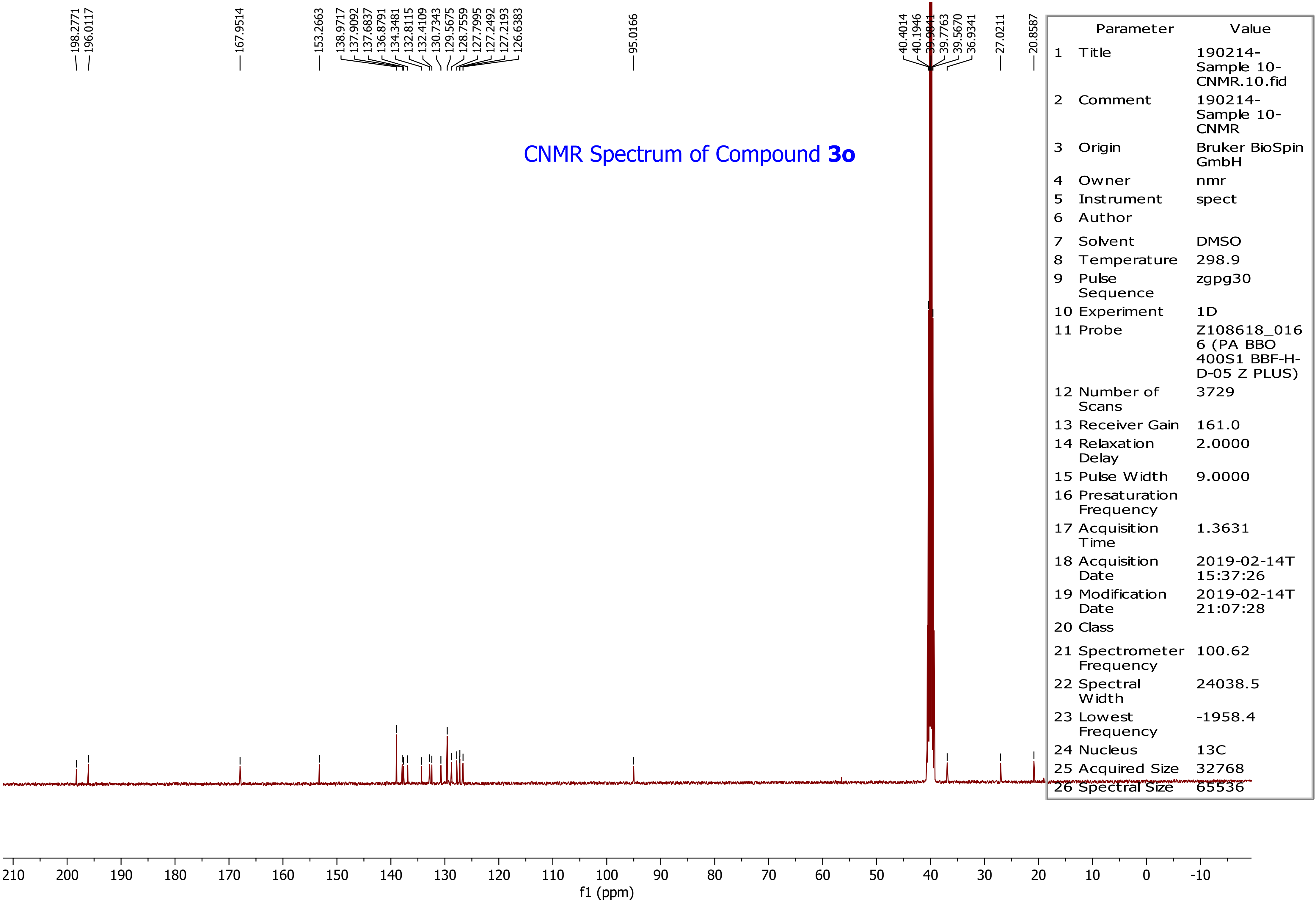

	Parameter	Value
1	Title	190214-Sample 10-CNMR.10.fid
2	Comment	190214-Sample 10-CNMR
3	Origin	Bruker BioSpin GmbH
4	Owner	nmr
5	Instrument	spect
6	Author	
7	Solvent	DMSO
8	Temperature	298.9
9	Pulse Sequence	zgpg30
10	Experiment	1D
11	Probe	Z108618_0166 (PA BBO 400S1 BBF-H-D-05 Z PLUS)
12	Number of Scans	3729
13	Receiver Gain	161.0
14	Relaxation Delay	2.0000
15	Pulse Width	9.0000
16	Presaturation Frequency	
17	Acquisition Time	1.3631
18	Acquisition Date	2019-02-14T15:37:26
19	Modification Date	2019-02-14T21:07:28
20	Class	
21	Spectrometer Frequency	100.62
22	Spectral Width	24038.5
23	Lowest Frequency	-1958.4
24	Nucleus	13C
25	Acquired Size	32768
26	Spectral Size	65536

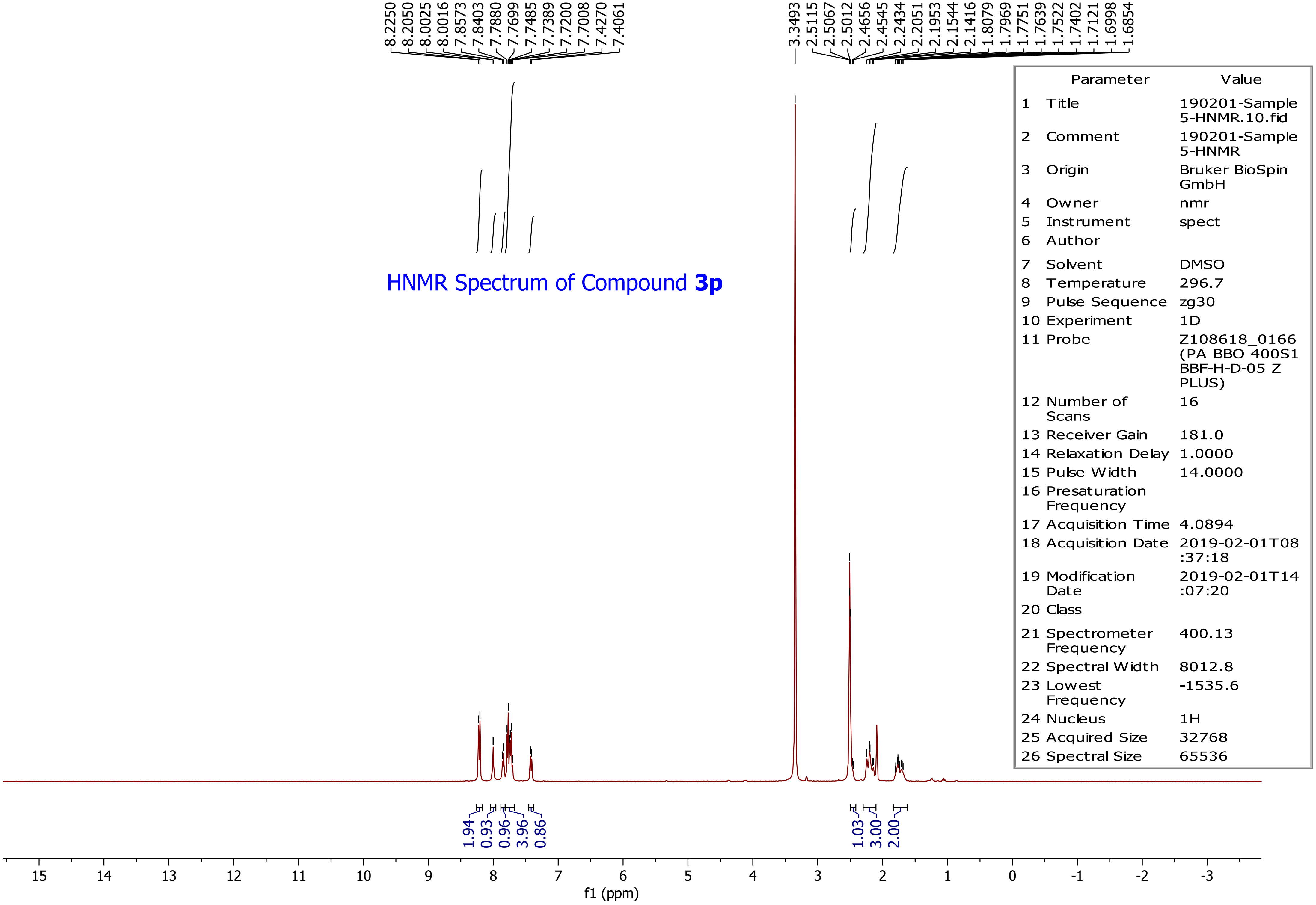

HNMR Spectrum of Compound **3p**

	Parameter	Value
1	Title	190201-Sample 5-HNMR.10.fid
2	Comment	190201-Sample 5-HNMR
3	Origin	Bruker BioSpin GmbH
4	Owner	nmr
5	Instrument	spect
6	Author	
7	Solvent	DMSO
8	Temperature	296.7
9	Pulse Sequence	zg30
10	Experiment	1D
11	Probe	Z108618_0166 (PA BBO 400S1 BBF-H-D-05 Z PLUS)
12	Number of Scans	16
13	Receiver Gain	181.0
14	Relaxation Delay	1.0000
15	Pulse Width	14.0000
16	Presaturation Frequency	
17	Acquisition Time	4.0894
18	Acquisition Date	2019-02-01T08:37:18
19	Modification Date	2019-02-01T14:07:20
20	Class	
21	Spectrometer Frequency	400.13
22	Spectral Width	8012.8
23	Lowest Frequency	-1535.6
24	Nucleus	1H
25	Acquired Size	32768
26	Spectral Size	65536

CNMR Spectrum of Compound **3p**

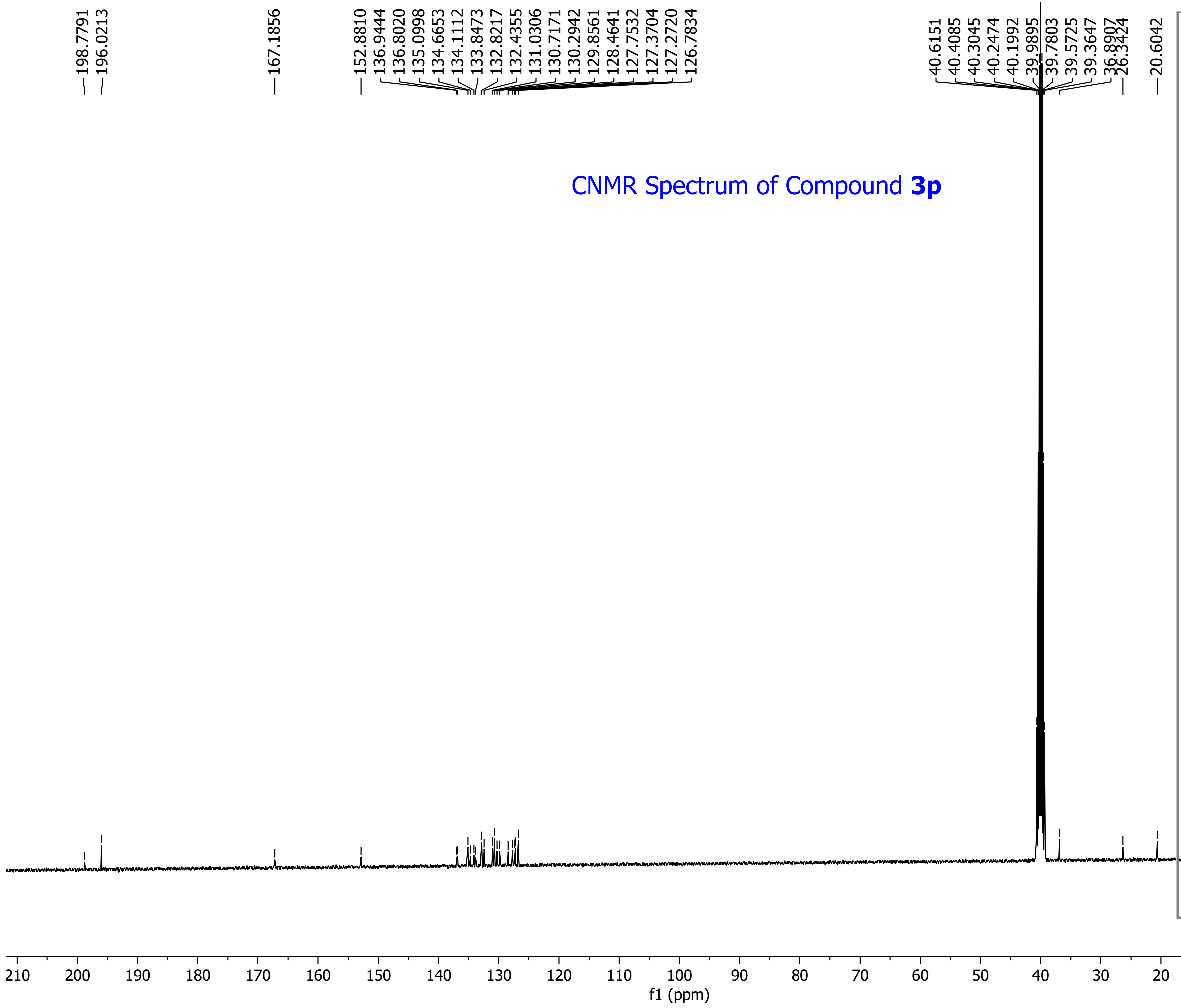

	Parameter	Value
1	Title	190212-Sample 5-CNMR.10.fid
2	Comment	190212-Sample 5-CNMR
3	Origin	Bruker BioSpin GmbH
4	Owner	nmr
5	Instrument	spect
6	Author	
7	Solvent	DMSO
8	Temperature	298.6
9	Pulse Sequence	zgpg30
10	Experiment	1D
11	Probe	Z108618_0166 (PA BBO 400S1 BBF-H-D-05 Z PLUS)
12	Number of Scans	2973
13	Receiver Gain	57.0
14	Relaxation Delay	2.0000
15	Pulse Width	9.0000
16	Presaturation Frequency	
17	Acquisition Time	1.3631
18	Acquisition Date	2019-02-12T15:03:45
19	Modification Date	2019-02-12T20:33:48
20	Class	
21	Spectrometer Frequency	100.62
22	Spectral Width	24038.5
23	Lowest Frequency	-1958.4
24	Nucleus	13C
25	Acquired Size	32768
26	Spectral Size	65536

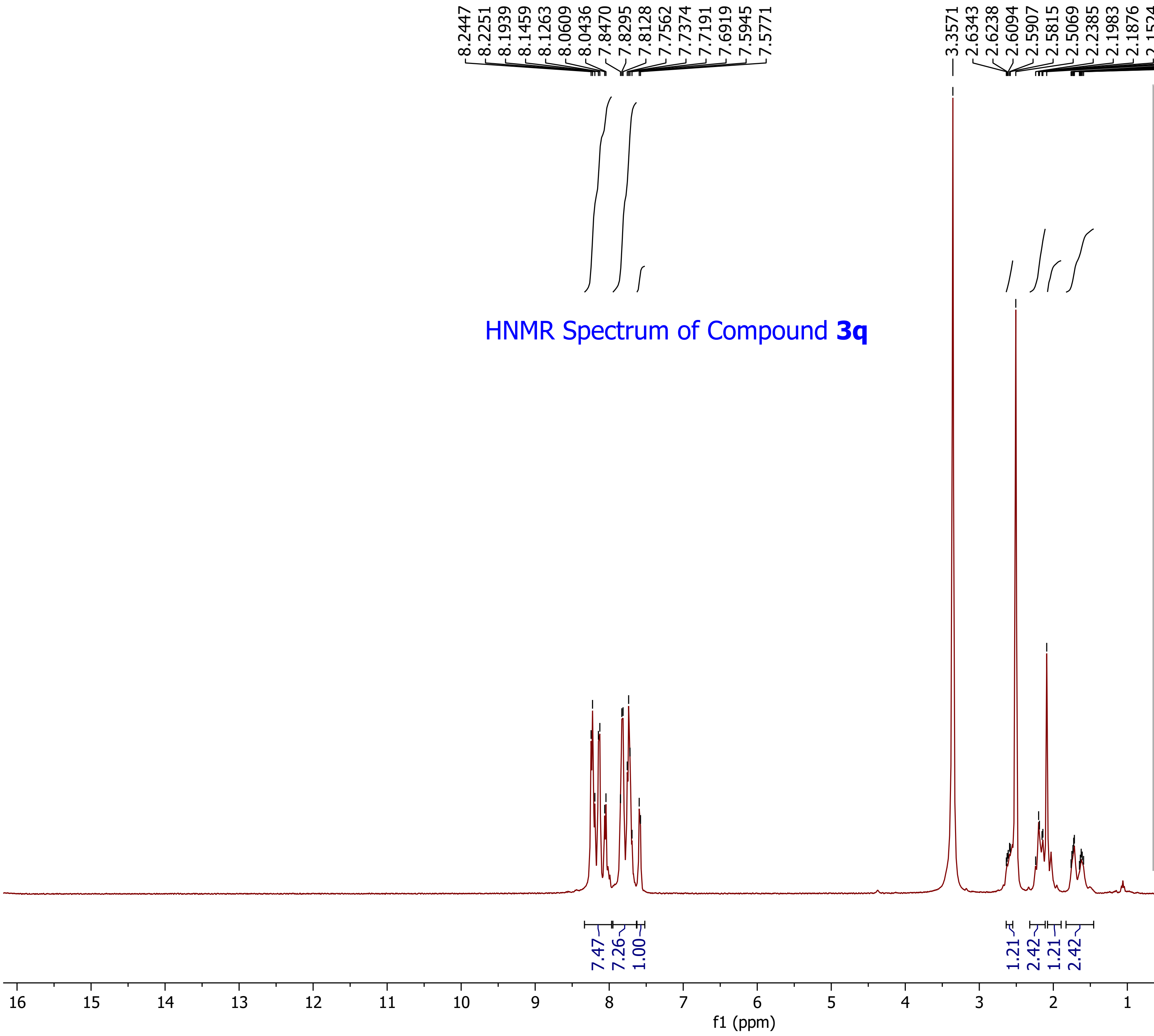

	Parameter	Value
1	Title	190201-Sample 21-HNMR.10.fid
2	Comment	190201-Sample 21-HNMR
3	Origin	Bruker BioSpin GmbH
4	Owner	nmr
5	Instrument	spect
6	Author	
7	Solvent	DMSO
8	Temperature	296.6
9	Pulse Sequence	zg30
10	Experiment	1D
11	Probe	Z108618_0166 (PA BBO 400S1 BBF-H-D-05 Z PLUS)
12	Number of Scans	16
13	Receiver Gain	161.0
14	Relaxation Delay	1.0000
15	Pulse Width	14.0000
16	Presaturation Frequency	
17	Acquisition Time	4.0894
18	Acquisition Date	2019-02-01T09:19:59
19	Modification Date	2019-02-01T14:50:00
20	Class	
21	Spectrometer Frequency	400.13
22	Spectral Width	8012.8
23	Lowest Frequency	-1535.6
24	Nucleus	1H
25	Acquired Size	32768
26	Spectral Size	65536

CNMR Spectrum of Compound **3q**

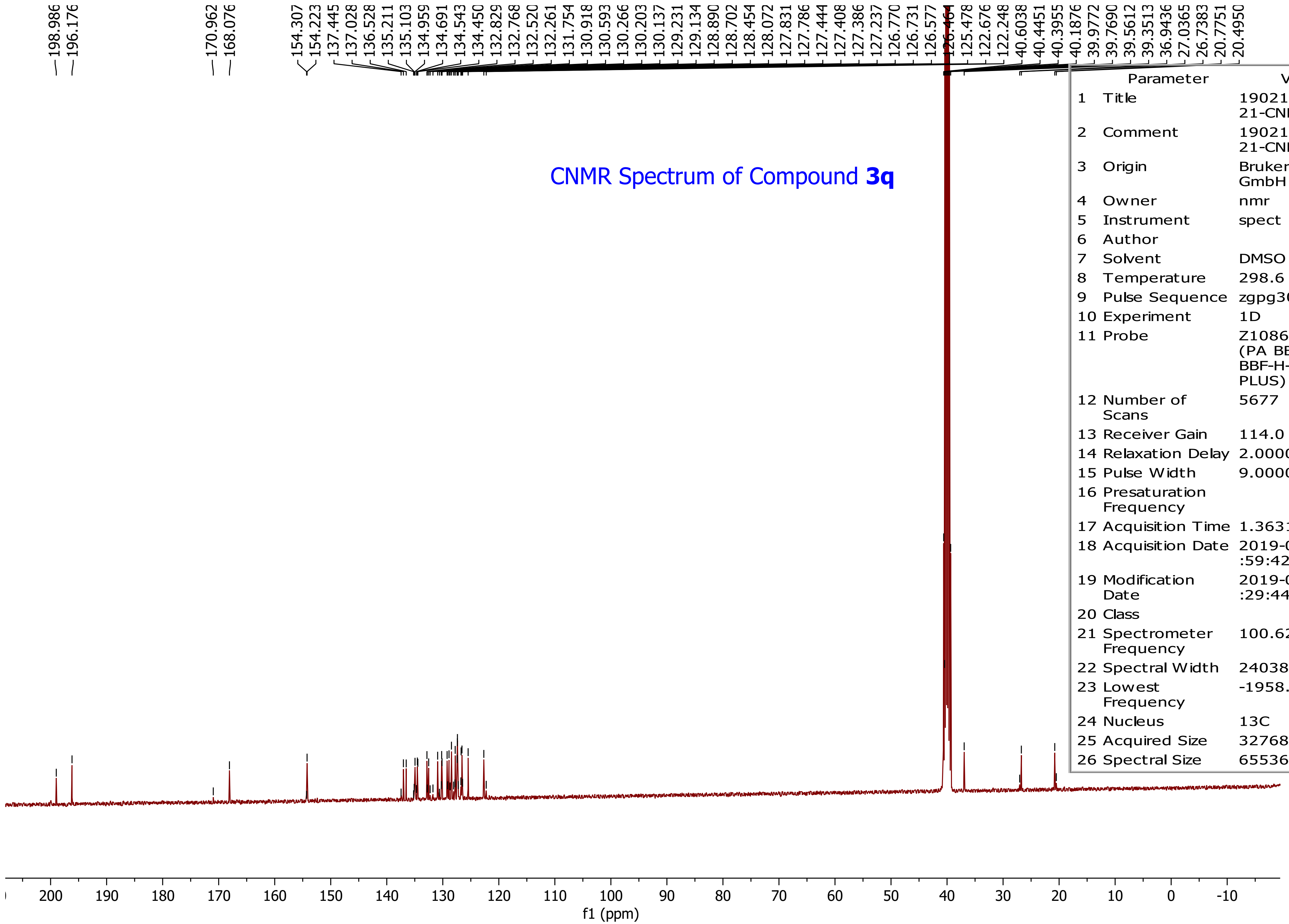

	Parameter	Value
1	Title	190217-Sample 21-CNMR.10.fid
2	Comment	190217-Sample 21-CNMR
3	Origin	Bruker BioSpin GmbH
4	Owner	nmr
5	Instrument	spect
6	Author	
7	Solvent	DMSO
8	Temperature	298.6
9	Pulse Sequence	zgpg30
10	Experiment	1D
11	Probe	Z108618_0166 (PA BBO 400S1 BBF-H-D-05 Z PLUS)
12	Number of Scans	5677
13	Receiver Gain	114.0
14	Relaxation Delay	2.0000
15	Pulse Width	9.0000
16	Presaturation Frequency	
17	Acquisition Time	1.3631
18	Acquisition Date	2019-02-17T17:59:42
19	Modification Date	2019-02-17T23:29:44
20	Class	
21	Spectrometer Frequency	100.62
22	Spectral Width	24038.5
23	Lowest Frequency	-1958.4
24	Nucleus	13C
25	Acquired Size	32768
26	Spectral Size	65536

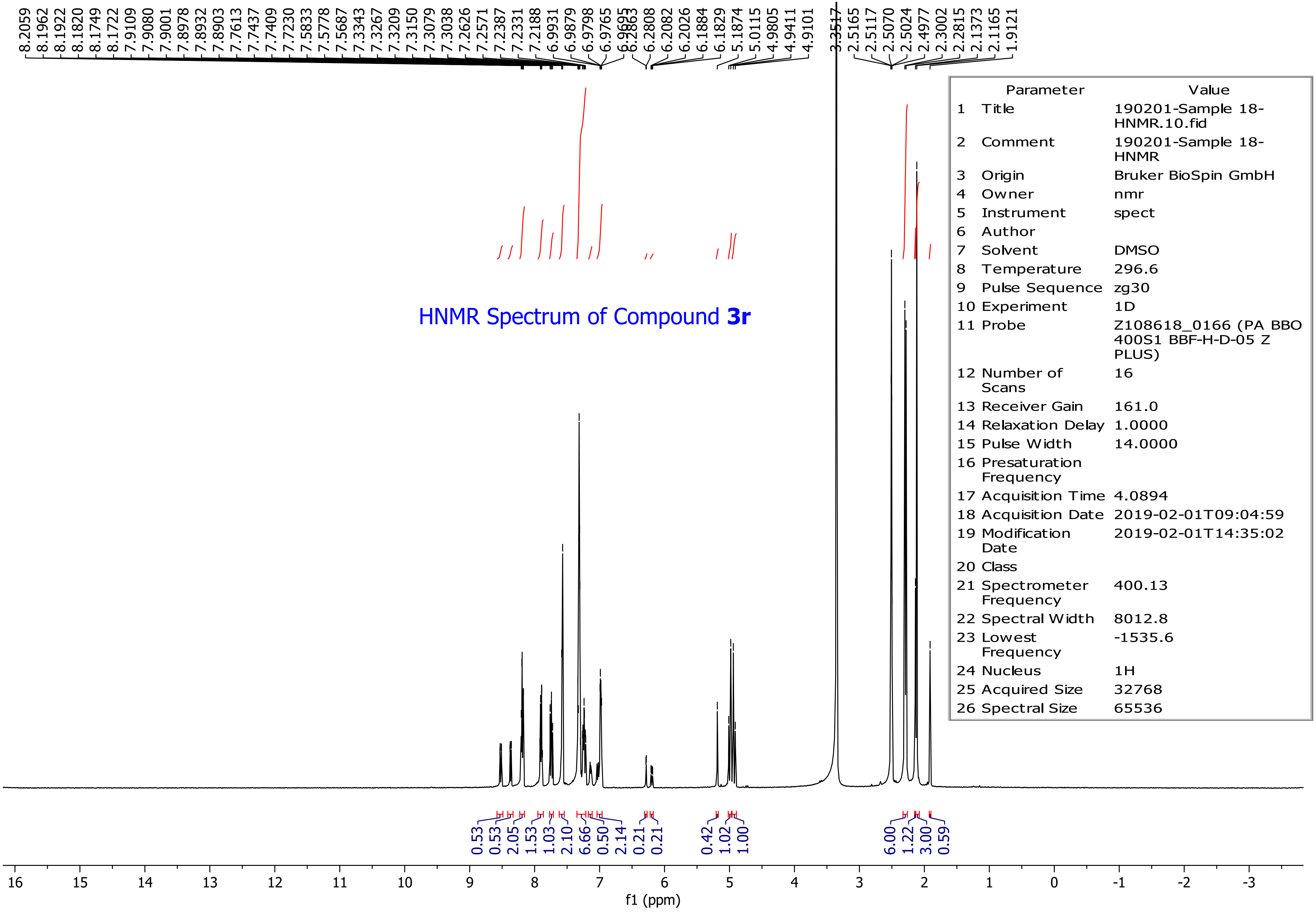

	Parameter	Value
1	Title	190201-Sample 18-HNMR.10.fid
2	Comment	190201-Sample 18-HNMR
3	Origin	Bruker BioSpin GmbH
4	Owner	nmr
5	Instrument	spect
6	Author	
7	Solvent	DMSO
8	Temperature	296.6
9	Pulse Sequence	zg30
10	Experiment	1D
11	Probe	Z108618_0166 (PA BBO 400S1 BBF-H-D-05 Z PLUS)
12	Number of Scans	16
13	Receiver Gain	161.0
14	Relaxation Delay	1.0000
15	Pulse Width	14.0000
16	Presaturation Frequency	
17	Acquisition Time	4.0894
18	Acquisition Date	2019-02-01T09:04:59
19	Modification Date	2019-02-01T14:35:02
20	Class	
21	Spectrometer Frequency	400.13
22	Spectral Width	8012.8
23	Lowest Frequency	-1535.6
24	Nucleus	1H
25	Acquired Size	32768
26	Spectral Size	65536

HNMR Spectrum of Compound **3r** at 80 ^{o}C

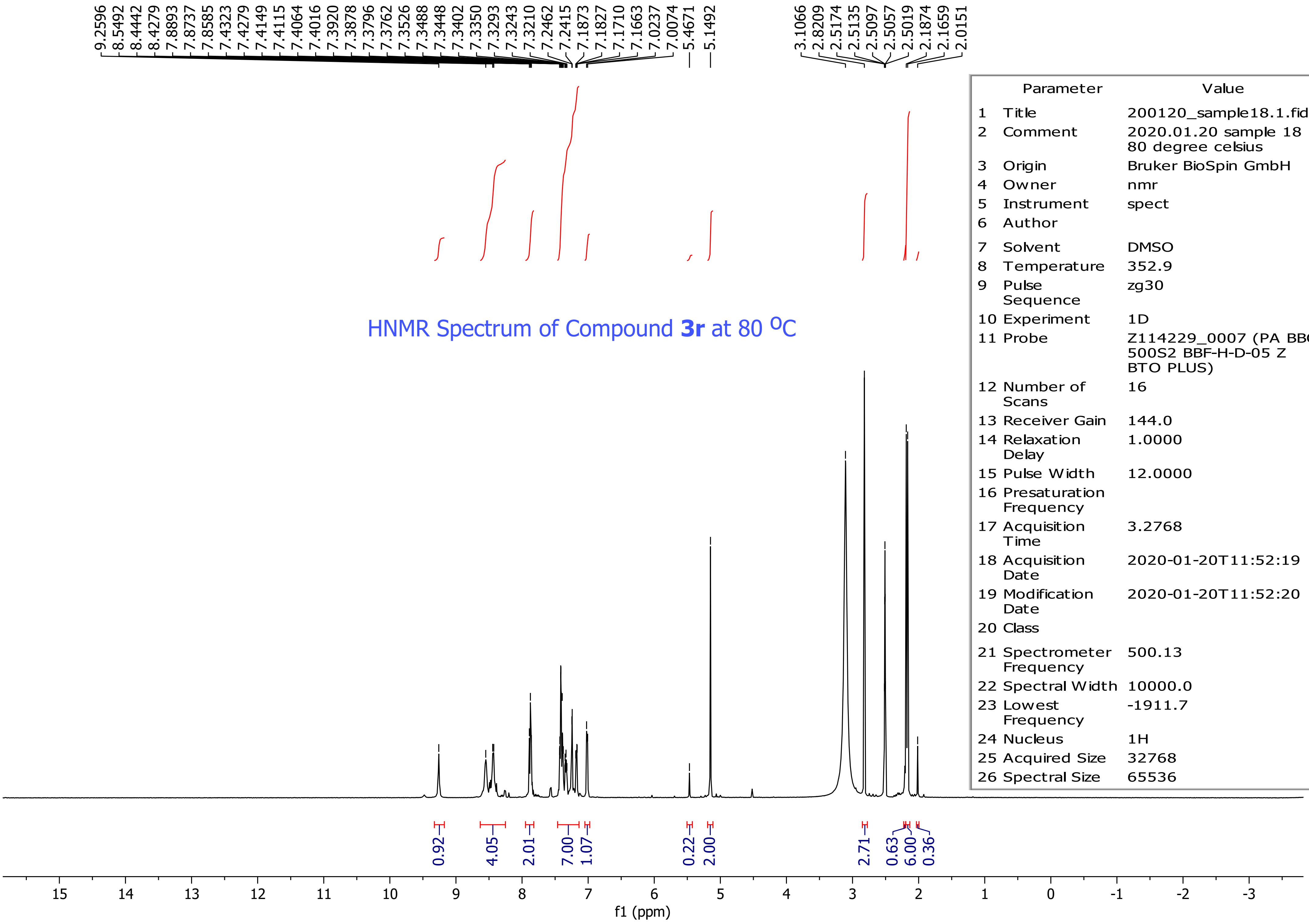

	Parameter	Value
1	Title	200120_sample18.1.fid
2	Comment	2020.01.20 sample 18 80 degree celsius
3	Origin	Bruker BioSpin GmbH
4	Owner	nmr
5	Instrument	spect
6	Author	
7	Solvent	DMSO
8	Temperature	352.9
9	Pulse Sequence	zg30
10	Experiment	1D
11	Probe	Z114229_0007 (PA BBO 500S2 BBF-H-D-05 Z BTO PLUS)
12	Number of Scans	16
13	Receiver Gain	144.0
14	Relaxation Delay	1.0000
15	Pulse Width	12.0000
16	Presaturation Frequency	
17	Acquisition Time	3.2768
18	Acquisition Date	2020-01-20T11:52:19
19	Modification Date	2020-01-20T11:52:20
20	Class	
21	Spectrometer Frequency	500.13
22	Spectral Width	10000.0
23	Lowest Frequency	-1911.7
24	Nucleus	1H
25	Acquired Size	32768
26	Spectral Size	65536

CNMR Spectrum of Compound **3r**

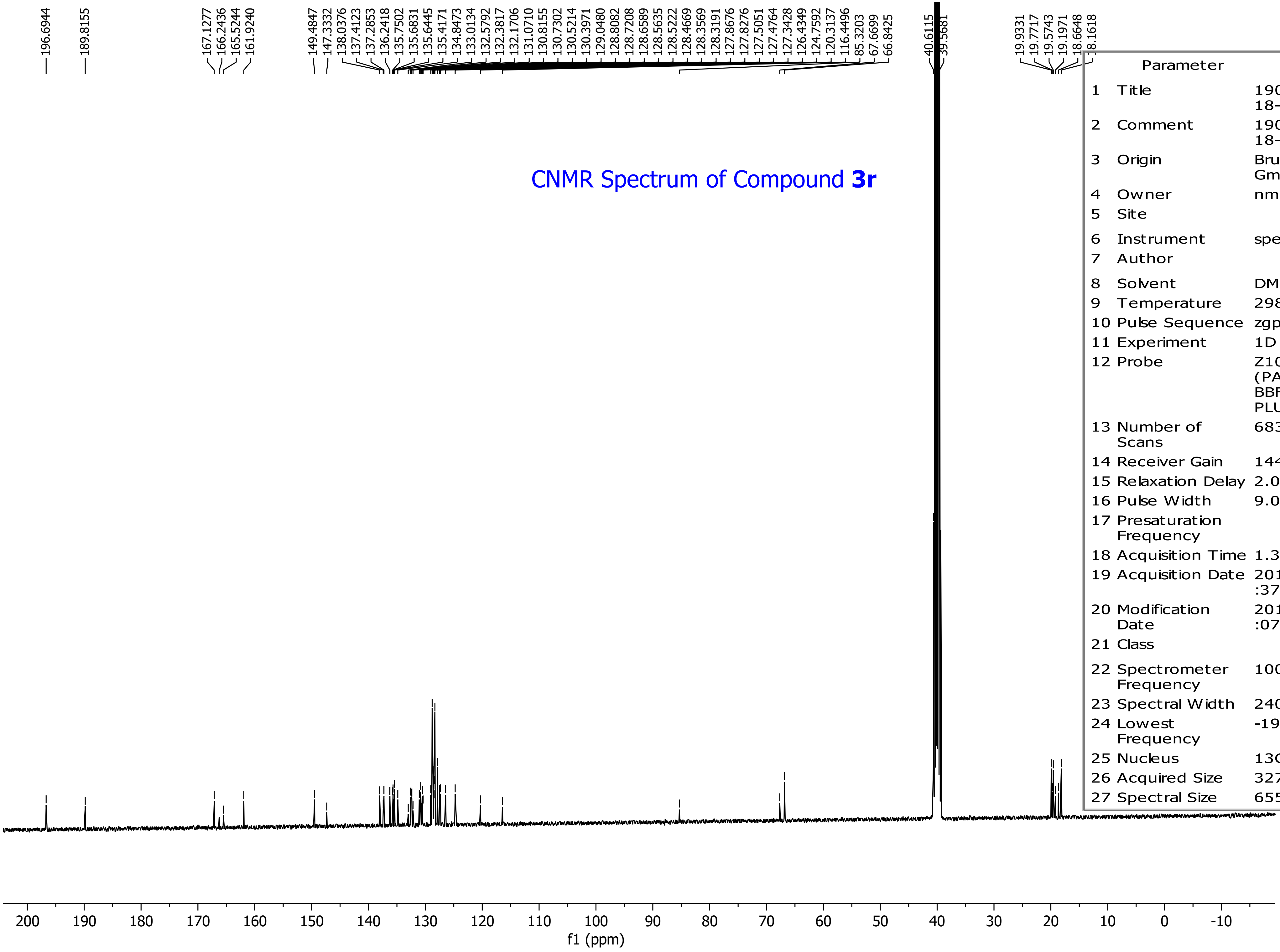

	Parameter	Value
1	Title	190323-Sample 18-CNMR.10.fid
2	Comment	190323-Sample 18-CNMR
3	Origin	Bruker BioSpin GmbH
4	Owner	nmr
5	Site	
6	Instrument	spect
7	Author	
8	Solvent	DMSO
9	Temperature	298.9
10	Pulse Sequence	zgpg30
11	Experiment	1D
12	Probe	Z108618_0166 (PA BBO 400S1 BBF-H-D-05 Z PLUS)
13	Number of Scans	6831
14	Receiver Gain	144.0
15	Relaxation Delay	2.0000
16	Pulse Width	9.0000
17	Presaturation Frequency	
18	Acquisition Time	1.3631
19	Acquisition Date	2019-03-23T18:37:51
20	Modification Date	2019-03-23T22:07:54
21	Class	
22	Spectrometer Frequency	100.62
23	Spectral Width	24038.5
24	Lowest Frequency	-1958.4
25	Nucleus	13C
26	Acquired Size	32768
27	Spectral Size	65536

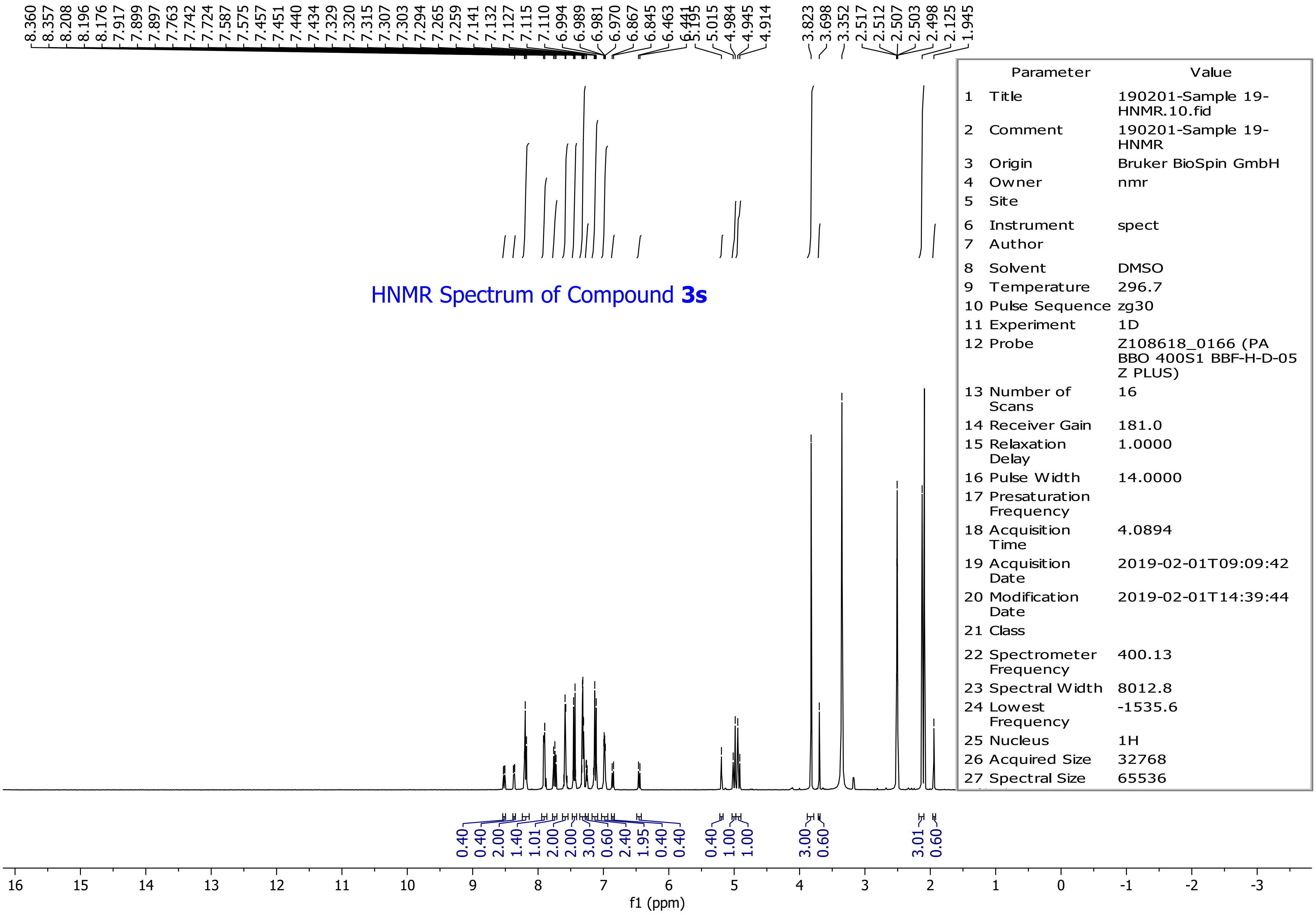

HNMR Spectrum of Compound **3s**

	Parameter	Value
1	Title	190201-Sample 19-HNMR.10.fid
2	Comment	190201-Sample 19-HNMR
3	Origin	Bruker BioSpin GmbH
4	Owner	nmr
5	Site	
6	Instrument	spect
7	Author	
8	Solvent	DMSO
9	Temperature	296.7
10	Pulse Sequence	zg30
11	Experiment	1D
12	Probe	Z108618_0166 (PA BBO 400S1 BBF-H-D-05 Z PLUS)
13	Number of Scans	16
14	Receiver Gain	181.0
15	Relaxation Delay	1.0000
16	Pulse Width	14.0000
17	Presaturation Frequency	
18	Acquisition Time	4.0894
19	Acquisition Date	2019-02-01T09:09:42
20	Modification Date	2019-02-01T14:39:44
21	Class	
22	Spectrometer Frequency	400.13
23	Spectral Width	8012.8
24	Lowest Frequency	-1535.6
25	Nucleus	1H
26	Acquired Size	32768
27	Spectral Size	65536

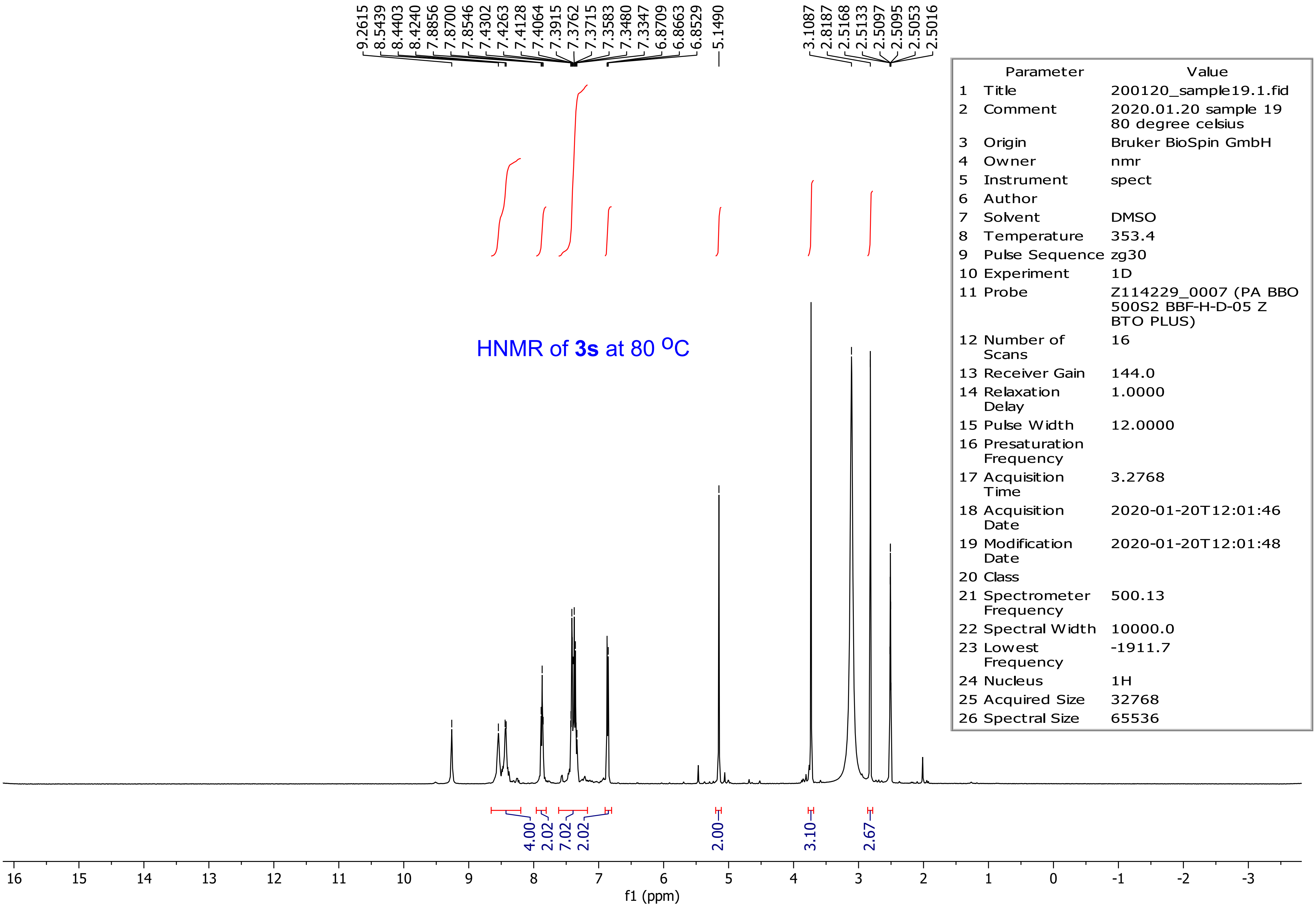

	Parameter	Value
1	Title	200120_sample19.1.fid
2	Comment	2020.01.20 sample 19 80 degree celsius
3	Origin	Bruker BioSpin GmbH
4	Owner	nmr
5	Instrument	spect
6	Author	
7	Solvent	DMSO
8	Temperature	353.4
9	Pulse Sequence	zg30
10	Experiment	1D
11	Probe	Z114229_0007 (PA BBO 500S2 BBF-H-D-05 Z BTO PLUS)
12	Number of Scans	16
13	Receiver Gain	144.0
14	Relaxation Delay	1.0000
15	Pulse Width	12.0000
16	Presaturation Frequency	
17	Acquisition Time	3.2768
18	Acquisition Date	2020-01-20T12:01:46
19	Modification Date	2020-01-20T12:01:48
20	Class	
21	Spectrometer Frequency	500.13
22	Spectral Width	10000.0
23	Lowest Frequency	-1911.7
24	Nucleus	1H
25	Acquired Size	32768
26	Spectral Size	65536

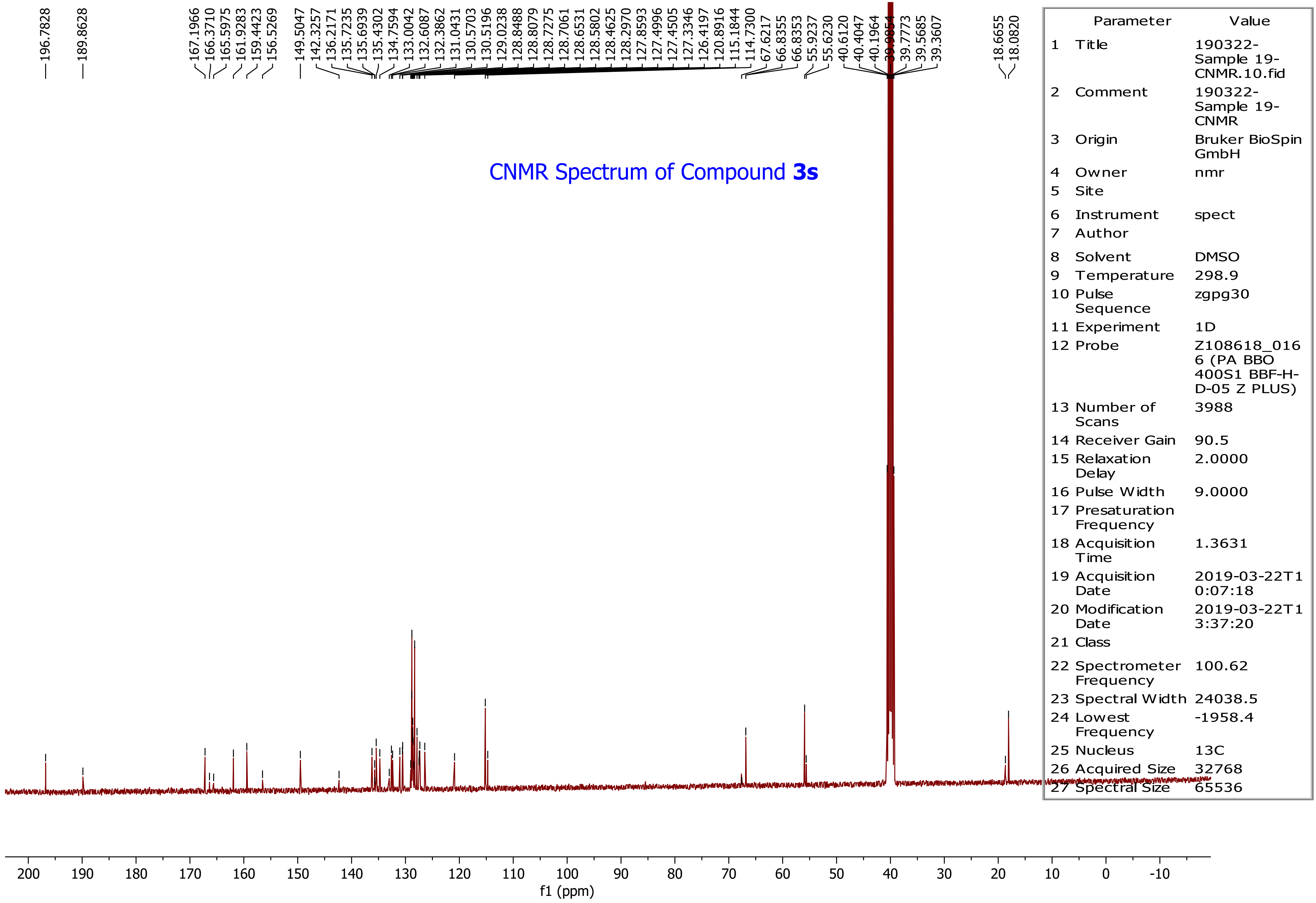

CNMR Spectrum of Compound **3s**
196.7828
189.8628
167.1966
166.3710
165.5975
161.9283
159.4423
156.5269
149.5047
142.3257
136.2171
135.7235
135.6939
135.4302
134.7594
133.0042
132.6087
132.3862
131.0431
130.5703
130.5196
129.0238
128.8488
128.8079
128.7275
128.7061
128.6531
128.5802
128.4625
128.2970
127.8593
127.4996
127.4505
127.3346
126.4197
120.8916
115.1844
114.7300
67.6217
66.8355
66.8353
55.9237
55.6230
40.6120
40.4047
40.1964
39.9854
39.7773
39.5685
39.3607
18.6655
18.0820
Parameter
Value
1 Title 190322-Sample 19-CNMR.10.fid
2 Comment 190322-Sample 19-CNMR
3 Origin Bruker BioSpin GmbH
4 Owner nmr
5 Site
6 Instrument spect
7 Author
8 Solvent DMSO
9 Temperature 298.9
10 Pulse Sequence zgpg30
11 Experiment 1D
12 Probe Z108618_0166 (PA BBO 400S1 BBF-H-D-05 Z PLUS)
13 Number of Scans 3988
14 Receiver Gain 90.5
15 Relaxation Delay 2.0000
16 Pulse Width 9.0000
17 Presaturation Frequency
18 Acquisition Time 1.3631
19 Acquisition Date 2019-03-22T10:07:18
20 Modification Date 2019-03-22T13:37:20
21 Class
22 Spectrometer Frequency 100.62
23 Spectral Width 24038.5
24 Lowest Frequency -1958.4
25 Nucleus 13C
26 Acquired Size 32768
27 Spectral Size 65536
200
190
180
170
160
150
140
130
120
110
100
90
80
70
60
50
40
30
20
10
0
-10
f1 (ppm)

190201-Sample 20-HNMR.10.fid — 190201-Sample 20-HNMR

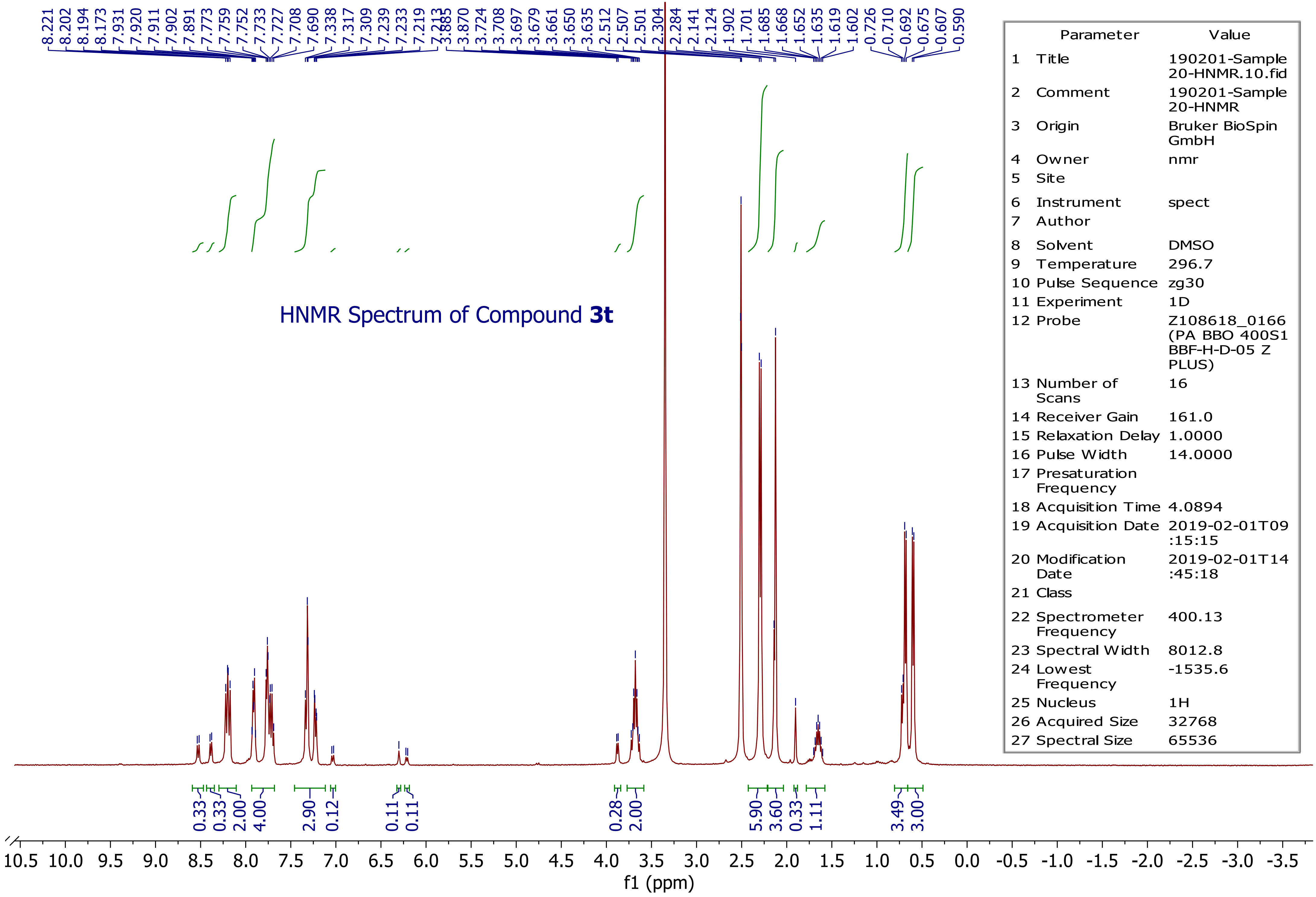

	Parameter	Value
1	Title	190201-Sample 20-HNMR.10.fid
2	Comment	190201-Sample 20-HNMR
3	Origin	Bruker BioSpin GmbH
4	Owner	nmr
5	Site	
6	Instrument	spect
7	Author	
8	Solvent	DMSO
9	Temperature	296.7
10	Pulse Sequence	zg30
11	Experiment	1D
12	Probe	Z108618_0166 (PA BBO 400S1 BBF-H-D-05 Z PLUS)
13	Number of Scans	16
14	Receiver Gain	161.0
15	Relaxation Delay	1.0000
16	Pulse Width	14.0000
17	Presaturation Frequency	
18	Acquisition Time	4.0894
19	Acquisition Date	2019-02-01T09:15:15
20	Modification Date	2019-02-01T14:45:18
21	Class	
22	Spectrometer Frequency	400.13
23	Spectral Width	8012.8
24	Lowest Frequency	-1535.6
25	Nucleus	1H
26	Acquired Size	32768
27	Spectral Size	65536

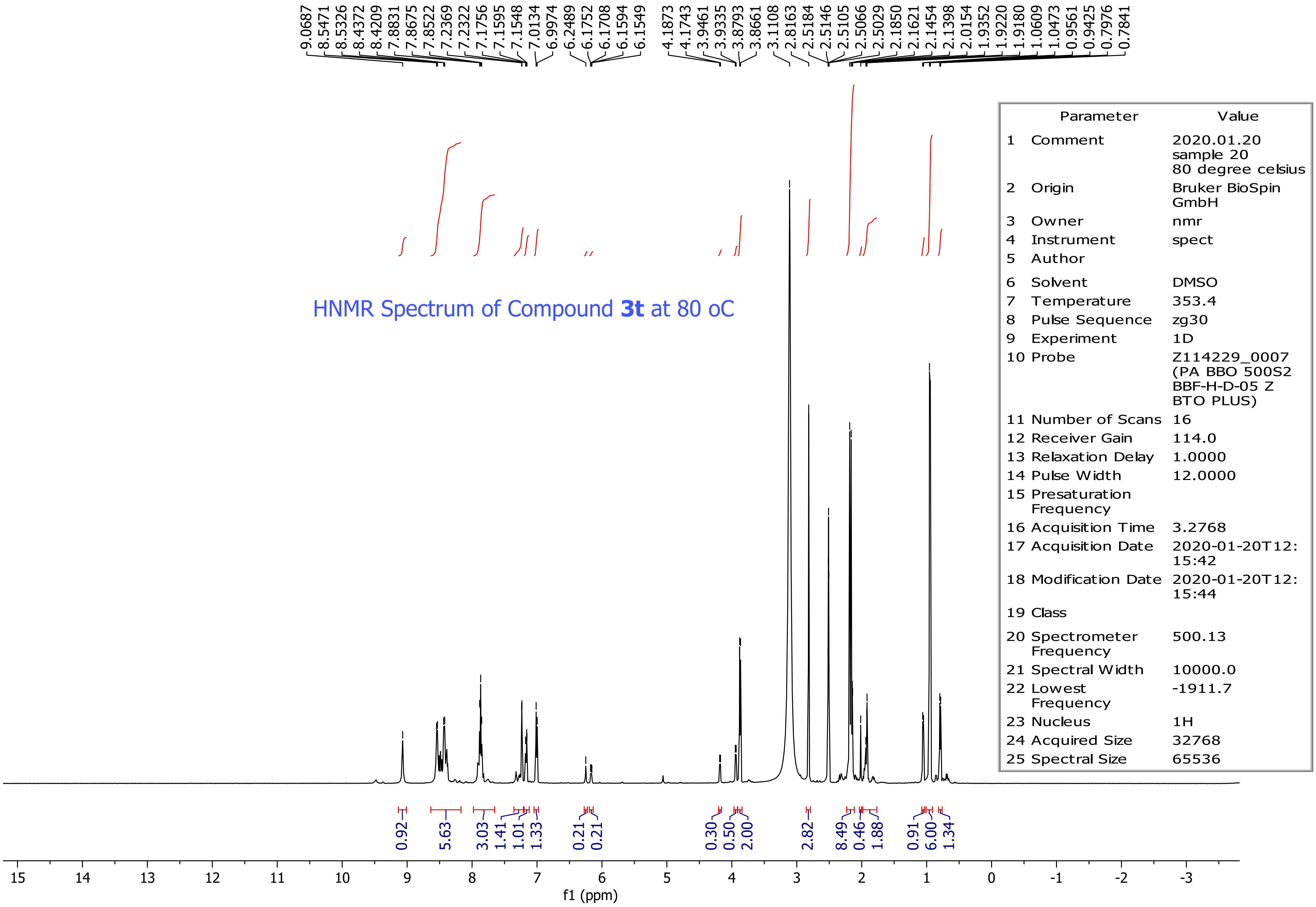

	Parameter	Value
1	Comment	2020.01.20 sample 20 80 degree celsius
2	Origin	Bruker BioSpin GmbH
3	Owner	nmr
4	Instrument	spect
5	Author	
6	Solvent	DMSO
7	Temperature	353.4
8	Pulse Sequence	zg30
9	Experiment	1D
10	Probe	Z114229_0007 (PA BBO 500S2 BBF-H-D-05 Z BTO PLUS)
11	Number of Scans	16
12	Receiver Gain	114.0
13	Relaxation Delay	1.0000
14	Pulse Width	12.0000
15	Presaturation Frequency	
16	Acquisition Time	3.2768
17	Acquisition Date	2020-01-20T12:15:42
18	Modification Date	2020-01-20T12:15:44
19	Class	
20	Spectrometer Frequency	500.13
21	Spectral Width	10000.0
22	Lowest Frequency	-1911.7
23	Nucleus	1H
24	Acquired Size	32768
25	Spectral Size	65536

CNMR Spectrum of Compound **3t**

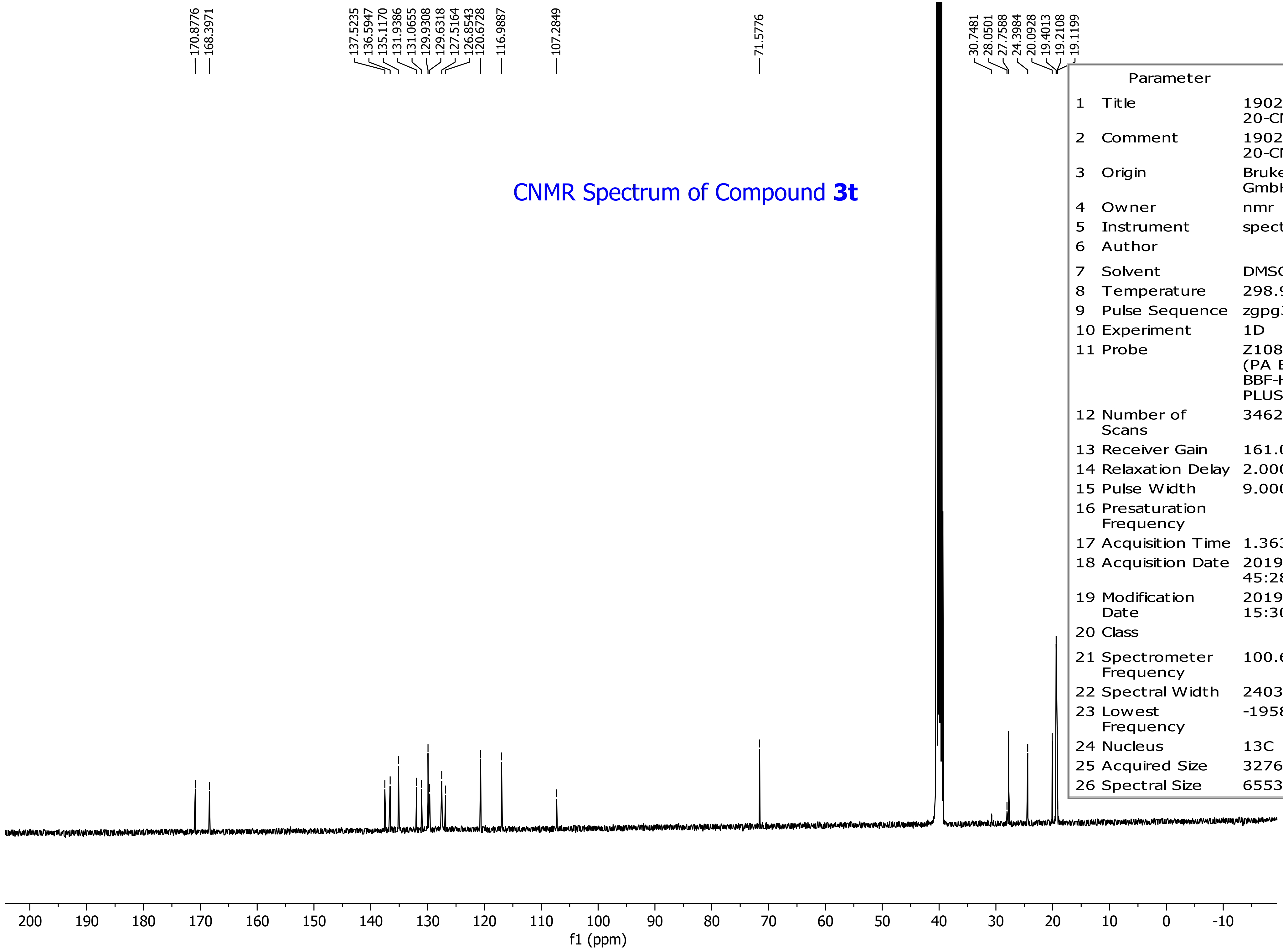

	Parameter	Value
1	Title	190225-Sample 20-CNMR.10.fid
2	Comment	190225-Sample 20-CNMR
3	Origin	Bruker BioSpin GmbH
4	Owner	nmr
5	Instrument	spect
6	Author	
7	Solvent	DMSO
8	Temperature	298.9
9	Pulse Sequence	zgpg30
10	Experiment	1D
11	Probe	Z108618_0166 (PA BBO 400S1 BBF-H-D-05 Z PLUS)
12	Number of Scans	3462
13	Receiver Gain	161.0
14	Relaxation Delay	2.0000
15	Pulse Width	9.0000
16	Presaturation Frequency	
17	Acquisition Time	1.3631
18	Acquisition Date	2019-02-25T14:45:28
19	Modification Date	2019-02-25T20:15:30
20	Class	
21	Spectrometer Frequency	100.62
22	Spectral Width	24038.5
23	Lowest Frequency	-1958.4
24	Nucleus	13C
25	Acquired Size	32768
26	Spectral Size	65536
